# 1,2,6-Thiadiazinones as Novel Narrow Spectrum Calcium/Calmodulin-Dependent Protein Kinase Kinase 2 (CaMKK2) Inhibitors

**DOI:** 10.3390/molecules23051221

**Published:** 2018-05-19

**Authors:** Christopher R. M. Asquith, Paulo H. Godoi, Rafael M. Couñago, Tuomo Laitinen, John W. Scott, Christopher G. Langendorf, Jonathan S. Oakhill, David H. Drewry, William J. Zuercher, Panayiotis A. Koutentis, Timothy M. Willson, Andreas S. Kalogirou

**Affiliations:** 1 Structural Genomics Consortium, UNC Eshelman School of Pharmacy, University of North Carolina at Chapel Hill, Chapel Hill, NC 27599, USA; david.drewry@unc.edu (D.H.D.); william.zuercher@unc.edu (W.J.Z.); tim.willson@unc.edu (T.M.W.); 2 Department of Pharmacology, University of North Carolina at Chapel Hill, NC 27599, USA; 3 Structural Genomics Consortium, Universidade Estadual de Campinas-UNICAMP, Campinas, São Paulo 13083-886, Brazil; phgodoi@yahoo.com (P.H.G.); rafaelcounago@gmail.com (R.M.C.); 4 Center for Molecular and Genetic Engineering (CBMEG), University of Campinas (UNICAMP), Cidade Universitária Zeferino Vaz, Avenida Cândido Rondon 400, P. O. Box 6010, 13083-875 Campinas, São Paulo 13083-886, Brazil; 5 School of Pharmacy, Faculty of Health Sciences, University of Eastern Finland, 70211 Kuopio, Finland; tuomo.laitinen@uef.fi (T.L.); 6 St Vincent’s Institute and Department of Medicine, University of Melbourne, 41 Victoria Parade, Fitzroy 3065, Australia; jscott@svi.edu.au (J.W.S.); clangendorf@svi.edu.au (C.G.L.); joakhill@svi.edu.au (J.S.O.); 7 Mary MacKillop Institute for Health Research, Australian Catholic University, 215 Spring Street, Melbourne 3000, Australia; 8 The Florey Institute of Neuroscience and Mental Health, Parkville 3052, Australia; 9 Lineberger Comprehensive Cancer Center, University of North Carolina at Chapel Hill, Chapel Hill, NC 27599, USA; 10 Department of Chemistry, University of Cyprus, P. O. Box 20537, 1678 Nicosia, Cyprus; koutenti@ucy.ac.cy; 11 Department of Life Sciences, School of Sciences, European University Cyprus, 6 Diogenis Str., Engomi, P. O. Box 22006, 1516 Nicosia, Cyprus

**Keywords:** thiadiazinone, hinge binder, kinase inhibitor design, kinase water network, CaMKK2

## Abstract

We demonstrate for the first time that 4*H*-1,2,6-thiadiazin-4-one (TDZ) can function as a chemotype for the design of ATP-competitive kinase inhibitors. Using insights from a co-crystal structure of a 3,5-bis(arylamino)-4*H*-1,2,6-thiadiazin-4-one bound to calcium/calmodulin-dependent protein kinase kinase 2 (CaMKK2), several analogues were identified with micromolar activity through targeted displacement of bound water molecules in the active site. Since the TDZ analogues showed reduced promiscuity compared to their 2,4-dianilinopyrimidine counter parts, they represent starting points for development of highly selective kinase inhibitors.

## 1. Introduction

**Figure 1:**
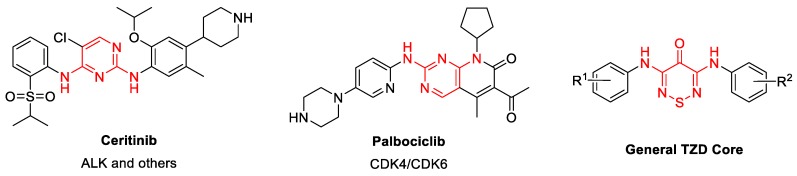
Representative examples of known dianilino(amino)pyrimidines (highlighted in red) and general (TDZ) core.

Protein kinases catalyze phosphate transfer from Adenosine Triphosphate (ATP) to tyrosine, threonine or serine residues in specific target proteins. These phosphorylation events occur in almost every signal transduction pathway and provide regulatory points for therapeutic intervention [1]. Kinases have been successfully utilized as drug targets for the past 30 years, with 38 kinase inhibitors approved by the FDA to date [2]. These drugs are predominantly multi-targeted tyrosine kinase inhibitors for the treatment of cancer [3]. However, approval of kinase inhibitors for the treatment on non-oncological diseases, such as rheumatoid arthritis, psoriasis and lung fibrosis has demonstrated their broader utility in treatment of human disease. There are over 500 kinases in the human genome, suggesting that there remains an untapped potential to treat a wide range of human ailments with new classes of inhibitors. Large scale kinome-wide profiling of ATP-competitive kinase inhibitors has also started to uncover the preferred chemotypes for the inhibition of many of the relatively under-studied kinases or dark kinases [4,5,6]. Despite the success in development of kinase inhibitor drugs, there is a still a need for new heterocycles on which to build ATP-competitive inhibitors [7,8]. One chemotype that has not yet been used in kinase inhibitor design is the 4*H*-1,2,6-thiadiazin-4-one (TDZ, Figure 1) [9,10] that can be prepared from 2,2-dichloromalononitrile [11].

Dianilinopyrimidines represent a remarkably common chemotype that is found in ~10% of the clinically approved kinase inhibitor drugs, including ceritinib and palbociclib (Figure 1) [12]. Each of these drugs demonstrates potency and efficacy for its primary kinase target but also has cross activity on a broad range of other kinases. As such, these drugs and many other pyrimidine-based inhibitors have limited use as chemical probes to study the biology of specific kinases. As further testament to the broad activity profile of the dianilinopyrimidine chemotype, the 35 examples that are contained in the chemogenomic inhibitor sets PKIS/PKIS2 showed activity on >400 different protein kinases (excluding mutants) in either enzyme inhibition or affinity capture assays [13,14].

## 2. Results

### 2.1. Synthesis

Following analysis of the kinome-wide profiling of the dianilinopyrimidines in PKIS/PKIS2, we selected five R^1^ and R^2^ substituent pairs (Table 1 and Figure S1) that showed the broadest range of activity on human kinases. The corresponding dianilino-TDZs (**1**–**5**) were synthesized in two-steps from 3,5-dichloro-4*H*-1,2,6-thiadiazin-4-one (**6**) [9]. The reason for this strategy is that the first of the two reactive chlorine atoms of dichlorothiadiazinone **6** can be readily displaced by anilines and alkylamines with stoichiometric amounts of the amine (1 equiv.) and 2,6-lutidine (1 equiv.) as base in EtOH, at *ca.* 0–20 °C. However, more forcing conditions are typically required for the displacement of the remaining chloride. This is owed to electron release by the 3-amino group into the thiadiazine that decreases the electrophilicity of the 3-amino-5-chloro-1,2,6-4*H*-thiadiazin-4-one [15]. Nevertheless, we were able to use our recently developed Buchwald-Hartwig coupling conditions to overcome this difficulty [16] and enable the efficient synthesis of unsymmetrical 3,5-diamino-thiadiazinones.

**Scheme 1:**
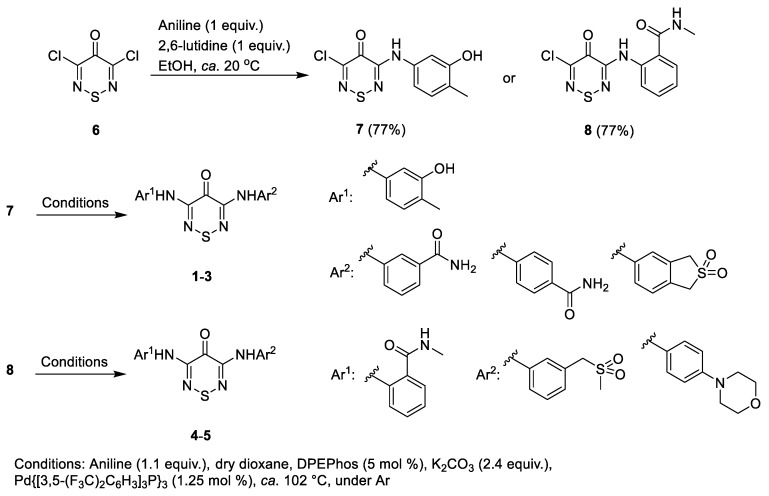
Synthetic route to dianilino-TDZs **1**–**5**.

Therefore, treatment of dichlorothiadiazinone **6** with one equivalent of 5-amino-2-methylphenol or 2-amino-*N*-methylbenzamide in the presence of 2,6-lutidine (1 equiv.), gave the required 3-chloro-5-[(3-hydroxy-4-methylphenyl)amino]-4*H*-1,2,6-thiadiazin-4-one (**7**) and 2-[(5-chloro-4-oxo-4*H*-1,2,6-thiadiazin-3-yl)amino]-*N*-methylbenzamide (**8**), respectively in good yields with a chromatography-free work-up (Scheme 1). Subsequently, scaffolds **7** and **8** were subjected to a Pd-catalyzed Buchwald-Hartwig coupling to introduce the second aniline. The five desired products (**1**–**5**) were obtained in medium to good yields (66–94%, Scheme 1).

After the synthesis of the desired dianilino-TDZs (**1**–**5**), the stability of dianilinothiadiazinone in biological systems was assessed: 3,5-bis(phenylamino)-4*H*-1,2,6-thiadiazin-4-one (**9**) [9] was subjected to neutral, acidic or slightly basic aqueous conditions (H_2_O/DMSO 50:50, THF/HCl 2 M 50:50 or THF/H_2_O 50:50 at pH 9 with a carbonate buffer), presence of amine or thiol nucleophiles (BuNH_2_ 1 equiv., PhNH_2_ 1 equiv., PhSH 1 equiv.), oxidizing [2,3-dichloro-5,6-dicyano-1,4-benzoquinone (DDQ) 2 equiv.] and reducing conditions (Sn, 2 equiv.). The dianilinothiadiazine **9** was stable (by TLC) to all the above conditions after 48 h indicating that the thiadiazinone core was stable for potential development.

### 2.2. Initial Kinase Profiling

The corresponding dianilino-TDZs (**1**–**5**) were tested on a panel of 46 protein kinases representing the major kinome branches at a concentration of 10 μM using a Differential Scanning Fluorimetry (DSF) [17]. Only analogues **1**–**3** induced a significant (>2 °C) thermal shift when incubated with a subset of the proteins (Table 1). Compound **1** showed significant activity only on the pseudokinase TRIB2 (ΔT_m_ = 2.5 °C) [18]. Compound **2** showed the broadest activity profile with ΔT_m_ > 2 °C on 16/46 kinases. Compound **3** showed an intermediate activity profile, ΔT_m_ > 2 °C on 9/46 kinases. Compounds **4** and **5** did not show ΔT_m_ > 2 °C on any of the 46 kinases, although weak activity was detected on TRIB2 (ΔT_m_ ~1 °C).

### 2.3. CaMKK2 Crystallography

**Figure 2:**
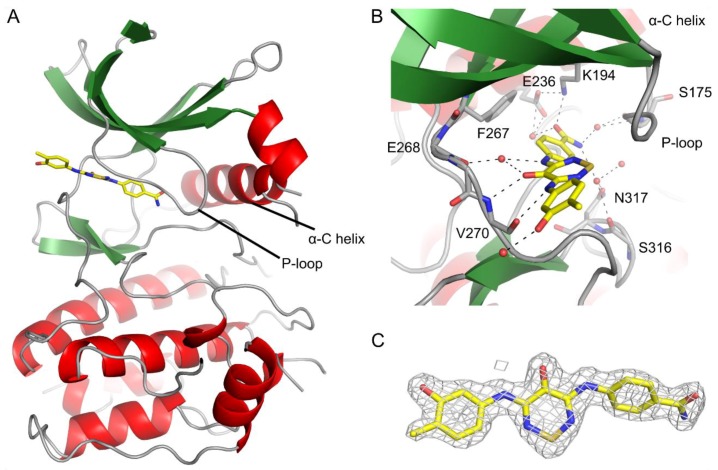
The co-crystal structure of CAMKK2 bound to compound **2**. (**A**) Cartoon representation of CAMKK2. (**B**) Binding interactions between CAMKK2 and compound **2**. Dashed lines depict putative hydrogen bonds. Water molecules are shown as red spheres. (**C**) Electron density (omit) map (shown as a grey mesh contoured at 1.5 σ) for compound **2**.

To investigate the molecular details of interaction of the dianilino-TDZs (**1**–**3**) with protein kinases, co-crystallization with the corresponding purified proteins was attempted. Diffracting crystals were obtained with compound **2** in complex with CAMKK2 (see Table S2). The structure was solved by molecular replacement. The CAMKK2 kinase domain adopted an active state conformation in which residues of the regulatory and catalytic spines were aligned (Figure 2A); residue Glu236 within α-C helix directly contacted Lys194 (“α-C helix in”); and residues Asp330 and Phe331 within the conserved DFG motif pointed towards and away, respectively, from the ATP-binding site (“DFG -in”).

The ligand displayed two direct contact points to the hinge region of the ATP-binding pocket: one involving the oxygen atom of the thiadiazinone moiety and the other through the nitrogen atom of the hydroxymethylaniline moiety (Figure 2B). A water bridge made by the nitrogen atom from the aminobenzamide provided a third contact point to the kinase hinge region. The co-crystal structure revealed that the oxygen atom of the aminobenzamide interacted with the catalytic Lys194 and made a water-bridge with Glu236 of the α-C helix and Asp330 within the conserved DFG motif. Likewise, two water-bridge interactions connected the nitrogen atom from the aminobenzamide moiety and residues Ser175 within P-loop and Asn317 at the bottom of the kinase ATP-binding site. Finally, the ligand aminobenzamide ring made a T-shaped π-π interaction with the Phe267. Compound **2** is bound to CAMKK2 with aniline groups in a twisted conformation relative to the central TDZ ring, as can be seen in the electron density map (Figure 2C).

### 2.4. CaMKK2 Docking and Water Map Simulation

**Figure 3:**
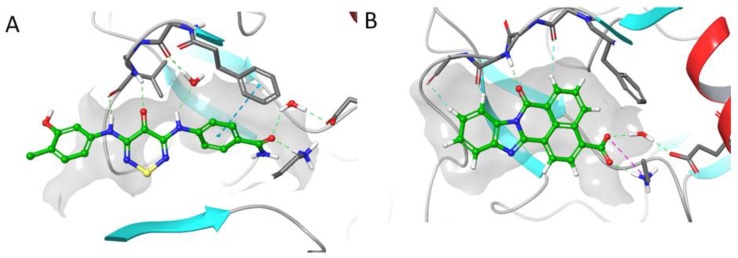
Comparison of the interaction of compounds **2** and STO-609 with the hinge region of CAMKK2: (**A**) PDB:5VT1 with compound **2**; (**B**) PDB:2ZV2 with STO-609.

To further probe the molecular basis of ligand binding to CAMKK2, we compared our co-crystal structure of compound **2** (PDB 5VT1) with the previously published co-crystal structure with STO-609 (PDB:2ZV2) (Figure 3) [19]. We also controlled for hinge contacts in the model by using the 2,4-dianilinopyrimidines as a training set (see Figure S2). We noted that STO-609 can displace a bound water molecule from the ATP-binding site that is still present when compound **2** is bound to the enzyme. The carboxylic acid of STO-609 also forms a tighter interaction with the catalytic lysine than the benzamide of compound **2**. To improve its activity, we designed analogues of **2** that would be able to displace the bound water molecule and form stronger interactions with the catalytic lysine.

**Figure 4:**
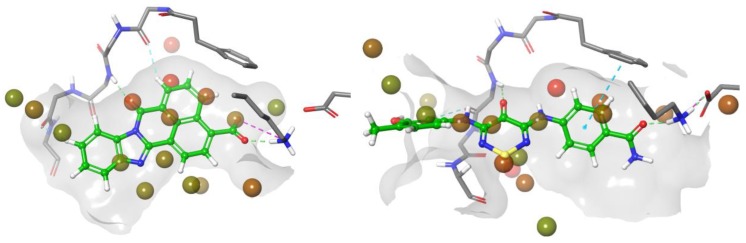
Hydration sphere of STO-609 (left) and **2** (right) generated by Water Map simulation showing high and low energy waters with graded shading (red—high energy and green—low energy).

In order to use molecular docking to guide the design of new analogues, we used the Maestro suite (2017-3) to create a models of compounds STO-609 and **2** bound to CaMKK2 that accurately reflected what was seen in the crystal structures of both [19]. Water Map using a 2 nS simulation was used to populate the hydration sphere of compounds STO-609 and **2**. The resulting model correctly identified the crystallographically observed water molecule highlighted in red (Figure 4). In addition, several other water molecules were identified in the ‘back’ pocket of the CaMKK2 that contained the catalytic lysine. Several analogues of compound **2** were designed to directly displace/interact with the key water molecule. In parallel, we designed a series of analogues to strengthen the interaction with the catalytic lysine.

**Figure 5:**
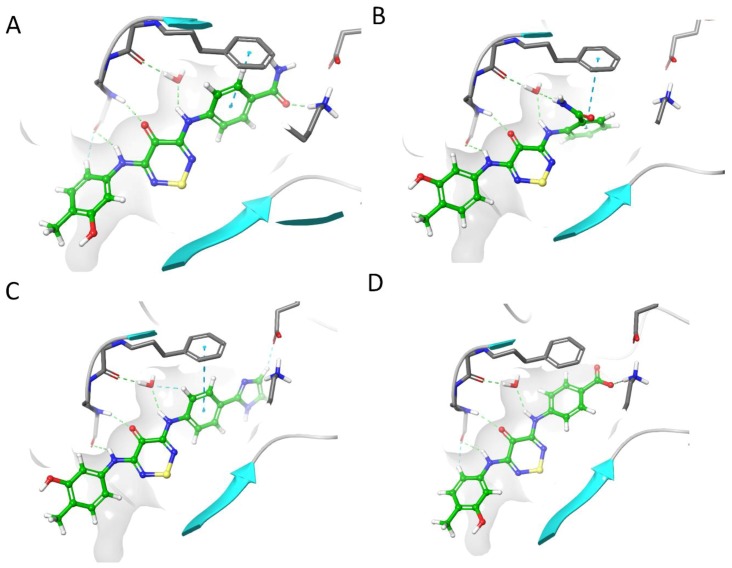
Molecular docking into CaMKK2 of (**A**) compound **2**; (**B**) compound **10**; (**C**) compound **11**; (**D**) compound **12.**

The docking simulations showed that strengthening the interaction with the catalytic lysine while retaining the bound water molecule gave better scores than direct displacement of the water (Figure 5). We first optimized the core to see if the weak hit (**2**) could be a tractable starting point. Molecular simulations of compound **2** (Figure 5A) showed a weaker interaction with the catalytic lysine when the primary amide was switched in compound **10** (Figure 5B); the interaction with the water produced a more effective docking pose with a strong water mediated interaction with the backbone. We were able to boost the proposed lysine interaction with an imidazole substitution (**11**) (Figure 5C). The carboxylic acid in STO-609 appeared to contribute significantly to the binding affinity to CaMKK2. We designed a switch of the primary amide in compound **2** to a carboxylic acid (**12**) (Figure 5D) and this gave a 14/15 poses match to where STO-609’s carboxylic acid was directed in the co-crystal structure with CaMKK2 [19].

### 2.5. Optimisation Results on CaMKK2

#### 2.5.1. Outline of Compounds

**Figure 6:**
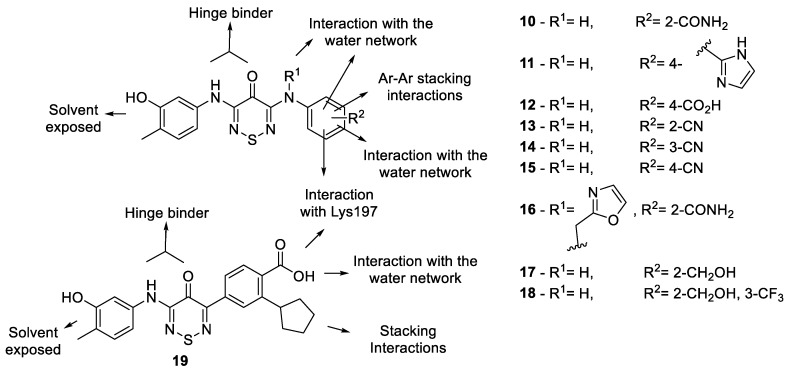
Designed compound rationale.

We proposed a series of modifications of compound **2** relating to the crystal structure and modelling (Figure 6). These included a switch of the *para*-amide to the *ortho*-position (**10**) to better interact with the water and a direct substitution of the *para*-amide with a carboxylic acid (**12**) to form a stronger interaction with the water network. A series of mono-substituted *ortho*-, *meta*- and *para*- cyano analogues (**13**–**15**, respectively) probed the space available in this pocket and checked conformation constraints. A substitution on the adjacent anilino-nitrogen to the hinge binder to directly replace the water with a 4-methyloxazole (**16**) was encouraging. A methanol substitution at the *ortho* position (**17**) also looked promising and to increase the *π*-stacking potential of this analogue we added a *meta*-trifluoromethyl group (**18**). The use of an imidazole as a hydrogen bond donor/acceptor and the model (Figure 4C) suggested that, though out of plane, it could align with the catalytic lysine. The *para*-substituted imidazole (**11**) is about 30–40° out of plane and looked favorable to form a networked interaction between Lys197 and the wider water network. The final analogue was an arylthiadiazinone with a substitution of 2-cyclopentylbenzoic acid (**19**). This tactic for interaction of the wider water network with the *para*-carboxylic acid while having an adjacent *meta*-cyclopentyl to form a *π*-stacking/lipophilic interaction was previously used to successfully target SGK1, a regulator of epithelial sodium channels (eNaCs) [20].

#### 2.5.2. Analogue Synthesis

**Scheme 2:**
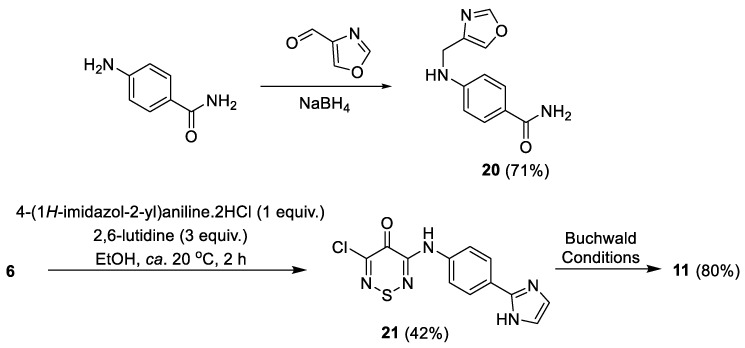
Synthesis of oxazole amine **20** and dianilino-TDZ **11**.

The designed analogues **10**–**19** were prepared using 3-chloro-5-[(3-hydroxy-4-methylphenyl)-amino]-4*H*-1,2,6-thiadiazin-4-one (**7**) as the substrate for the Buchwald-Hartwig coupling reaction to the relevant aniline. The desired dianilino-TDZs were prepared in medium to good yields (65–94%) except for 4-benzoic acid derivative **19** that gave a low yield (36%) attributed to potential nucleophilic displacements by the carboxylate ion in the reaction conditions (K_2_CO_3_). The secondary amine **20** required for the preparation of oxazole derivative **16** was prepared by a reductive amination reaction of 4-aminobenzamide and oxazole-4-carbaldehyde with sodium borohydride (Scheme 2).

Interestingly, a different route was used to access imidazole derivative **11** as the Buchwald-Hartwig coupling of thiadiazinone **7** with 4-(1*H*-imidazol-2-yl)aniline led to a complex mixture of products. This was resolved by switching the reaction sequence and performing first the nucleophilic displacement of the 3-chloride of dichlorothiadiazinone **6** with 4-(1*H*-imidazol-2-yl)aniline to afford anilinothiadiazine **21** and subsequently performing the Buchwald-Hartwig coupling with 5-amino-2-methylphenol (Scheme 2).

**Scheme 3:**
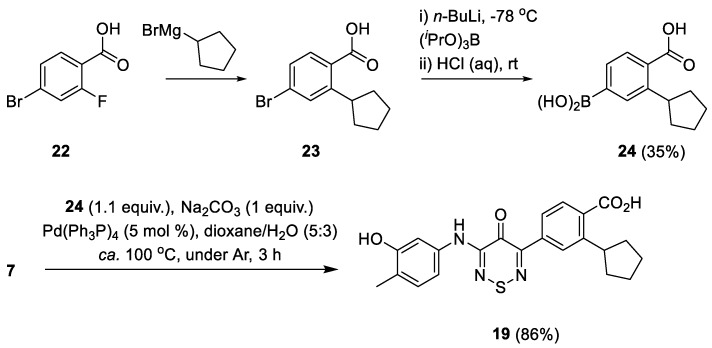
Synthesis of boronic acid **22** and aryl-anilino-TDZ **19**.

The arylthiadiazinone analogue **19** required a different synthetic protocol involving a Suzuki coupling with the relevant arylboronic acid **22**. The boronic acid was prepared in two steps from 4-bromo-2-fluorobenzoic acid (Scheme 3). Treatment of 4-bromo-2-fluorobenzoic acid **22** with cyclopentyl magnesium bromide led to 4-bromo-2-cyclopentylbenzoic acid (**23**). Subsequent lithium-halogen exchange and treatment with triisopropyl borate gave the desired boronic acid **24** albeit in a 35% overall yield. Boronic acid **24** was then reacted with 3-chloro-5-[(3-hydroxy-4-methylphenyl)-amino]-4*H*-1,2,6-thiadiazin-4-one (**7**) in the presence of Pd(Ph_3_P)_4_ (5 mol %) to yield the aryl-thiadiazinone **19** in 86% yield (Scheme 3) [10]. We note that this reaction order was chosen since it is difficult to perform a mono-arylation Suzuki reaction [10] but easy to mono-displace dichlorothiadiazinone **6** with amine nucleophiles.

### 2.6. Optimization Results on CaMKK2

**Figure 7:**
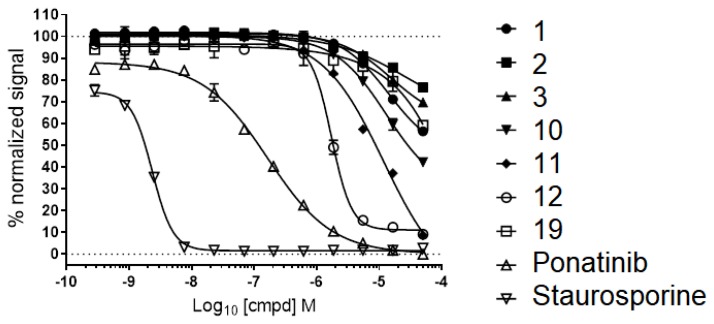
TR-FRET optimization of compound **2** with ponatinib and staurosporine as a control.

To more accurately determine the relative changes in potency of the TDZ analogues and to enable measurement of accurate IC_50_^’^s, we developed a Time-Resolved Fluorescence Resonance Energy Transfer (TR-FRET) assay. The TR-FRET tracer displacement assays were generated using a protocol derived from the Lanthascreen binding assays (ThermoFisher Scientific, Waltham, MA, USA) [21]. In this assay, we measured the effect of ATP competing compounds that are able to displace a fluorophore-labeled pan-kinase inhibitor (tracer 236) from the ATP binding site. We used ponatinib and staurosporine as internal controls to calibrate the FRET assay. The results of the CAMKK2 FRET assay are shown in Figure 7 and Table 2 (see Figures S3 and S4, SI). Surprisingly, TDZ analogues **1** and **2** showed no measurable activity at a concentration up to 50 μM but analogue **3** gave weak activity with an IC_50_ 34 μM. Nevertheless, several of the structure-optimized analogues showed improvements in potency. TDZ’s **10**–**12** had the highest affinity for CAMKK2 with IC_50_ 7.8, 3.2 and 10.5 μM, respectively.

The switch of the amide from the *para*-position (**2**) to the *ortho*-position (**10**) provided a >8-fold boost likely related to the new more favorable water mediated interaction. The exchange of the *para*-amide (**2**) to the *para*-carboxylic acid (**12**) led to a >5-fold increase in potency. However, the mono-cyano group substitutions were relatively in-effective. The *ortho*-cyano (**13**) showed some activity (43 μM) likely do to the water network interaction but *meta*-cyano (**14**) and *para*-cyano (**15**) were >50 μM against CaMKK2. This was the same result for the 4-methyloxazole (**16**), which was surprising but could be related to an inability to be accommodated in the active site. The methanol analogues (**17**-**18**) were also effectively inactive, likely due in part to the lack of ability to reach the water interaction. The imidazole (**11**) preformed well as we expected from our model (Figure 4C) and appeared to form the water network interaction in the back pocket of CaMKK2. The direct carbon-carbon bonded *para*-carboxylic acid with adjacent *meta*-cyclopentyl compound (**19**) was only weakly active (38 μM) and was likely out of position on this scaffold to form the optimal interaction as in STO-609.

### 2.7. Advanced Enzyme Assay Results on CaMKK2 Demonstrating Functional Inhibition

To further characterize the activity of **10**–**12** as inhibitors of CAMKK2, the compounds were subjected to an enzyme inhibition assay. CaMKK2 activity was measured by determining the rate of transfer of radiolabeled phosphate from [*γ*-^32^P]-ATP to a synthetic peptide substrate [22]. Compounds **10**–**12**, when initially screened at a concentration of 1 *µ*M, showed statistically significant inhibition of CAMKK2 kinase activity. The compounds were then screened at 7 concentrations (see Table S3, SI) to produce moderately potent IC_50_’s. TDZs **10**–**12** were demonstrated to be competent inhibitors of the CaMKK2 enzyme with enzymatic IC_50_^’s^ of 11.9, 6.5 and 4.1 μM, respectively (Table 3).

## 3. Discussion

We demonstrate, for the first time, that the 4*H*-1,2,6-thiadiazin-4-one (TDZ) chemotype can function as an ATP-competitive kinase inhibitor. TDZ represents a novel hinge binder with the potential to be further optimized into a high quality chemical probe for kinases such as CaMKK2. Furthermore, we report the first protein co-crystallization with this rare heterocycle. The electronics of the TDZ core allows for participation of the sulfur atom to be part of extended conjugated electronic exchanges through the core units to transfer charge [23]. This electronic property, exploited in solar cell applications, can partly explain the general lack of kinome promiscuity compared to the dianilinopyrimidine. The modular synthesis and relative narrow kinome spectrum make the TDZ an attractive chemotype for further development.

CaMKK2 is predominantly expressed in the brain, with trace expression in peripheral tissues such as the testis, spleen and lung [24,25]. In addition to recently being linked to appetite in vivo [26], CAMKK2 is over-expressed in multiple cancers [27,28]. The knockout of CaMKK2 can reduce cell proliferation and tumorigenicity in vivo, making CAMKK2 an attractive drug target. The only reported potent small molecule inhibitor of CaMKK2 is STO-609, which has several liabilities limiting its use as a probe of CaMKK2 activity. These include poor solubility and unfavorable off-target kinome profile with kinases that would cloud the interpretation of a phenotype including ERK8, MNK1 and PIM3 [29,30,31]. In addition, STO-609 is an agonist of the arylhydrocarbon receptor (AhR) [32]. The complicating factors highlight the need for the design and development of high quality inhibitors targeting CAMKK2.

Our results add further credence to the importance of water networks in optimization of kinase inhibitors. The advent of powerful modelling tools such as Water Map and the Schrodinger Maestro platform have made manipulating the water network more accessible [33,34,35]. There are two distinct water network regions that the TDZ core can exploit in binding to CaMKK2. We have shown an ability to exploiting the water network and lysine interactions, we improved on the activity of compound **2** and produced compound **11** that is >15-fold more potent. Our discovery of the TDZ core as a useful chemotype for kinase inhibitor design adds a new a hinge binding heterocycle to the medicinal chemistry tool box and provides another example of the application of sulfur in drug design.

## 4. Materials and Methods

### 4.1. Kinase Panel

Kinase selectivity assay—A home-made kinase panel was generated for the following enzymes: AAK1, BMP2K, BMX, BRAF, CAMK1D, CAMK1G, CAMKK1, CAMKK2B, CDC42BPA, CDK2, CDKL1, CHEK2, CLK1, CSNK1G1, CSNK1G3, CSNK2A1, DYRK1A, DYRK2A, EPHA2, GAK, GSG2, MAPK1, MAP2K7, MAPK14B, MAPK3, PHKG2, PIM1, PLK1, PKMYT1, PRPF4B, RPS6KA1A, RPS6KA5A, RPS6KA6A, SLK, SRPK1, SRPK2, STK3, STK6, STK10, STK17A, STK24, STK38L, TRIB2, TTK, VRK1 and VRK2. Proteins were produced in *E. coli*, purified in a Ni-chelate column, followed by overnight digestion using TEV protease (made in house with an *N*-terminal 6xHis tag) and dialysis to remove imidazole. To clear samples of uncleaved proteins and the TEV protease, samples were loaded on new Ni-chelate columns. The flow through was collected, concentrated and loaded to a HiLoad Superdex 200 16/600 column (GE Healthcare, Chicago, IL, USA) for final polishing and buffer exchange.

Starting from 100 *µ*M protein stocks, our kinase panel enzymes were diluted to 1 *µ*M in buffer 100 mM K_2_HPO_4_ pH 7.5 containing 150 mM NaCl, 10% glycerol and 5X dye (Applied Biosystems catalogue 4461806). The protein/dye mixture was transferred to a 384-well PCR microplate having 20 *µ*L per well. Compounds in DMSO at 10 mM concentration were added in 20 nL volume, using a liquid handling device setup with a pin head, to make 10 *µ*M compound concentration in the assay plate.

Protein thermal shift data was measured in a qPCR instrument (Applied Biosystems QuantStudio 6) programmed to equilibrate the plate at 25 °C for 5 min followed by ramping the temperature to 95 °C at a rate of 0.05 °C/s. Data was processed using Protein Thermal shift software (Applied Biosystems) by fitting experimental curves to a Boltzmann function to calculate differential thermal shifts (dT_m_) referenced to protein/dye in 0.2% DMSO.

### 4.2. CaMKK2 Crystallization

#### 4.2.1. Cloning, Protein Expression and Purification

The crystallization of CAMKK2 was performed with a construct of CAMKK2 isoform 7 residues 161-449 (NCBI NP_001257415.1 – SGC construct CAMKK2B-cb002) containing the wild-type kinase domain in vector pNIC28-Bsa4. The construct was transformed into BL21(DE3) *Escherichia coli* cells that co-express *λ*-phosphatase and three rare tRNAs (plasmid pACYC-LIC+). Cells were cultured in TB medium containing 50 µg/mL kanamycin and 35 µg/mL chloramphenicol at 37 °C with shaking until the OD600 reached ~3 and then cooled to 18 °C for 1 h. Isopropyl *β*-d-1-thiogalactopyranoside (IPTG) was added to a final concentration of 0.1 mM and the cultures were left overnight at 18 °C. The cells were collected by centrifugation then resuspended in 2× lysis buffer [1× lysis buffer is 50 mM HEPES buffer, pH 7.5, 0.5 M KOAc, 10% (*v*/*v*) glycerol, 50 mM each arginine/glutamate, 10 mM imidazole, 1.0 mM tris(2-carboxyethyl)phosphine (TCEP), Protease Inhibitor Cocktail Set VII (Calbiochem, 1/500 dilution)] and flash-frozen in liquid nitrogen. Cells were lysed by sonication on ice. The resulting proteins were purified using Ni-Sepharose resin (GE Healthcare) and eluted stepwise in 1× lysis buffer with 300 mM imidazole. Removal of the hexahistidine tag was performed at 4 °C overnight using recombinant TEV protease. The protein was further purified using reverse affinity chromatography on Ni-Sepharose followed by gel filtration (Superdex 200 16/60, GE Healthcare). The protein in gel filtration buffer (10 mM HEPES, 500 mM KOAc, 1.0 mM TCEP, 5% (*v*/*v*) glycerol, 50 mM each arginine/glutamate) was concentrated to 8.5 mg/mL (measured by UV absorbance in a NanoDrop spectrophotometer (Thermo Scientific, Waltham, MA, USA) using the calculated molecular weight and estimated extinction coefficient) using 30 kDa molecular weight cut-off centrifugal concentrators (Sigma-Aldrich Corp., St. Louis, MO, USA) at 4 °C. The concentrated protein was flash-frozen in a liquid nitrogen bath and stored at −80 °C until use.

#### 4.2.2. Protein Crystallization

Kinase inhibitor (dissolved in 100% DMSO) was added to the protein in 3-fold molar excess and incubated on ice for approximately 30 min. The mixture was centrifuged at 15,000 rpm for 10 min at 4 °C before setting up 150 nL volume sitting drops at three ratios (2:1, 1:1, or 1:2 protein-inhibitor complex to reservoir solution). Crystallization experiments were performed at 20 °C. Crystals were cryoprotected in mother liquor supplemented with 25–30% glycerol before flash-freezing in liquid nitrogen for data collection. Diffraction data were collected at 100 K at Diamond Light Source beamline I03. Crystal optimization used Newman’s buffer system [36].

#### 4.2.3. Structure Solution and Refinement

Diffraction data were integrated using XDS [37] and scaled using AIMLESS from the CCP4 software suite (version 7.0.057, London, UK) [38]. Molecular replacement (MR) was performed with Phaser [19] using the CAMKK2 bound to STO-609 co-structure (PDB ID 2ZV2) [19]. Automated refinement was performed with Refmac [39,40]. Coot [41] was used for manual model building and refinement. Structure validation was performed using MolProbity [42]. Structure factors and coordinates have been deposited in the PDB (see Table S2, SI).

### 4.3. Molecular Modelling

#### 4.3.1. Molecular Modelling

Molecular modelling was performed using Schrödinger Maestro software package (version 2018-1, Schrödinger, Mannheim, Germany) [43]. Structures of small molecules were prepared using and the LigPrep module of Schrodinger suite employing OPLS3 force for all computations. X-ray crystal structure for the CaMMKK2 (PDB:5VT1/2ZV2) was pre-processed using the protein preparation wizard of Schrödinger suite to optimize the hydrogen bonding network [43].

Prior to Glide docking, the grid box was centered using corresponding x-ray ligand as template. The ligand docking was performed using default SP settings of Schrodinger Glide with softened vdw’s potential (0.6) and additional hydrogen bond constraints to NH of V270 (hinge residue). Graphical illustrations were generated using Schrödinger Maestro software (version 2018-1, Schrödinger, Mannheim, Germany).

#### 4.3.2. Hydration Site Analysis

Hydration site analysis calculated with Water Map (Schrödinger Release 2017-3: Water Map, Schrödinger, LLC, New York, NY, 2017). The 5VT1 structure was prepared with Protein Preparation Wizard (as above). Waters were analyzed within 6 Å of the co-crystallized ligand and the 2 ns simulation was conducted with OPLS3 force field.

### 4.4. Biochemical Assays

#### 4.4.1. CaMKK2B TR-FRET Assay

CAMKK2 kinase domain (132–470) was cloned in a pNIC-Bio2 vector in fusion with *N*-terminal 10xHis tag followed by a TEV protease cleavage site and a C-terminal biotin ligase recognition sequence. This construct was used in the expression of CAMKK2 in *E. coli* BL21(DE3)-R3-BirA [44]. Protein was purified in a Ni-NTA column (Thermo Scientific, Waltham, MA, USA) followed TEV digestion overnight, dialysis to remove imidazole and re-purification in Ni-NTA to remove undigested samples and TEV protease (made in house with an *N*-terminal 6xHis tag). As a last step, this sample was loaded to a HiLoad Superdex 200 16/600 column (GE Healthcare, Chicago, IL, USA) for final polishing and buffer exchange.

Tracer displacement assay was measured in 15 *µ*L volume containing 5 nM of our *C*-terminal biotinylated CAMKK2 kinase domain, 2 nM of Europium-labeled streptavidin in buffer 50 mM HEPES pH 7.5, 10 mM MgCl_2_, 1 mM EGTA, 0.01% Brij-35 and 8 nM of tracer 236 (measured K_D_ of 8.13 ± 0.9 nM) as described [45].

#### 4.4.2. CaMKK2 Enzyme Assay

CaMKK2 activity was measured as described previously [22]. A standard 30 μL assay, 1 ng of recombinant bacterial expressed human CaMKK2 (residues 50–588) was added to assay buffer (50 mM HEPES [pH 7.4], 1 mM DTT, 0.02% (*v*/*v*) Brij-35) containing 200 μM CaMKKtide peptide substrate, 50 μM CaCl_2_, 1 μM calmodulin (Sigma-Aldrich Corp., St. Louis, MO, USA), 50 μM [*γ*-^32^P]-ATP (Perkin Elmer) and 5 mM MgCl_2_, in the presence and absence of different concentrations of small-molecule inhibitors. Reactions were incubated for 10 min at 30 °C, after which they were terminated by spotting 15 μL onto P81 phosphocellulose paper (Whatman, GE Healthcare, Chicago, IL, USA) and washing extensively in 1% phosphoric acid. Radioactivity was quantified by scintillation counting.

### 4.5. Chemistry Experimental Section

#### 4.5.1. General Methods and Materials

All chemicals were commercially available except those whose synthesis is described. Anhydrous Na_2_SO_4_ was used for drying organic extracts and all volatiles were removed under reduced pressure. 1,4-Dioxane was dried by refluxing over CaH_2_. All reaction mixtures and column eluents were monitored by TLC using commercial glass backed thin layer chromatography (TLC) plates (Merck Kieselgel 60 F_254_) [46]. The plates were observed under UV light at 254 and 365 nm. The technique of dry flash chromatography was used throughout for all prep scale chromatographic separations using Merck Silica Gel 60 (less than 0.063 mm). Melting points were determined using a PolyTherm-A, Wagner & Munz, Koefler-Hotstage Microscope apparatus or were determined using a TA Instruments DSC Q1000 with samples hermetically sealed in aluminum pans under an argon atmosphere; using heating rates of 5 °C/min (DSC m.p. listed by onset and peak values). Solvents used for recrystallization are indicated after the melting point. UV spectra were obtained using a Perkin-Elmer Lambda-25 UV/vis spectrophotometer and inflections are identified by the abbreviation “inf.” IR spectra were recorded on a Shimadzu FTIR-NIR Prestige-21 spectrometer with Pike *Miracle* Ge ATR accessory and strong, medium and weak peaks are represented by s, m and w, respectively. ^1^H and ^13^C-NMR spectra were recorded on a Bruker Avance 300 (at 300 and 75 MHz, respectively), or a 500 machine (at 500 and 125 MHz, respectively). Deuterated solvents were used for homonuclear lock and the signals are referenced to the deuterated solvent peaks. APT NMR studies identified quaternary and tertiary carbons, which are indicated by (s) and (d) notations, respectively. MALDI-TOF mass spectra were recorded on a Bruker Autoflex III Smartbeam instrument. Low resolution (EI) mass spectra were recorded on a Shimadzu Q2010 GC-MS with direct inlet probe. 3,5-Dichloro-4*H*-1,2,6-thiadiazin-4-one (**6**) was prepared according to the reported procedure [9].

#### 4.5.2. Preparation of Aniline Starting Materials

*4-[(Oxazol-4-ylmethyl)amino]benzamide (**20**)*. To a stirred solution of 4-aminobenzamide (136 mg, 1.00 mol) in EtOH (5 mL), at *ca.* 20 °C, was added oxazole-4-carbaldehyde (97 mg, 1.00 mmol) in one portion and the mixture was stirred at this temperature for 12 h. Then NaBH_4_ (75.6 mg, 2.00 mmol) and the mixture was stirred for a further 6 h. H_2_O (10 mL) was then added and the mixture stirred for 30 min. The colorless solid formed was then filtered under reduced pressure and washed with EtOH (2 mL), DCM (5 mL) and *n*-hexane (5 mL) to give the title compound **20** (153.6 mg, 71%) as colorless plates, m.p. 161–162 °C (from EtOH); *R*_f_ 0.44 (DCM/MeOH, 90:10); (found: C, 60.78; H, 5.03; N, 19.26. C_11_H_11_N_3_O_2_ requires C, 60.82; H, 5.10; N, 19.34%); *λ*_max_(EtOH)/nm 216 (log ε 3.86), 292 (4.34); *υ*_max_/cm^−1^ 3381 m, 3273 m, 3169 m, 3129 w, 1639 m, 1599s , 1530 s, 1422 m, 1391 s, 1385 m, 1342 m, 1278 w, 1267 m, 1242 w, 1204 m, 1186 m, 1150 s, 1126 m, 1109 s, 1086 m, 1061 s, 1003 m, 922 m, 874 w, 842 m, 828 m, 804 m, 789 m, 775 m, 762 m, 727 m, 702 m; *δ*_H_(500 MHz; CDCl_3_) 8.33 (1H, d, *J* 0.7), 7.97 (1H, d, *J* 0.9), 7.63 (2H, d, *J* 8.7, Ar *H*), 7.55 (1H, br s, N*H*), 6.86 (1H, br s, N*H*), 6.61 (2H, d, *J* 8.8, Ar *H*), 6.57 (1H, dd, *J* 5.9, 5.9, Ar *H*), 4.20 (2H, d, *J* 5.8, C*H*_2_); *δ*_C_(125 MHz; CDCl_3_) 167.9 (s), 152.1 (s), 150.7 (s), 137.7 (s), 136.1 (d), 128.9 (d), 121.3 (s), 111.0 (d), 38.4 (t); *m*/*z* (APCI+) 218 (MH^+^, 59%), 201 (93), 175 (32), 137 (100), 120 (55).

*4-Borono-2-cyclopentylbenzoic acid (**24**).* To a stirred solution of 4-bromo-2-fluorobenzoic acid (**22**) (2.00 g, 9.13 mmol) in THF, at *ca*. 0 °C, under a N_2_ atmosphere, was added a solution of 2 M cyclopentyl magnesium bromide (16 mL, 32 mmol) and the mixture stirred at this temperature for 4.5 h. Then was added slowly 2 M HCl (25 mL) followed by EtOAc (40 mL). The two layers were separated and the organic layer was washed with H_2_O (2 × 20 mL) and then dried (MgSO_4_). The solvent was removed under vacuum to give 4-bromo-2-cyclopentylbenzoic acid (**23**) as a colorless solid (2.10 g, 85%) that was used directly in next step without further purification.

*4-Bromo-2-cyclopentylbenzoic acid (**23**)* (2.10 g, 7.80 mmol) was dissolved in THF (50 mL) and the mixture cooled to −78 °C with stirring. Triisopropyl borate (6.30 mL, 27.3 mmol, 3.5 equiv.) was then added, followed by the slow addition of a solution of *n*-BuLi (hexanes) 2.5 M (13 mL, 31.2 mmol, 4 equiv.). The reaction mixture was slowly warmed to room temperature and stirred for 5 h. Then a solution of 2 M HCl (20 mL) was added and the mixture stirred for 10 min. The mixture was extracted with EtOAc (2 × 25 mL) and the combined organic layers were then stirred with 2.5 M NaOH (30 mL) for 1 h. The layers were separated and the aqueous layer acidified to pH 2-3 with concentrated HCl. The mixture was then extracted by EtOAc (2 × 25 mL), the organic layer dried (Na_2_SO_4_) and the solvent was removed under vacuum. The crude colorless solid was stirred in DCM (10 mL) and filtered to give the title compound **24** (750 mg, 35% overall yield) as a colorless solid, m.p. 162–165 °C; *R*_f_ 0.38 (*n*-hexane/Et_2_O, 50:50); *υ*_max_/cm^−1^ 3215 br (O-H), 2955 w, 2947 w and 2870 w (alkyl C-H), 1692 s, 1678 s, 1503 w, 1441 w, 1366 s, 1333 m, 1302 m, 1248 m, 1213 m, 1188 m, 1144 m, 1113 m, 1072 w, 1044w, 1011 w, 932 w, 903 w, 849 w, 791 m, 716 s; *δ*_H_(500 MHz; DMSO-*d*_6_) 12.86 (1H, br s, O*H*), 8.19 (2H, s, O*H*), 7.87 (1H, s, Ar *H*), 7.62 (1H, d, *J* 7.6, Ar *H*), 7.54 (1H, d, *J* 7.6, Ar *H*), 3.67–3.60 (1H, m, alkyl *H*), 1.98 (2H, br s, alkyl *H*), 1.79 (2H, br s, alkyl *H*), 1.66-1.53 (4H, m, alkyl *H*); *δ*_C_(125 MHz; DMSO-*d*_6_) 169.9 (s), 144.1 (s), 137.1 (s), 133.4 (s), 132.3 (d), 131.0 (d), 127.6 (d), 41.1 (d), 34.4 (t), 25.3 (t); mass spectrometry and elemental analysis data could not be obtained due to compound instability.

#### 4.5.3. Preparation of 3-Amino-Substituted-4*H*-1,2,6-Thiadiazines

*3-Chloro-5-[(3-hydroxy-4-methylphenyl)amino]-4H-1,2,6-thiadiazin-4-one (**7**) (General procedure).* To a stirred solution of 3,5-dichloro-4*H*-1,2,6-thiadiazin-4-one (**6**) (366.0 mg, 2.000 mmol) in EtOH (4 mL), at *ca.* 20 °C, was added 5-amino-2-methylphenol (246.3 mg, 2.000 mmol) in one portion followed by 2,6-lutidine (233 μL, 4.00 mmol) and the mixture was stirred at this temperature until complete consumption of the starting material (TLC, 1 h). The yellow solid formed was then filtered under reduced pressure and washed with EtOH (2 mL), DCM (5 mL) and *n*-hexane (5 mL) to give the title compound **7** (477.1 mg, 77%) as orange needles, m.p. 248–250 °C (from EtOH/THF); *R*_f_ 0.53 (*n*-hexane/*t-*BuOMe, 50:50); (found: C, 44.45; H, 2.97; N, 15.56. C_10_H_8_ClN_3_O_2_S requires C, 44.53; H, 2.99; N, 15.58%); *λ*_max_(DCM)/nm 241 (log *ε* 3.88), 343 (4.34), 408 (3.67); *υ*_max_/cm^−1^ 3383br (O-H), 3341 m and 2922 w (C-H), 1584 s, 1562 s, 1557 s, 1553 s, 1503 m, 1450 w, 1427 m, 1421 m, 1406 w, 1366 w, 1327 w, 1312 w, 1263 m, 1238 m, 1194 w, 1153 m, 1123 s, 999 m, 972 m, 874 m, 862 m, 851 s, 802s , 737 m, 727 s; *δ*_H_(500 MHz; CDCl_3_) 9.91 (1H, s), 9.36 (1H, s), 7.32 (1H, d, *J* 2.0, Ar *H*), 7.06 (1H, dd, *J* 8.1, 2.0, Ar *H*), 7.00 (1H, d, *J* 8.2, Ar *H*), 2.08 (3H, s, C*H*_3_); *δ*_C_(125 MHz; CDCl_3_) 157.0 (s), 155.1 (s), 150.0 (s), 140.7 (s), 136.2 (s), 130.1 (d), 120.0 (s), 111.8 (d), 107.4 (d), 15.5 (q); *m*/*z* (MALDI-TOF) 272 (MH^+^ + 2, 42%), 270 (MH^+^, 94), 252 (100), 234 (32), 180 (42).

*2-[(5-Chloro-4-oxo-4H-1,2,6-thiadiazin-3-yl)amino]-N-methylbenzamide (**8**).* Similar treatment of 3,5-dichloro-4*H*-1,2,6-thiadiazin-4-one (**6**) (183 mg, 1.00 mmol) in EtOH (1 mL), with 2-amino-*N*-methyl-benzamide (150 mg, 1.00 mmol) and 2,6-lutidine (116 μL, 2.00 mmol) after 48 h gave the title compound **8** (228 mg, 77%) as yellow needles, m.p. 252–255 °C (from benzene); *R*_f_ 0.22 (*n*-hexane/*t-*BuOMe, 50:50); (found: C, 44.45; H, 2.92; N, 18.74. C_11_H_9_ClN_4_O_2_S requires C, 44.53; H, 3.06; N, 18.88%); *λ*_max_(DCM)/nm 240 inf (log ε 4.47), 300 (4.64), 336 (4.82), 402 (4.25); *υ*_max_/ cm^−1^ 3310 m, 3111 w, 1626 m, 1595 m, 1585 m, 1541 s, 1537 s, 1452 m, 1435 m, 1406 m, 1329 m, 1308 m, 1285 w, 1238 w, 1178 m, 1169 m, 1150 w, 1107 w, 1053 w, 1001 w, 947 w, 885 m, 858 m, 841 m, 773 m, 752 m, 727 m; *δ*_H_(500 MHz; CDCl_3_) 12.36 (1H, br s, N*H*), 8.76 (1H, s, N*H*), 8.47 (1H, d, *J* 8.2, Ar *H*), 7.78 (1H, d, *J* 7.4, Ar *H*), 7.55 (1H, dd, *J* 7.4, 7.4, Ar *H*), 7.20 (1H, dd, *J* 7.2, 7.2, Ar *H*), 2.81 (3H, d, *J* 3.5, C*H*_3_); *δ*_C_(125 MHz; CDCl_3_) 168.3 (s), 157.3 (s), 149.5 (s), 141.5 (s), 137.9 (s), 131.8 (d), 128.2 (d), 122.9 (d), 121.3 (s), 119.1 (d), 26.2 (q); *m*/*z* (MALDI-TOF) 298 (M^+^ + 2, 25%), 296 (M^+^, 100%), 265 (42).

*3-{[4-(1H-Imidazol-2-yl)phenyl]amino}-5-chloro-4H-1,2,6-thiadiazin-4-one (**21**).* Similar treatment of 3,5-dichloro-4*H*-1,2,6-thiadiazin-4-one (**6**) (91.5 mg, 0.500 mmol) in MeCN (2 mL), with 4-(1*H*-imidazol-2-yl)aniline dihydrochloride (116 mg, 0.500 mmol) and Hünig’s base (261 μL, 1.50 mmol) after 2 h gave the title compound **21** (63.3 mg, 42%) as orange needles, m.p. 298-300 °C (from EtOH/THF); *R*_f_ 0.45 (*t-*BuOMe); *λ*_max_(DCM)/nm 268 (log *ε* 4.00), 342 (4.53), 403 (3.73); *υ*_max_/cm^−1^ 3293 m, 2768 br, 1630 s, 1589s, 1547 s, 1537 s, 1512 s, 1445 m, 1402 w, 1331 w, 1296 w, 1248 w, 1182 m, 1107 m, 1005 w, 949 m, 885m, 868 m, 849 s, 779 m, 733 s, 717 s; *δ*_H_(500 MHz; CDCl_3_) 12.51 (1H, br s, N*H*), 10.21 (1H, s, N*H*), 7.90 (2H, d, *J* 8.9, Ar *H*), 7.87 (2H, d, *J* 8.4, Ar *H*), 7.12 (2H, s, Ar *H*); *δ*_C_(125 MHz; CDCl_3_) 157.1 (s), 149.9 (s), 145.2 (s), 145.2 (s), 137.6 (s), 126.8 (s), 126.5 (s), 126.3 (d), 125.0 (d), 120.6 (d), 113.5 (d); *m*/*z* (ESI+) 306 (MH^+^, 15%), 160 (33), 153 (19), 130 (38), 62 (100).

#### 4.5.4. Preparation of 3,5-Diaminosubstituted Thiadiazines

*3-({5-[(3-Hydroxy-4-methylphenyl)amino]-4-oxo-4H-1,2,6-thiadiazin-3-yl}amino)benzamide (**1**) (General procedure).* To a mixture of 3-chloro-5-[(3-hydroxy-4-methylphenyl)amino]-4*H*-1,2,6-thiadiazin-4-one (**7**) (53.9 mg, 0.200 mmol), Pd[3,5-(F_3_C)_2_C_6_H_3_]_3_ (5.3 mg, 1.25 mol %), DPEPhos (5.3 mg, 5 mol %), powdered dry K_2_CO_3_ (66.4 mg, 0.480 mmol) and 3-aminobenzamide (30.0 mg, 0.220 mmol) was added dioxane (5 mL). The stirred suspension was then deaerated by bubbling of Ar through it for 5 min and then heated at reflux under Ar until complete consumption of the starting thiadiazine (TLC, 2 h). The mixture was cooled to *ca*. 20 °C, then adsorbed onto silica and chromatographed (*n*-hexane/acetone, 50:50) to give the title compound **1** (63.1 mg, 85%) as orange needles, m.p. 297–298 °C (from THF); *R*_f_ 0.30 (*n*-hexane/acetone, 50:50); (found: C, 55.11; H, 4.15; N, 18.83. C_17_H_15_N_5_O_3_S requires C, 55.28; H, 4.09; N, 18.96%); *λ*_max_(EtOH)/nm 207 (log ε 4.62), 338 (4.), 453 (3.80); *υ*_max_/cm^−1^ 3447 w, 3373 w, 3345 m, 3329 m, 3177 w, 1641 m, 1628 m, 1614 m, 1582 s, 1537 s, 1510 s, 1485 m, 1477 m, 1435 m, 1422 m, 1341 w, 1327 w, 1310 w, 1294 w, 1275 w, 1234 m, 1196 w, 1180 m, 1124 m, 1070 w, 999 m, 869 m, 860 m, 787 m; *δ*_H_(500 MHz; DMSO-*d*_6_) 9.63 (1H, s, N*H*), 9.38 (1H, s, N*H*), 9.29 (1H, s, O*H*), 8.30 (1H, s, N*H*), 7.92 (1H, s, N*H*), 7.89 (1H, dd, *J* 8.1, 1.8, Ar *H*), 7.53 (1H, d, *J* 7.8, Ar *H*), 7.41–7.36 (3H, m, Ar *H* and N*H*), 7.05 (1H, dd, *J* 8.1, 1.9, Ar *H*), 6.97 (1H, d, *J* 8.2, Ar *H*), 2.07 (3H, s, C*H*_3_); *δ*_C_(125 MHz; DMSO-*d*_6_) 167.8 (s), 155.1 (s), 154.6 (s), 147.1 (s), 146.8 (s), 139.0 (s), 137.3 (s), 134.9 (s), 130.1 (d), 128.3 (d), 122.2 (d), 121.4 (d), 118.8 (d), 118.4 (s), 110.6 (d), 106.1 (d), 15.4 (q); *m*/*z* (ESI+) 370 (MH^+^, 100%), 369 (M^+^, 7), 214 (14); HRMS found for MH^+^ 370.09675, C_17_H_16_N_5_O_3_S requires 370.09684.

*4-({5-[(3-Hydroxy-4-methylphenyl)amino]-4-oxo-4H-1,2,6-thiadiazin-3-yl}amino)benzamide (**2**).* Similar treatment of 3-chloro-5-[(3-hydroxy-4-methylphenyl)amino]-4*H*-1,2,6-thiadiazin-4-one (**7**) (53.9 mg, 0.200 mmol) with 4-aminobenzamide (30.0 mg, 0.220 mmol) after 1 h gave after chromatography (*n*-hexane/acetone, 50:50) the title compound **2** (69.2 mg, 94%) as orange needles, m.p. > 300 °C (from MeOH/THF); *R*_f_ 0.27 (*n*-hexane/acetone, 50:50); (found: C, 55.39; H, 4.25; N, 18.78. C_17_H_15_N_5_O_3_S requires C, 55.28; H, 4.09; N, 18.96%); *λ*_max_(EtOH)/nm 208 (log *ε* 4.60), 235 inf (4.28), 347 (4.82), 451 (3.94); *υ*_max_/cm^−1^ 3645 w, 3362 m, 3325 m, 3188 w, 2955 m, 2918 w, 2870 w, 1667 m, 1607 m, 1593 m, 1531 m, 1510 s, 1485 m, 1429 m, 1416 m, 1402 m, 1337 m, 1323 m, 1242 m, 1190 m, 1177 m, 1159 m, 1126 m, 1055 m, 1001 w, 955 w, 924 w, 889 m, 860 m, 849 m, 802 m, 785 m; *δ*_H_(500 MHz; DMSO-*d*_6_) 9.76 (1H, s, N*H*), 9.39 (1H, s, N*H*), 9.28 (1H, s, O*H*), 7.88–7.84 (5H, m, Ar *H* and N*H*), 7.40 (1H, d, *J* 2.0, Ar *H*), 7.22 (1H, br s, N*H*), 7.05 (1H, dd, *J* 8.2, 2.1, Ar *H*), 6.98 (1H, d, *J* 8.2, Ar *H*), 2.08 (3H, s, C*H*_3_); *δ*_C_(125 MHz; DMSO-*d*_6_) 167.3 (s), 155.1 (s), 154.8 (s), 147.3 (s), 146.5 (s), 141.8 (s), 137.2 (s), 130.1 (d), 128.1 (d), 127.8 (s), 118.5 (s), 118.1 (d), 110.7 (d), 106.2 (d), 15.5 (q); *m*/*z* (ESI+) 370 (MH^+^, 100%); HRMS found for MH^+^ 370.09655, C_17_H_16_N_5_O_3_S requires 370.09684.

*3-[(2,2-Dioxido-1,3-dihydrobenzo[*c*]thiophen-5-yl)amino]-5-[(3-hydroxy-4-methylphenyl)amino]-4H-1,2,6-thiadiazin-4-one (**3**).* Similar treatment of 3-chloro-5-[(3-hydroxy-4-methylphenyl)amino]-4*H*-1,2,6-thiadiazin-4-one (**7**) (53.9 mg, 0.200 mmol) with 5-amino-1,3-dihydrobenzo[*c*]thiophene 2,2-dioxide (40.3 mg, 0.220 mmol) after 4 h gave after chromatography (*n*-hexane/acetone, 50:50) the title compound **3** (56.9 mg, 68%) as orange needles, m.p. > 300 °C (from EtOH/THF); *R*_f_ 0.74 (*n*-hexane/acetone, 50:50); (found: C, 52.06; H, 3.92; N, 13.37. C_18_H_16_N_4_O_4_S_2_ requires C, 51.91; H, 3.87; N, 13.45%); *λ*_max_(EtOH)/nm 208 (log *ε* 4.62), 338 (4.73), 454 (3.89); *υ*_max_/cm^−1^ 3335 w, 3316 w, 2970 w, 2949 w, 2924 w, 2870 w, 1614 m, 1587 m, 1530 m, 1504 s, 1487 m, 1460 m, 1454 m, 1433 m, 1381 m, 1300 m, 1263 m, 1217 m, 1186 m, 1177 m, 1165 m, 1123 m, 1105 m, 1091 m, 1047 w, 1001 w, 980 w, 910 w, 806 m, 731 w; *δ*_H_(500 MHz; DMSO-*d*_6_) 9.69 (1H, s, N*H*), 9.36 (1H, s, N*H*), 9.27 (1H, s, O*H*), 7.89 (1H, s, Ar *H*), 7.72 (1H, d, *J* 8.2, Ar *H*), 7.39 (1H, s, Ar *H*), 7.32 (1H, d, *J* 8.4, Ar *H*), 7.04 (1H, d, *J* 8.2, Ar *H*), 6.98 (1H, d, *J* 8.2, Ar *H*), 4.50 (2H, s, C*H*_2_), 4.43 (2H, s, C*H*_2_), 2.07 (3H, s, C*H*_3_); *δ*_C_(125 MHz; DMSO-*d*_6_) 155.1 (s), 154.7 (s), 147.2 (s), 146.7 (s), 138.9 (s), 137.3 (s), 132.5 (s), 130.1 (d), 126.1 (d), 126.0 (s), 119.5 (d), 118.5 (s), 116.1 (d), 110.7 (d), 106.2 (d), 56.3 (t), 55.7 (t), 15.4 (q); *m*/*z* (ESI+) 417 (MH^+^, 21%), 391 (100), 214 (24); HRMS found for MH^+^ 417.06829, C_18_H_17_N_4_O_4_S_2_ requires 417.06857.

*N-Methyl-2-([5-({3-[(methylsulfonyl)methyl]phenyl}amino)-4-oxo-4H-1,2,6-thiadiazin-3-yl]-amino}-benzamide (**4**).* Similar treatment of 2-[(5-chloro-4-oxo-4*H*-1,2,6-thiadiazin-3-yl)amino]-*N*-methyl-benzamide (**8**) (59.3 mg, 0.200 mmol) with 3-[(methylsulfonyl)methyl]aniline (40.8 mg, 0.220 mmol) after 3 h gave after chromatography (*t-*BuOMe) the title compound **4** (58.6 mg, 66%) as orange needles, m.p. > 300 °C (from DMA); *R*_f_ 0.23 (*t-*BuOMe); (found: C, 50.98; H, 4.25; N, 15.56. C_19_H_19_N_5_O_4_S_2_ requires C, 51.22; H, 4.30; N, 15.72%); *λ*_max_(THF)/nm 240 (log *ε* 4.54), 267 (4.37), 354 (4.78), 443 (4.19); *υ*_max_/cm^−1^ 3347 w, 3287 w, 2936 w, 1628 m, 1614 m, 1605 m, 1593 m, 1582 m, 1530 s, 1518 s, 1493 m, 1489 m, 1450 m, 1429 m, 1410 m, 1333 m, 1302 m, 1292 m, 1287 m, 1242 m, 1227 w, 1169 m, 1148 w, 1117 m, 1088 w, 972 m, 945 w, 851 w, 789 m, 777 m, 750 m, 729 w; *δ*_H_(300 MHz; DMSO-*d*_6_) 12.09 (1H, s, N*H*), 9.72 (1H, s, N*H*), 8.68 (1H, d, *J* 4.4, Ar *H*), 8.52 (1H, d, *J* 8.3, Ar *H*), 7.89 (1H, s, Ar *H*), 7.75 (2H, dd, *J* 7.4, 7.4, Ar *H*), 7.52 (1H, dd, *J* 7.7, 7.7, Ar *H*), 7.36 (1H, dd, *J* 7.8, 7.8, Ar *H*), 7.10 (2H, dd, *J* 7.8, 7.8, Ar *H*), 4.47 (2H, s, C*H*_2_), 2.94 (3H, s, C*H*_3_), 2.82 (3H, d, *J* 4.4, C*H*_3_); *δ*_C_(125 MHz; DMSO-*d*_6_) 168.5 (s), 155.1 (s), 147.1 (s), 146.6 (s), 139.0 (s), 131.7 (d), 129.4 (s), 128.6 (d), 128.1 (d), 125.4 (d), 121.6 (d), 121.4 (d), 120.5 (s), 119.5 (d), 118.1 (d), 59.5 (t), 26.1 (q), one C (q) resonance missing; *m*/*z* (ESI+) 446 (MH^+^, 17%), 391 (100); HRMS found for MH^+^ 446.09443, C_19_H_20_N_5_O_4_S_2_ requires 446.09512.

*N-Methyl-2-({5-[(4-morpholinophenyl)amino]-4-oxo-4H-1,2,6-thiadiazin-3-yl}amino)benzamide (**5**).* Similar treatment of 2-[(5-chloro-4-oxo-4*H*-1,2,6-thiadiazin-3-yl)amino]-*N*-methylbenzamide (**8**) (59.3 mg, 0.200 mmol) with 4-morpholinoaniline (39.2 mg, 0.220 mmol) after 3 h gave after chromatography (*n*-hexane/acetone, 50:50) the title compound **5** (60.9 mg, 69%) as red plates, m.p. 285–287 °C (from EtOH/THF); *R*_f_ 0.42 (*n*-hexane/acetone, 50:50); (found: C, 57.25; H, 4.91; N, 19.33. C_21_H_22_N_6_O_3_S requires C, 57.52; H, 5.06; N, 19.17%); *λ*_max_(DCM)/nm 356 (log *ε* 4.61), 457 (3.82); *υ*_max_/cm^−1^ 3329 w, 2963 w, 2916 w, 2851 w, 1614 m, 1593 m, 1585 m, 1526 m, 1511 s, 1504 s, 1449 m, 1435 m, 1412 m, 1402 m, 1317 m, 1287 m, 1267 m, 1240 m, 1167 w, 1123 m, 1088 w, 1070 w, 1053 w, 932 m, 810 m, 748 m; *δ*_H_(500 MHz; DMSO-*d*_6_) 12.04 (1H, s, N*H*), 9.49 (1H, s, N*H*), 8.67 (1H, s, N*H*), 8.50 (1H, d, *J* 8.3, Ar *H*), 7.73 (1H, d, *J* 7.6, Ar *H*), 7.63 (2H, d, *J* 8.8, Ar *H*), 7.51 (1H, dd, *J* 7.6, 7.6, Ar *H*), 7.09 (1H, dd, *J* 7.4, 7.4, Ar *H*), 6.93 (2H, d, *J* 8.8, Ar *H*), 3.74 (4H, dd, *J* 3.9, 3.9, C*H*_2_), 3.06 (4H, dd, *J* 3.6, 3.6, C*H*_2_), 2.81 (3H, d, *J* 4.2, C*H*_3_); *δ*_C_(125 MHz; DMSO-*d*_6_) 168.5 (s), 155.0 (s), 147.3 (s), 147.1 (s), 146.1 (s), 139.1 (s), 131.7 (d), 130.9 (s), 128.1 (d), 121.3 (d), 120.8 (d), 120.4 (s), 117.9 (d), 115.3 (d), 66.0 (t), 48.8 (t), 26.1 (q); *m*/*z* (ESI+) 439 (MH^+^, 100%); HRMS found for MH^+^ 439.15424, C_21_H_23_N_6_O_3_S requires 439.15469.

*2-({5-[(3-Hydroxy-4-methylphenyl)amino]-4-oxo-4H-1,2,6-thiadiazin-3-yl}amino)benzamide (**10**).* Similar treatment of 3-chloro-5-[(3-hydroxy-4-methylphenyl)amino]-4*H*-1,2,6-thiadiazin-4-one (**7**) (53.9 mg, 0.200 mmol) with 2-aminobenzamide (30.0 mg, 0.220 mmol) after 3 h gave after filtration of the reaction mixture and washing with H_2_O (5 mL) and EtOH (5 mL) the title compound **10** (67.6 mg, 91%) as orange needles, m.p. 290 °C (decomp., from EtOH/THF); *R*_f_ 0.44 (DCM/Et_2_O, 90:10); (found: C, 55.42; H, 4.16; N, 18.77. C_17_H_15_N_5_O_3_S requires C, 55.28; H, 4.09; N, 18.96%); *λ*_max_(EtOH)/nm 232 (log *ε* 4.14), 262 inf (3.94), 326 inf (4.42), 352 (4.59), 453 (3.85); *υ*_max_/cm^−1^ 3411 br, 1643 m, 1582 s, 1530 s, 1518 s, 1510 s, 1503 s, 1452 m, 1400 m, 1310 m, 1231 m, 1177 m, 1124 m, 999 w, 833 w, 750 m; *δ*_H_(500 MHz; DMSO-*d*_6_) 12.36 (1H, s, N*H*), 9.45 (1H, s, O*H*), 8.56 (1H, d, *J* 8.4, Ar *H*), 8.21 (1H, br s, N*H*), 7.83 (1H, d, *J* 7.9, Ar *H*), 7.67 (1H, br s, N*H*), 7.51 (1H, dd, *J* 7.7, 7.7, Ar *H*), 7.39 (1H, d, *J* 1.7, Ar *H*), 7.09-7.05 (2H, m, Ar *H*), 6.97 (1H, d, *J* 8.1, Ar *H*), 2.07 (3H, s, C*H*_3_), one N*H* resonance missing; *δ*_C_(125 MHz; DMSO-*d*_6_) 170.6 (s), 155.1 (s), 155.0 (s), 147.2 (s), 146.4 (s), 139.8 (s), 137.3 (s), 132.0 (d), 130.1 (d), 128.8 (d), 121.1 (d), 119.3 (s), 118.5 (s), 117.8 (d), 110.5 (d), 106.1 (d), 15.4 (q); *m*/*z* (ESI+) 370 (MH^+^, 100%), 369 (M^+^, 25); HRMS found for MH^+^ 370.09656, C_17_H_16_N_5_O_3_S requires 370.09684.

*3-{[4-(1H-Imidazol-2-yl)phenyl]amino}-5-[(3-hydroxy-4-methylphenyl)amino]-4H-1,2,6-thiadiazin-4-one* (**11**). Similar treatment of 5-chloro-3-{[4-(1*H*-imidazol-2-yl)phenyl]amino}-4*H*-1,2,6-thiadiazin-4-one (**21**) (61.1 mg, 0.200 mmol) with 5-amino-2-methylphenol (27.1 mg, 0.220 mmol) after 18 h gave after filtration of the reaction mixture and washing with H_2_O (5 mL) and EtOH (5 mL) the title compound **11** (63.0 mg, 80%) as orange plates, m.p. > 300 °C (from EtOH/THF); *R*_f_ 0.62 (DCM/THF, 50:50); (found: C, 58.19; H, 4.31; N, 21.26. C_19_H_16_N_6_O_2_S requires C, 58.15; H, 4.11; N, 21.42%); *λ*_max_(THF)/nm 351 (log ε 4.50), 456 (3.56); *υ*_max_/cm^−1^ 3358 w, 3314 w, 3167 w, 1593 m, 1579 m, 1526 m, 1508 m, 1504 s, 1445 m, 1422 m, 1319 m, 1248 m, 1231 w, 1126 w, 1101 w, 1049 w, 947 w, 926 w, 887 w, 858 w, 829 m, 814 w, 772 w, 760 w, 708 m; *δ*_H_(300 MHz; DMSO-*d*_6_) 12.36 (1H, s, N*H*), 9.65 (1H, s, N*H*), 9.36 (1H, s, N*H*), 9.28 (1H, s, O*H*), 7.87 (4H, s, Ar *H*), 7.40 (1H, d, *J* 1.9, Ar *H*), 7.21 (1H, s, Ar *H*), 7.05 (1H, dd, *J* 8.1, 1.9, Ar *H*), 6.98 (2H, d, *J* 7.7, Ar *H*), 2.08 (3H, s, C*H*_3_); *δ*_C_(75 MHz; DMSO-*d*_6_) 155.1 (s), 154.7 (s), 147.1 (s), 146.8 (s), 145.5 (s), 138.7 (s), 137.3 (s), 130.1 (d), 128.6 (d), 125.3 (s), 125.0 (d), 119.1 (d), 118.4 (s), 117.1 (d), 110.6 (d), 106.1 (d), 15.4 (q); *m*/*z* (ESI+) 393 (MH^+^, 100%); HRMS found for MH^+^ 393.11204, C_19_H_17_N_6_O_2_S requires 393.11282.

*4-({5-[(3-Hydroxy-4-methylphenyl)amino]-4-oxo-4H-1,2,6-thiadiazin-3-yl}amino)benzoic acid (**12**).* Similar treatment of 3-chloro-5-[(3-hydroxy-4-methylphenyl)amino]-4*H*-1,2,6-thiadiazin-4-one (**7**) (53.9 mg, 0.200 mmol) with 4-aminobenzoic acid (30.2 mg, 0.220 mmol) after 30 min gave after filtration of the reaction mixture and washing with H_2_O (5 mL) and EtOH (5 mL) the title compound **12** (26.8 mg, 36%) as orange needles, m.p. > 300 °C (from dioxane); *R*_f_ 0.56 (DCM/Et_2_O, 90:10); (found: C, 54.98; H, 3.90; N, 15.16. C_17_H_14_N_4_O_4_S requires C, 55.13; H, 3.81; N, 15.13%); *λ*_max_(MeOH)/nm 212 (log *ε* 4.35), 235 (4.04), 269 (3.75), 349 (4.55), 447 (3.68); *υ*_max_/cm^−1^ 3362 w, 3327 w, 2924 w, 1591 s, 1547 s, 1530 s, 1414 m, 1479 m, 1421 m, 1391 s, 1385 s, 1315 m, 1248 m, 1229 m, 1206 m, 1179 m, 1152 s, 1128 m, 1111 m, 1001 w, 860 w, 837 w, 822 w, 785 m, 752w , 737 w, 710 w; *δ*_H_(500 MHz; DMSO-*d*_6_) 10.25 (1H, br, CO_2_*H*), 9.46 (1H, s, N*H*), 9.25 (1H, s, O*H*), 7.83 (2H, d, *J* 8.0, Ar *H*), 7.66 (2H, d, *J* 8.1, Ar *H*), 7.16 (1H, s, Ar *H*), 6.95 (2H, s, Ar *H*), 2.08 (3H, s, C*H*_3_), one N*H* resonance missing; *δ*_C_(125 MHz; DMSO-*d*_6_) 168.5 (s), 155.7 (s), 154.5 (s), 146.9 (s), 146.7 (s), 138.9 (s), 137.3 (s), 135.9 (s), 129.9 (d), 129.4 (d), 118.4 (s), 117.9 (d), 110.1 (d), 106.1 (d), 15.6 (q); *m*/*z* (ESI+) 371 (MH^+^, 17%), 370 (M^+^, 100); HRMS found for M^+^ 370.07236, C_17_H_14_N_4_O_4_S requires 370.07358.

*2-({5-[(3-Hydroxy-4-methylphenyl)amino]-4-oxo-4H-1,2,6-thiadiazin-3-yl}amino)benzonitrile (**13**).* Similar treatment of 3-chloro-5-[(3-hydroxy-4-methylphenyl)amino]-4*H*-1,2,6-thiadiazin-4-one (**7**) (53.9 mg, 0.200 mmol) with 2-aminobenzonitrile (26.0 mg, 0.220 mmol) after 4 h gave after chromatography (DCM/Et_2_O, 90:10) the title compound **13** (57.9 mg, 82%) as orange needles, m.p. 229–230 °C (from benzene/MeCN); *R*_f_ 0.67 (DCM/Et_2_O, 90:10); (found: C, 58.09; H, 3.57; N, 19.82. C_17_H_13_N_5_O_2_S requires C, 58.11; H, 3.73; N, 19.93%); *λ*_max_(DCM)/nm 264 (log *ε* 3.90), 332 inf (4.36), 345 (4.53), 443 (3.82); *υ*_max_/cm^−1^ 3383 br, 3341 w, 2218 w (C≡N), 1587 s, 1562 s, 1557 s, 1503 m, 1454 w, 1427 m, 1365 w, 1312 m, 1263 m, 1238 m, 1211 m, 1196 m, 1153 s, 112 3s, 999 m, 972 m, 874 m, 862 m, 853 m, 802 m, 727 m; *δ*_H_(300 MHz; DMSO-*d*_6_) 9.53 (1H, s, N*H*), 9.51 (1H, s, N*H*), 9.28 (1H, s, O*H*), 7.99 (1H, d, *J* 8.3, Ar *H*), 7.84 (1H, dd, *J* 7.8, 1.4, Ar *H*), 7.71 (1H, ddd, *J* 7.6, 7.6, 1.5, Ar *H*), 7.37 (1H, d, *J* 2.0, Ar *H*), 6.98 (1H, d, *J* 8.2, Ar *H*), 2.08 (3H, s, C*H*_3_); *δ*_C_(75 MHz; DMSO-*d*_6_) 155.1 (s), 154.5 (s), 147.5 (s), 146.7 (s), 140.8 (s), 137.1 (s), 134.0 (d), 133.0 (d), 130.1 (d), 124.2 (d), 122.1 (d), 118.7 (s), 116.8 (s), 110.8 (d), 106.4 (d), 104.9 (s), 15.4 (q); *m*/*z* (ESI+) 352 (MH^+^, 100%); HRMS found for MH^+^ 352.08600, C_17_H_14_N_5_O_2_S requires 352.08627.

*3-({5-[(3-Hydroxy-4-methylphenyl)amino]-4-oxo-4H-1,2,6-thiadiazin-3-yl}amino)benzonitrile (**14**).* Similar treatment of 3-chloro-5-[(3-hydroxy-4-methylphenyl)amino]-4*H*-1,2,6-thiadiazin-4-one (**7**) (53.9 mg, 0.200 mmol) with 3-aminobenzonitrile (26.0 mg, 0.220 mmol) after 1 h gave after chromatography (DCM/Et_2_O, 90:10) the title compound **14** (65.8 mg, 94%) as yellow needles, m.p. 258–259 °C (from EtOH/THF); *R*_f_ 0.61 (DCM/Et_2_O, 90:10); (found: C, 58.05; H, 3.71; N, 19.85. C_17_H_13_N_5_O_2_S requires C, 58.11; H, 3.73; N, 19.93%); *λ*_max_(THF)/nm 281 (log ε 4.09), 341 (4.76), 447 (3.09); *υ*_max_/cm^−1^ 3345 w, 2237 w (C≡N), 1578 m, 1574 m, 1535 s, 1510 s, 1505 s, 1487 m, 1476 m, 1325 m, 1312 m, 1296 m, 1244 m, 1165 m, 1123 m, 999 m, 858 m, 789 m; *δ*_H_(300 MHz; DMSO-*d*_6_) 9.90 (1H, s, N*H*), 9.40 (1H, s, N*H*), 9.28 (1H, s, O*H*), 8.25 (1H, s, Ar *H*), 8.13 (1H, d, *J* 8.1, Ar *H*), 7.53 (1H, dd, *J* 7.8, 7.8, Ar *H*), 7.46 (1H, d, *J* 7.6, Ar *H*), 7.39 (1H, d, *J* 1.9, Ar *H*), 7.05 (1H, dd, *J* 8.2, 1.9, Ar *H*), 6.98 (1H, d, *J* 8.3, Ar *H*), 2.08 (3H, s, C*H*_3_); *δ*_C_(75 MHz; DMSO-*d*_6_) 155.1 (s), 154.7 (s), 147.4 (s), 146.4 (s), 140.0 (s), 137.1 (s), 130.1 (d), 129.9 (d), 125.7 (d), 123.8 (d), 121.6 (d), 118.8 (s), 118.6 (s), 111.3 (s), 110.7 (d), 106.3 (d), 15.4 (q); *m*/*z* (ESI+) 352 (MH^+^, 100%), 351 (M^+^, 27); HRMS found for MH^+^ 352.08583, C_17_H_14_N_5_O_2_S requires 352.08627.

*4-({5-[(3-Hydroxy-4-methylphenyl)amino]-4-oxo-4H-1,2,6-thiadiazin-3-yl}amino)benzonitrile (**15**).* Similar treatment of 3-chloro-5-[(3-hydroxy-4-methylphenyl)amino]-4*H*-1,2,6-thiadiazin-4-one (**7**) (53.9 mg, 0.200 mmol) with 4-aminobenzonitrile (26.0 mg, 0.220 mmol) after 1 h gave after chromatography (DCM/Et_2_O, 90:10) the title compound **15** (61.5 mg, 88%) as yellow needles, m.p. 280 °C (decomp., from EtOH/THF); *R*_f_ 0.61 (DCM/Et_2_O, 90:10); (found: C, 58.00; H, 3.62; N, 19.85. C_17_H_13_N_5_O_2_S requires C, 58.11; H, 3.73; N, 19.93%); *λ*_max_(THF)/nm 355 (log ε 4.46), 445 (3.65); *υ*_max_/cm^−1^ 3366 w, 3323 w, 2974 w, 2220 w (C≡N), 1620 w, 1614 w, 1601 m, 1580 m, 1535 m, 1531 m, 1518 s, 1510 s, 1487 m, 1414 m, 1325 m, 1248 w, 1231 w, 1177 m, 1123 m, 1092 w, 1049 m, 999 m, 964 w, 910 w, 853 w, 837 m, 797 m, 729 w; *δ*_H_(500 MHz; DMSO-*d*_6_) 10.02 (1H, s, N*H*), 9.44 (1H, s, N*H*), 9.30 (1H, s, O*H*), 8.02 (1H, d, *J* 8.9, Ar *H*), 7.76 (1H, d, *J* 8.9, Ar *H*), 7.38 (1H, d, *J* 2.1, Ar *H*), 7.05 (1H, dd, *J* 8.1, 2.1, Ar *H*), 6.98 (1H, d, *J* 8.3, Ar *H*), 2.08 (3H, s, C*H*_3_); *δ*_C_(125 MHz; DMSO-*d*_6_) 155.1 (s), 154.9 (s), 147.8 (s), 146.1 (s), 143.5 (s), 137.1 (s), 132.9 (d), 130.2 (d), 119.3 (s), 118.77 (d), 118.72 (s), 110.8 (d), 106.4 (d), 103.5 (s), 15.5 (q); *m*/*z* (ESI+) 352 (MH^+^, 100%), 351 (M^+^, 56); HRMS found for MH^+^ 352.08593, C_17_H_14_N_5_O_2_S requires 352.08627.

*4-({5-[(3-Hydroxy-4-methylphenyl)amino]-4-oxo-4H-1,2,6-thiadiazin-3-yl}(oxazol-4-ylmethyl)amino)-benzamide (**16**).* Similar treatment of 3-chloro-5-[(3-hydroxy-4-methylphenyl)amino]-4*H*-1,2,6-thiadiazin-4-one (**7**) (53.9 mg, 0.200 mmol) with 4-[(oxazol-4-ylmethyl)amino]benzamide (**20**) (47.3 mg, 0.220 mmol) after 3 h gave after chromatography (DCM/*t*-BuOMe, 50:50) the title compound **16** (66.9 mg, 74%) as yellow needles, m.p. 235–237 °C (from EtOH/THF); *R*_f_ 0.43 (DCM/*t*-BuOMe, 90:10); (found: C, 56.12; H, 3.97; N, 18.59. C_21_H_18_N_6_O_4_S requires C, 55.99; H, 4.03; N, 18.66%); *λ*_max_(THF)/nm 312 inf (log *ε* 4.54), 347 (4.81), 422 (4.01); *υ*_max_/cm^−1^ 3404 w, 3377 w, 3358 w, 3120 w, 1678 m, 1593 s, 1547 m, 1524 m, 1479 s, 1470 s, 1422 m, 1342 m, 1269 m, 1238 m, 1182 m, 1177 m, 1124 m, 1113 m, 1059 m, 1051 m, 997 m, 984 m, 920 m, 848 m, 831 m, 826 m, 761 m, 756 m; *δ*_H_(300 MHz; DMSO-*d*_6_) 10.03 (1H, s, N*H*), 9.69 (1H, s, N*H*), 9.33 (1H, s, O*H*), 8.34 (1H, d, *J* 0.8, N*H*), 8.01 (1H, d, *J* 0.8, N*H*), 7.71 (1H, d, *J* 8.8, Ar *H*), 7.37 (1H, d, *J* 1.9, Ar *H*), 7.06 (1H, dd, *J* 8.2, 2.0, Ar *H*), 7.00 (1H, d, *J* 8.3, Ar *H*), 6.92 (1H, dd, *J* 5.8, 5.8, Ar *H*), 6.72 (1H, d, *J* 8.8, Ar *H*), 4.25 (2H, d, *J* 5.7, C*H*_2_), 2.08 (3H, s, C*H*_3_); *δ*_C_(75 MHz; DMSO-*d*_6_) 163.7 (s), 155.4 (s), 155.1 (s), 152.1 (s), 151.9 (s), 149.9 (s), 145.0 (s), 137.4 (s), 136.6 (s), 136.1 (d), 130.1 (d), 129.4 (d), 119.8 (s), 119.3 (s), 111.3 (d), 106.9 (d), 38.2 (t), 15.5 (q), one C (d) resonance missing; *m*/*z* (ESI+) 451 (MH^+^, 100%); HRMS found for MH^+^ 451.11757, C_21_H_19_N_6_O_4_S requires 451.11830.

*3-[(3-Hydroxy-4-methylphenyl)amino]-5-{[2-(hydroxymethyl)phenyl]amino}-4H-1,2,6-thiadiazin-4-one (**17**).* Similar treatment of 3-chloro-5-[(3-hydroxy-4-methylphenyl)amino]-4*H*-1,2,6-thiadiazin-4-one (**7**) (53.9 mg, 0.200 mol) with (2-aminophenyl)methanol (27.1 mg, 0.220 mol) after 7 h gave after filtration of the reaction mixture and washing with H_2_O (5 mL) and EtOH (5 mL) the title compound **17** (46.1 mg, 65%) as orange needles, m.p. 242–243 °C (from EtOH/THF); *R*_f_ 0.29 (DCM/*t*-BuOMe, 90:10); (found: C, 57.17; H, 4.59; N, 15.82. C_17_H_16_N_4_O_3_S requires C, 57.29; H, 4.53; N, 15.72%); *λ*_max_(THF)/nm 265 (log *ε* 4.03), 323 (4.71), 334 inf (4.62), 441 (3.89); *υ*_max_/cm^−1^ 3453 w, 3370 w, 3319 w, 3312 w, 2907 w, 1601 w, 1589 m, 1568 m, 1535 m, 1530 m, 1526 m, 1497 s, 1460 m, 1454 m, 1422 m, 1416 m, 1310 m, 1252 m, 1231 w, 1213 w, 1202 w, 1186 w, 1175 w, 1132 w, 1003 m, 934 w, 844 m, 806 m, 748s; *δ*_H_(300 MHz; DMSO-*d*_6_) 9.94 (1H, s, N*H*), 9.38 (1H, s, N*H*), 9.28 (1H, s, O*H*), 8.08 (1H, d, *J* 7.8, Ar *H*), 7.39–7.29 (3H, m, Ar *H*), 7.06–6.96 (3H, m, Ar *H*), 5.72 (1H, s, O*H*), 4.60 (2H, s, C*H*_2_), 2.08 (3H, s, C*H*_3_); *δ*_C_(75 MHz; DMSO-*d*_6_) 155.1 (s), 154.7 (s), 146.9 (s), 146.8 (s), 137.6 (s), 137.3 (s), 130.9 (s), 130.1 (d), 128.4 (d), 127.7 (d), 122.7 (d), 119.5 (d), 118.4 (s), 110.5 (d), 106.0 (d), 62.3 (t), 15.4 (q); *m*/*z* (ESI+) 357 (MH^+^, 98%), 356 (M^+^, 100); HRMS found for MH^+^ 357.10121, C_17_H_17_N_4_O_3_S requires 357.10159.

*3-[(3-Hydroxy-4-methylphenyl)amino]-5-{[2-(hydroxymethyl)-3-(trifluoromethyl)phenyl]amino}-4H-1,2,6-thiadiazin-4-one (**18**).* Similar treatment of 3-chloro-5-[(3-hydroxy-4-methylphenyl)amino]-4*H*-1,2,6-thiadiazin-4-one (**7**) (53.9 mg, 0.200 mol) with [2-amino-6-(trifluoromethyl)phenyl]methanol (42.1 mg, 0.220 mol) after 4 h gave after filtration of the reaction mixture and washing with H_2_O (5 mL) and EtOH (5 mL) the title compound **18** (55.5 mg, 65%) as orange needles, m.p. 241–242 °C (from EtOH/*c*-hexane); *R*_f_ 0.60 (DCM/*t*-BuOMe, 80:20); (found: C, 51.23; H, 3.17; N, 13.26. C_18_H_15_F_3_N_4_O_3_S requires C, 50.94; H, 3.56; N, 13.20%); *λ*_max_(THF)/nm 278 (log *ε* 4.31), 284 (4.30), 340 (4.96), 351 inf (4.89), 447 (4.16); *υ*_max_/ cm^−1^ 3537 w, 3366 w, 3171 w, 1582 m, 1541 m, 1537 s, 1508 m, 1504 s, 1483 m, 1443 m, 1323 m, 1306 m, 1287 m, 1273 w, 1175 m, 1134 m, 1123 m, 1107 m, 1092 m, 1013 w, 997 m, 978 w, 851 w, 789 m; *δ*_H_(500 MHz; DMSO-*d*_6_) 10.20 (1H, s, N*H*), 9.43 (1H, s, N*H*), 9.28 (1H, s, O*H*), 8.38 (1H, d, *J* 8.2, Ar *H*), 7.54 (1H, dd, *J* 8.0, 8.0, Ar *H*), 7.43 (1H, d, *J* 7.8, Ar *H*), 7.37 (1H, d, *J* 2.1, Ar *H*), 7.05 (1H, dd, *J* 8.1, 2.1, Ar *H*), 6.98 (1H, d, *J* 8.3, Ar *H*), 6.07 (1H, dd, *J* 4.5, 4.5, Ar *H*), 4.71 (2H, d, *J* 3.9, C*H*_2_), 2.08 (3H, s, C*H*_3_); *δ*_C_(125 MHz; DMSO-*d*_6_) 155.1 (s), 154.7 (s), 147.1 (s), 146.7 (s), 140.2 (s), 137.2 (s), 130.2 (d), 128.5 (s), 128.2 (d), 127.2 (q, ^2^*J*_CF_ 30.1), 124.1 (q, ^1^*J*_CF_ 274.2), 123.8 (d), 119.4 (q, ^3^*J*_CF_ 5.8), 118.6 (s), 110.6 (d), 106.2 (d), 56.9 (t), 15.5 (q); *δ*_F_(282 MHz; DMSO-*d*_6_) -56.5 (s, C*F*_3_); *m*/*z* (ESI+) 425 (MH^+^, 100%), 424 (M^+^, 9); HRMS found for MH^+^ 425.08829, C_18_H_16_F_3_N_4_O_3_S requires 425.08897.

*2-Cyclopentyl-4-{5-[(3-hydroxy-4-methylphenyl)amino]-4-oxo-4H-1,2,6-thiadiazin-3-yl}-benzoic acid (**19**).* A stirred mixture of 3-chloro-5-[(3-hydroxy-4-methylphenyl)amino]-4*H*-1,2,6-thiadiazin-4-one (**7**) (53.9 mg, 0.200 mol), 4-borono-2-cyclopentylbenzoic acid (**24**) (51.5 mg, 0.220 mol), Na_2_CO_3_ (21.2 mg, 0.200 mol) and Pd(Ph_3_P)_4_ (11.6 mg, 0.0100 mol, 5 mol %), in dioxane/H_2_O 5:3 (0.8 mL) was deaerated by bubbling of Ar through it for 5 min and then heated to *ca*. 100 °C under Ar until complete consumption of the starting thiadiazine (TLC, 3 h). The mixture was then cooled to *ca*. 20 °C, diluted with DCM (10 mL) and extracted with saturated Na_2_CO_3_ (2 × 10 mL). The combined aqueous phase was then acidified with 2 M HCl to a pH of 3 and then extracted with DCM (3 × 10 mL), the organic phase dried (Na_2_SO_4_), filtered and evaporated under reduced pressure to give the title compound **19** (72.9 mg, 86%) as yellow plates, m.p. 277–278 °C (from *c*-hexane); *R*_f_ 0.40 (DCM/*t*-BuOMe, 80:20); (found: C, 62.67; H, 4.91; N, 9.85. C_22_H_21_N_3_O_4_S requires C, 62.40; H, 5.00; N, 9.92%); *λ*_max_(THF)/nm 278 (log *ε* 4.02), 358 (4.37), 422 inf (3.92); *υ*_max_/ cm^−1^ 3455 w, 3321 w, 2955 w, 2866 w, 1694 m, 1620 m, 1595 m, 1547 s, 1422 m, 1310 m, 1267 m, 1234 w, 1175 m, 1119 m, 999 w, 941 w, 901 w, 858 w, 804 w, 797 w, 733 w; *δ*_H_(500 MHz; DMSO-*d*_6_) 13.04 (1H, br, COO*H*), 10.00 (1H, s, N*H*), 9.38 (1H, s, O*H*), 8.23 (1H, dd, *J* 1.5, Ar *H*), 7.87 (1H, dd, *J* 8.2, 1.6, Ar *H*), 7.72 (1H, d, *J* 8.2, Ar *H*), 7.41 (1H, d, *J* 2.0, Ar *H*), 7.11 (1H, dd, *J* 8.1, 2.1, Ar *H*), 7.01 (1H, d, *J* 8.2, Ar *H*), 3.81–3.76 (1H, m, C*H*), 2.09 (3H, s, C*H*_3_), 2.08-2.04 (2H, m, C*H*_2_), 1.76–1.82 (2H, m, C*H*_2_), 1.70–1.61 (2H, m, C*H*_2_), 1.60–1.52 (2H, m, C*H*_2_); *δ*_C_(75 MHz; DMSO-*d*_6_) 169.2 (s), 159.6 (s), 155.1 (s), 152.2 (s), 150.4 (s), 145.4 (s), 137.6 (s), 136.4 (s), 132.4 (s), 130.2 (d), 128.8 (d), 126.5 (d), 125.0 (d), 119.8 (s), 111.7 (d), 107.3 (d), 41.0 (d), 34.4 (t), 35.2 (t), 15.5 (q); *m*/*z* (ESI+) 424 (MH^+^, 100%), 423 (M^+^, 4); HRMS found for MH^+^ 424.13167, C_22_H_22_N_3_O_4_S requires 424.13255.

## Figures and Tables

**Table 1:** Activity of dianilinothiadiazinones (**1**–**5**) on a broad range of protein kinases by Differential Scanning Fluorimetry (DSF) assay (see Supporting Information (SI) Table S1).

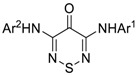				
Entry	Ar^1^	Ar^2^	Number and Kinases Hit DSF > 2 °C *^a^*	
1	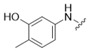	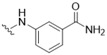	1	TRIB2
2	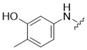	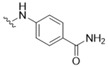	16	CAMK1D/G/K1/K2B, CDC42BPA, CDK2
				CHEK2, DYRK2, MAP2K7, PHKG2
				PIM1, PKMYT1, RPSKA6, STK3, STK17, TTK
3	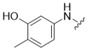	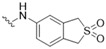	9	CAMK1G/2B, CDC42BPA,
				CLK1, MAP2K7, PIM1,
				STK10, TRIB2, TTK
4	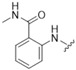	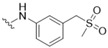	0	
				-

5	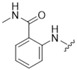	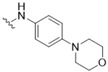	0	
				-


*^a^* Average of 4 experiments.

**Table 2:** CaMKK2 FRET results for advanced TDZ analogues.

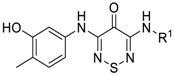					
Compound	R^1^	FRET (IC_50_) *^a^*	Compound	R^1^	FRET (IC_50_) *^a^*
**1**	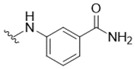	>50	**14**	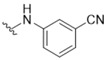	>50
**2**	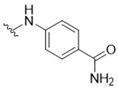	>50	**15**	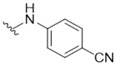	>50
**3**	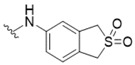	34	**16**	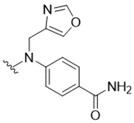	>50
**10**	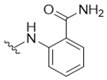	7.8	**17**	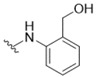	>50
**11**	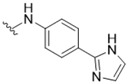	3.2	**18**	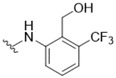	>50
**12**	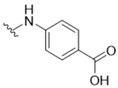	10.5	**19**	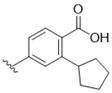	38
**13**	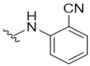	43	**-**	Ponatinib Staurosporine	0.4 & 0.0023

^a^ Average of 2 experiments for CaMKK2/(μM).

**Table 3:** CaMKK2 Enzyme assay results for advanced TDZ analogues.

Compound	FRET (IC_50_) *^a^*	CaMKK2 Enzyme Assay *^b^*
		IC_50_ (μM)
**10**	7.8	11.9
**11**	10.5	6.5
**12**	3.2	4.1
STO-609	-	0.04

*^a^* Average of 2 experiments; *^b^* Data are presented as mean ± SEM for 2 experiments

## Supplementary Materials

The following are available online, Figure S1: Design of TDZs **1**–**5**, Table S1: DSF kinome selectivity panel, Table S2: X-ray crystallography data, Figure S2: Validation of modelling docking poses showing the same hinge contacts as standard 2,4-dianilinopyrimidines, Figure S3 and S4: CaMKK2 FRET results for advanced thiadiazinone analogues, Table S3: CaMKK2 Enzyme assay raw data results for TDZs 10–12 and STO-609, ^1^H and ^13^C-NMR spectra of all new compounds.
