# Supplementary material for: 1,2,6-Thiadiazinones as Novel Narrow Spectrum Calcium/Calmodulin-Dependent Protein Kinase Kinase 2 (CaMKK2) Inhibitors
Source: Molecules. 2018 May 19;23(5):1221. doi: 10.3390/molecules23051221 (PMC6019134; doi:10.3390/molecules23051221)
Supplement: Supplementary file 1 [file molecules-23-01221-s001.zip › molecules-304200-SI.pdf]

Supporting Information

1,2,6-Thiadiazinones as novel narrow spectrum Calcium/calmodulin-dependent protein kinase kinase 2 (CaMKK2) inhibitors

Christopher R. M. Asquith [1,2,*], Paulo H. Godoi [3], Rafael M. Couñago [3], Tuomo Laitinen [4], John W. Scott [5,6,7], Christopher G. Langendorf [5], Jonathan S. Oakhill [5,6], David H. Drewry [1], William J. Zuercher [1,8], Panayiotis A. Koutentis [9], Timothy M. Willson [1], Andreas S. Kalogirou [9,10,*]

[1] Structural Genomics Consortium, UNC Eshelman School of Pharmacy, University of North Carolina at Chapel Hill, Chapel Hill, NC 27599, USA. chris.asquith@unc.edu (C.R.M.A.), david.drewry@unc.edu (D.H.D.), william.zuercher@unc.edu (W.J.Z.), tim.willson@unc.edu (T.M.W.)

[2] Department of Pharmacology, University of North Carolina at Chapel Hill, NC 27599, USA chris.asquith@unc.edu (C.R.M.A)

[5] Structural Genomics Consortium, Universidade Estadual de Campinas - UNICAMP, Campinas, São Paulo, 13083-886, Brazil. phgodoi@yahoo.com (P.H.G.), rafaelcounago@gmail.com (R.M.C.)

[4] School of Pharmacy, Faculty of Health Sciences, University of Eastern Finland, 70211, Kuopio, Finland. tuomo.laitinen@uef.fi (T.L.)

[5] St Vincent's Institute and Department of Medicine, University of Melbourne, 41 Victoria Parade, Fitzroy, 3065, Australia. jscott@svi.edu.au (J.S.), clangendorf@svi.edu.au (C.G.L.), joakhill@svi.edu.au (J.S.O.)

[6] Mary MacKillop Institute for Health Research, Australian Catholic University, 215 Spring Street, Melbourne, 3000, Australia. (J.S.), (J.S.O.)

[7] The Florey Institute of Neuroscience and Mental Health, Parkville, 3052, Australia. (J.S.)

[8] Lineberger Comprehensive Cancer Center, University of North Carolina at Chapel Hill, Chapel Hill, NC 27599, USA. (W.J.Z.)

[9] Department of Chemistry, University of Cyprus, P. O. Box 20537, 1678 Nicosia, Cyprus. koutenti@ucy.ac.cy (P.A.K.), kalogirou.andreas@ucy.ac.cy (A.S.K.)

[10] Department of Life Sciences, School of Sciences, European University Cyprus, 6 Diogenis Str., Engomi, P. O. Box 22006, 1516 Nicosia, Cyprus. A.Kalogirou@external.euc.ac.cy (A.S.K.)

Contents **Page**

S1.	Design of TDZs **1-5**	S3
S2.	DSF Panel data	S4-5
S3.	X-ray crystallography data	S6
S4.	Validation of modelling docking poses	S7
S5.	CaMKK2 FRET results for TDZs **1-5** and **10-19**	S8
S6.	CaMKK2 Enzyme assay raw data results for TDZs **10-12** and STO-609	S9
S7.	References	S9
S8.	^{1}H and ^{13}C NMR spectra of all new compounds	S10-S50

S1. Design of TDZs 1-5

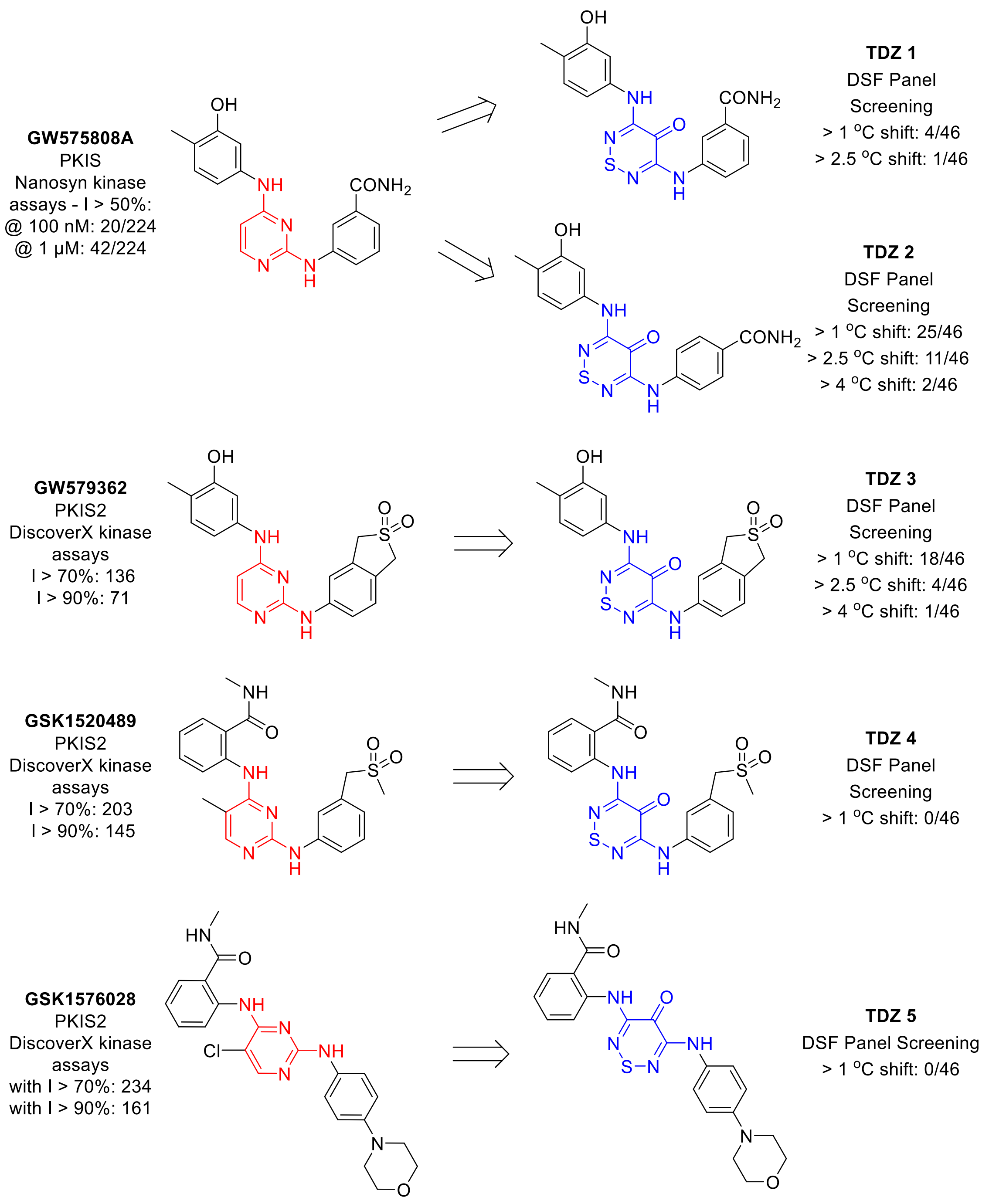

Figure S1. Initial set designed using the template strategy to utilizing the thiadiazinone core modification (blue) over existing 2,4-dianilinopyrimidine (red) kinase inhibitors from PKIS and PKIS2. These compounds were then profiled by DSF on a representative set of 46 kinases.

S2. DSF Panel data

Table S1. DSF kinome selectivity panel.

Number	Kinases	Compounds, ΔTm (°C)				
		**1**	**2**	**3**	**4**	**5**
1	AAK1	0,6	1,5	0,6	-0,4	-0,4
2	BMP2K	-0,4	-0,2	0,1	-0,1	-0,1
3	BMXA	0,1	-0,1	0,0	0,0	0,0
4	BRAFA	-0,1	0,2	0,7	0,1	0,4
5	CAMK1D	0,8	4,3	1,4	-1,3	-0,9
6	CAMK1G	1,8	3,3	3,0	0,2	0,1
7	CAMKK1	0,0	3,7	1,4	-0,4	-0,4
8	CAMKK2B	0,5	2,9	2,3	-0,4	-0,5
9	CDC42BPA	0,3	4,8	2,3	-0,8	-0,6
10	CDK2	0,2	2,7	1,8	0,2	-0,1
11	CDKL1	1,2	1,0	0,9	0,5	0,5
12	CHEK2	0,6	3,1	1,6	0,6	0,8
13	CLK1	0,5	0,9	2,1	0,0	1,0
14	CSNK1G1	-0,6	-1,0	-0,7	0,3	0,0
15	CSNK1G3	0,0	0,1	-0,2	-0,1	0,0
16	CSNK2A1	-0,9	-0,2	-0,6	-0,4	-0,4
17	DYRK1AA	-0,6	0,9	-0,1	-0,5	-0,8
18	DYRK2A	0,1	2,4	0,7	0,1	0,1
19	EPHA2A	0,2	0,5	0,8	-0,1	0,0
20	GAKA	-0,3	1,4	0,0	-0,4	-0,4
21	GSG2A	0,6	1,3	1,2	0,4	0,3
22	MAPK1A	-0,2	1,4	0,1	-0,3	-0,3
23	MAP2K7A	1,1	3,1	3,2	0,6	1,0
24	MAPK14B	0,0	0,6	1,0	-0,2	-0,3
25	MAPK3A	0,1	1,2	0,4	0,1	0,1
26	PHKG2A	0,7	2,4	1,6	0,2	0,2
27	PIM1A	1,0	3,5	4,1	-0,7	-0,2
28	PLK1A	0,3	-0,2	1,1	0,2	0,2
29	PKMYT1A	0,0	3,0	1,2	-0,3	-0,8
30	PRPF4BA	0,7	0,1	0,6	0,1	0,1
31	RPS6KA1A	-0,6	1,3	-0,4	-0,4	-0,4
32	RPS6KA5A	-0,9	1,7	-0,3	-1,2	-1,1
33	RPS6KA6A	0,5	2,6	0,9	-0,3	-0,6
34	SLKA	-0,6	-0,1	-0,2	-0,8	-0,7
35	SRPK1A	0,0	1,8	0,0	0,0	0,0
36	SRPK2A	-0,2	0,6	0,1	-0,3	-0,1
37	STK3	0,2	2,1	1,1	-0,4	-0,3

38	STK6A	-0,3	1,0	0,1	-0,1	0,5
39	STK10A	0,5	0,9	2,2	0,2	-0,4
40	STK17AA	-0,5	2,2	1,7	-0,8	-0,8
41	STK24A	-0,1	-	-0,3	0,0	-1,1
42	STK38LA	0,2	0,4	0,8	0,4	0,3
43	TRIB2A	2,5	-	4,9	1,0	0,8
44	TTKA	0,8	2,6	2,1	0,3	0,1
45	VRK1	-0,4	-0,7	-0,2	-0,3	-0,3
46	VRK2A	-0,6	1,8	0,1	-0,3	-0,3

S3. X-ray crystallography data

Table S2. Data processing and structure refinement statistics of the crystal structure of compound **2** in complex with CAMKK2

Ligand	thiadiazinone benzamide (**2**)
Data collection	
X-ray source	DLS I03
Wavelength (Å)	0.9763
Space group	$P6_1$
Cell dimensions	
a, b, c (Å)	100.5, 100.5, 69.7
α, β, γ (°)	90.0, 90.0, 120.0
Resolution (Å)*	19.61-1.60 (1.63-1.60)
No. of unique reflections*	31,197 (2,111)
Rmerge (%)*	12.4 (51.4)
Mean I/σI *	7.3 (2.4)
Mean CC(1/2)*	1.0 (0.8)
Completeness (%)*	99.7 (99.7)
Redundancy*	5.1 (5.2)
Refinement	
Resolution (Å)	19.96-1.90 (1.94-1.90)
Rcryst / Rfree (%)	16.1 / 18.5
No. of atoms / Mean B-factor (Å)	
Protein atoms	2,096 / 33.3
Solvent atoms	246 / 49.4
Ligand atoms	27 / 28.0
Rmsd bond lengths (Å)	0.010
Rmsd bong angles (degrees)	1.03
Ramachandran statistics (%)	
Favored	98.0
Allowed	2.0
Outlier	0
PDB ID	5VT1
Crystallization conditions	24% PEG 8,000, 0.1M magnesium acetate, 0.1M CHC buffer, pH 7.0

S4. Validation of modelling docking poses

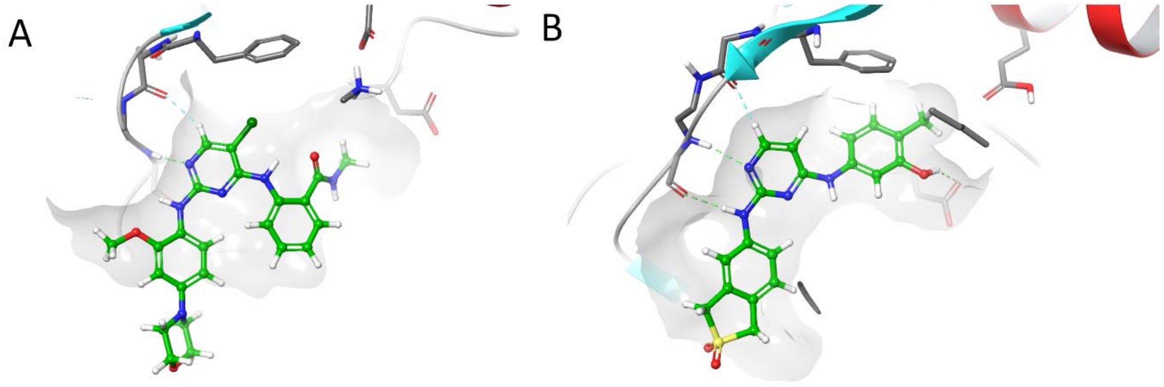

Figure S2. Validation of modelling docking poses showing the same hinge contacts as standard 2,4-dianilinopyrimidines: Pose of GW575808A (A) and GW579362 (B) docked into CaMKK2 (PDB 2Z2V) [1]

S5. CaMKK2 FRET results for TDZs 1-5 and 10-19

Figure S3. FRET results for TDZs for **1-5** on CaMKK2

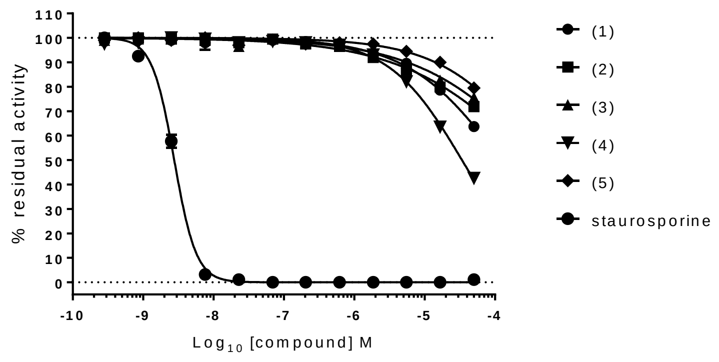

Figure S4. FRET results for TDZs for **10-19** on CaMKK2

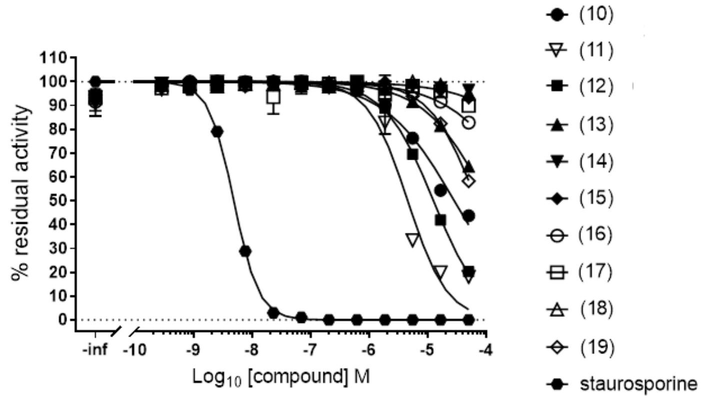

S6. CaMKK2 Enzyme assay raw data results for TDZs 10-12 and STO-609

Table S3. Enzyme assay raw data for TDZs 10-12 and STO-609 including standard error measurements.

[drug] (µM)	**10**	SEM	**11**	SEM	**12**	SEM	**STO-609**	SEM
100	2.19	0.09	1.23	0.09	0.83	0.04	0.07	0.00
10	52.53	1.68	42.11	2.46	28.43	1.13	1.89	0.09
1	85.36	4.15	79.37	3.56	79.70	4.23	4.64	0.31
0.1	91.02	5.65	84.85	5.21	89.54	3.04	42.21	2.36
0.01	98.48	4.62	93.09	2.65	95.03	5.19	69.88	3.00
0.001	102.30	6.19	98.56	6.36	102.38	7.32	88.33	6.02
0	100.00	5.82	100.00	4.71	100.00	4.30	100.00	6.19
IC50 (µM)	11.87	2.82	6.51	2.53	4.09	0.88	0.04	0.02

S7. References

1. Kukimoto-Niino, M.; Yoshikawa, S.; Takagi, T.; Ohsawa, N.; Tomabechi, Y.; Terada, T.; Shirouzu, M.; Suzuki, A.; Lee, S.; Yamauchi, T.; Okada-Iwabu, M.; Iwabu, M.; Kadowaki, T.; Minokoshi, Y.; Yokoyama, S. Crystal structure of the Ca^{2+}/calmodulin-dependent protein kinase kinase in complex with the inhibitor STO-609. *J. Biol. Chem.* **2011**, 286, 22570-22579. DOI: 10.1074/jbc.M111.251710.

S8. ^{1}H and ^{13}C NMR spectra of all new compounds

^{1}H-NMR of 4-((oxazol-4-ylmethyl)amino)benzamide (**20**)

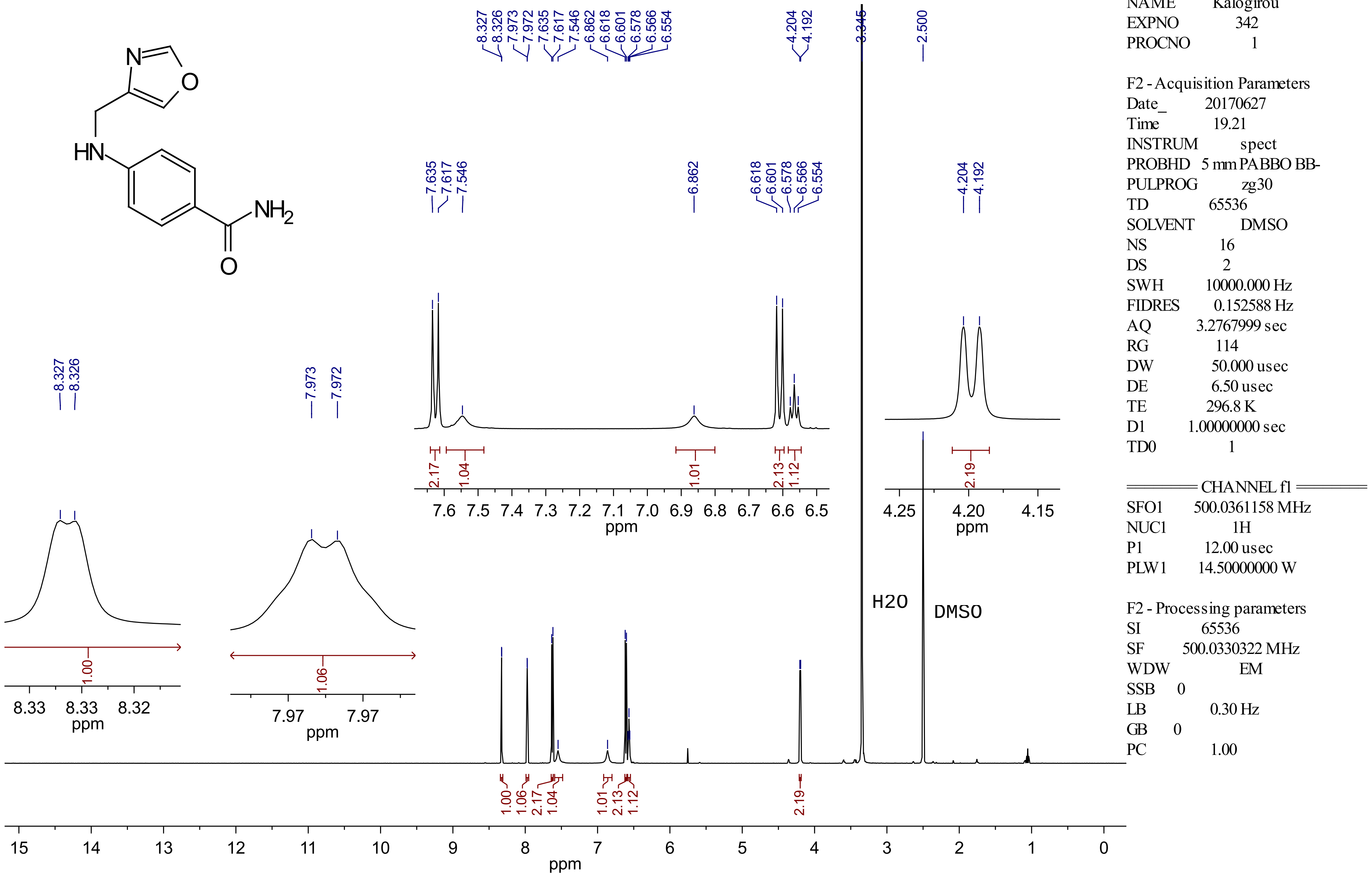

^{13}C-NMR of 4-((oxazol-4-ylmethyl)amino)benzamide (**20**)

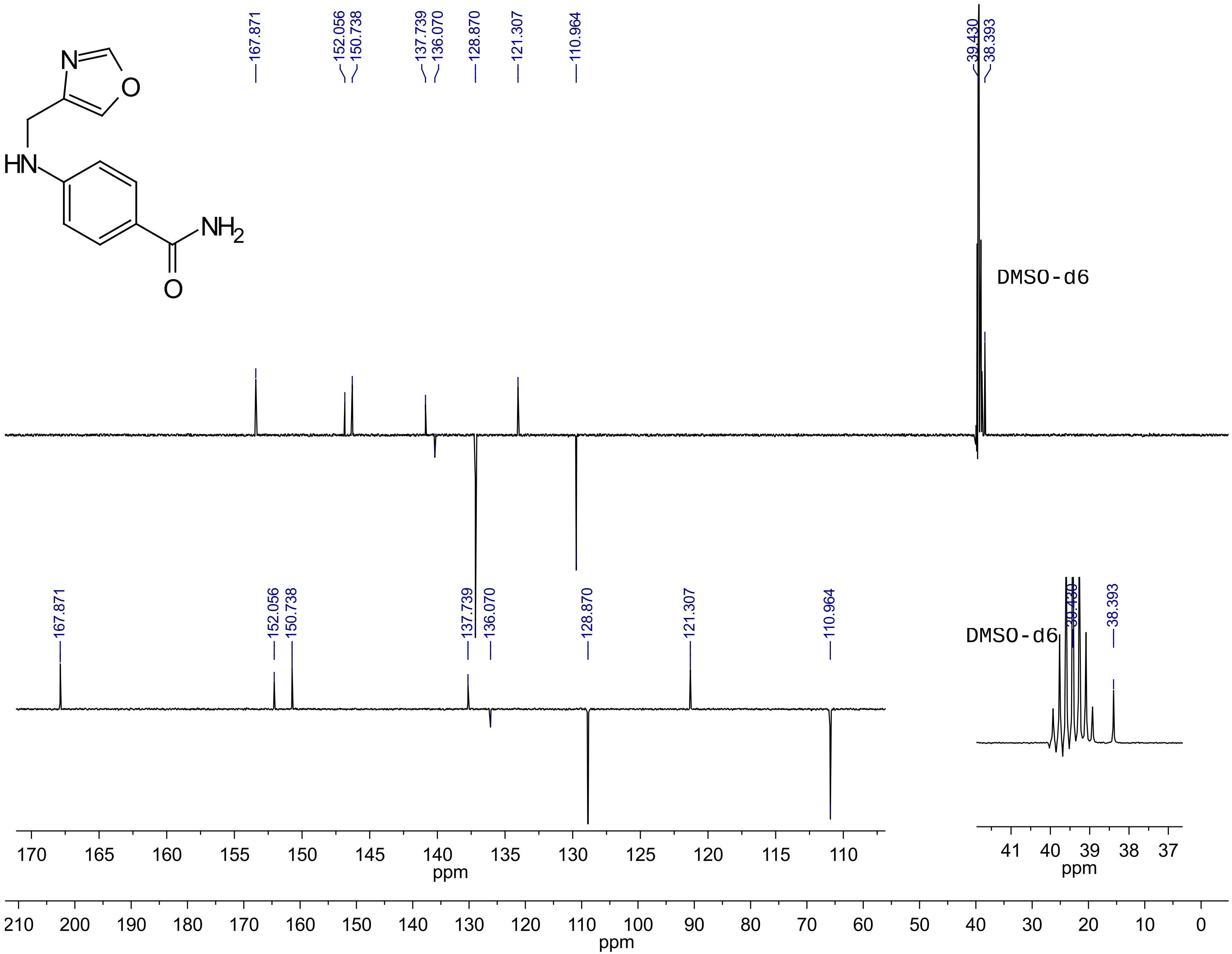

Current Data Parameters
NAME Kalogirou
EXPNO 343
PROCNO 1
F2 - Acquisition Parameters
Date_ 20170627
Time 19.32
INSTRUM spect
PROBHD 5 mm PABBO BB-
PULPROG jmod
TD 65536
SOLVENT DMSO
NS 5120
DS 4
SWH 29761.904 Hz
FIDRES 0.454131 Hz
AQ 1.1010048 sec
RG 2050
DW 16.800 usec
DE 6.50 usec
TE 297.7 K
CNST2 145.0000000
CNST11 1.0000000
D1 2.00000000 sec
D20 0.00689655 sec
TD0 1
======== CHANNEL f1 ========
SFO1 125.7459782 MHz
NUC1 13C
P1 9.00 usec
P2 18.00 usec
PLW1 140.00000000 W
======== CHANNEL f2 ========
SFO2 500.0350280 MHz
NUC2 1H
CPDPRG[2 waltz16
PCPD2 80.00 usec
PLW2 14.50000000 W
PLW12 0.32624999 W
F2 - Processing parameters
SI 32768
SF 125.7334743 MHz
WDW EM
SSB 0
LB 1.00 Hz
GB 0
PC 1.40

^{1}H-NMR of 4-borono-2-cyclopentylbenzoic acid (**22**)

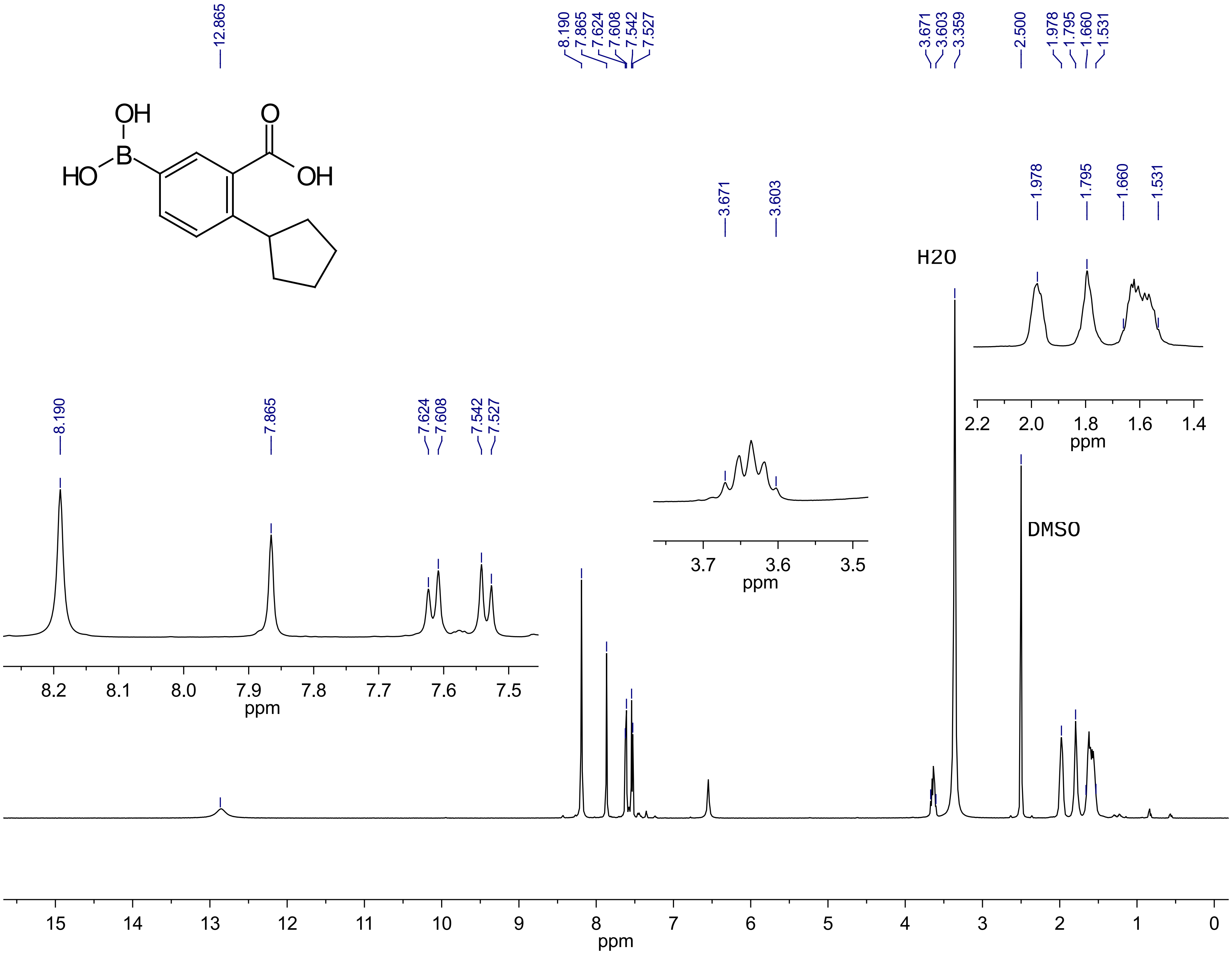

Current Data Parameters	
NAME	Kalogirou
EXPNO	537
PROCNO	1
F2 - Acquisition Parameters	
Date_	20180414
Time	18.04
INSTRUM	spect
PROBHD	5 mm PABBO BB-
PULPROG	zg30
TD	65536
SOLVENT	DMSO
NS	16
DS	2
SWH	10000.000 Hz
FIDRES	0.152588 Hz
AQ	3.2767999 sec
RG	101
DW	50.000 usec
DE	6.50 usec
TE	295.0 K
D1	1.00000000 sec
TD0	1
======== CHANNEL f1 ========	
SFO1	500.0361158 MHz
NUC1	1H
P1	12.00 usec
PLW1	14.80000019 W
F2 - Processing parameters	
SI	65536
SF	500.0330316 MHz
WDW	EM
SSB	0
LB	0.30 Hz
GB	0
PC	1.00

^{13}C-NMR of 4-borono-2-cyclopentylbenzoic acid (**22**)

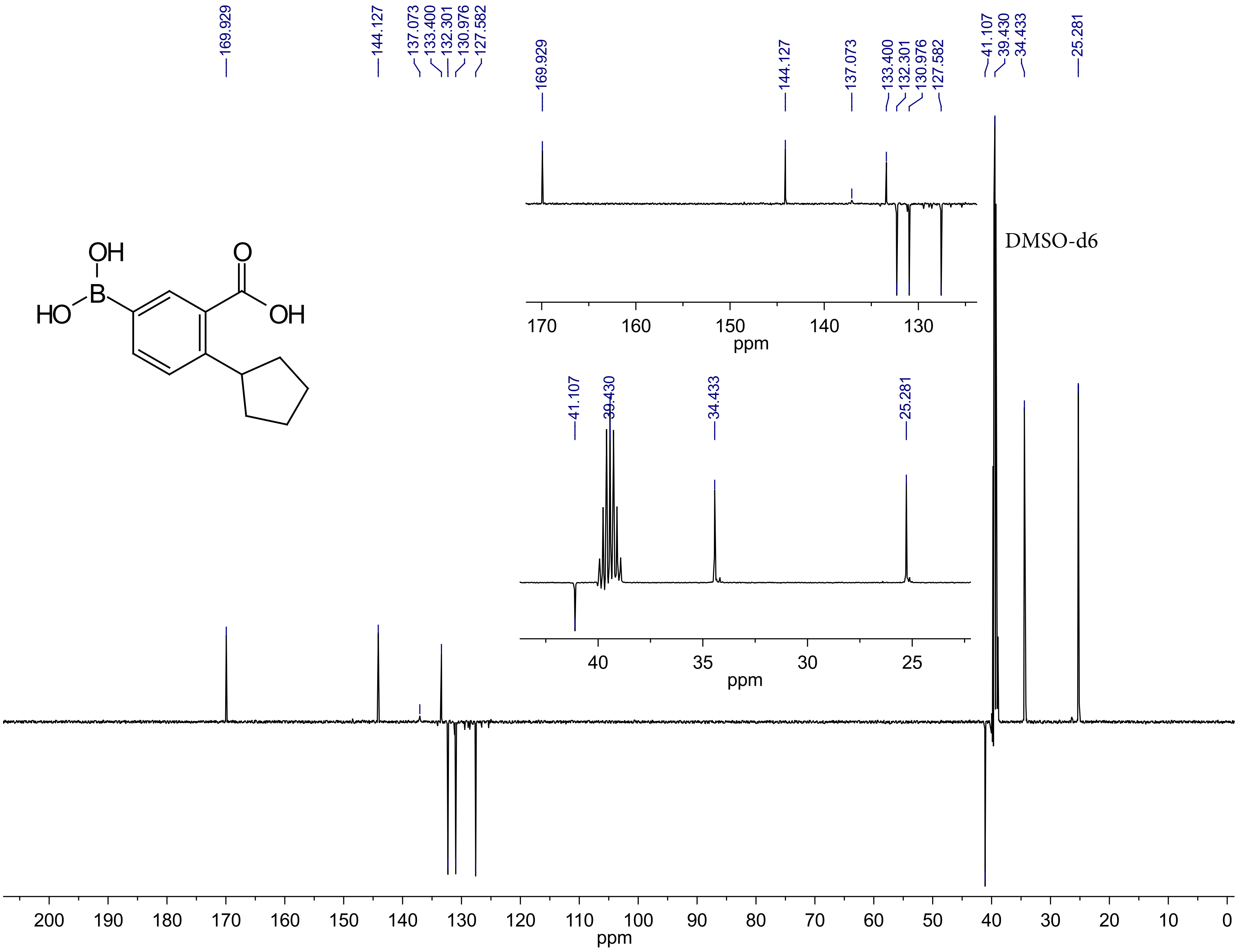

Current Data Parameters	
NAME	Kalogirou
EXPNO	538
PROCNO	1
F2 - Acquisition Parameters	
Date_	20180414
Time	18.17
INSTRUM	spect
PROBHD	5 mm PABBO BB-
PULPROG	jmod
TD	65536
SOLVENT	DMSO
NS	8192
DS	4
SWH	29761.904 Hz
FIDRES	0.454131 Hz
AQ	1.1010048 sec
RG	1820
DW	16.800 usec
DE	6.50 usec
TE	296.1 K
CNST2	145.0000000
CNST11	1.0000000
D1	2.00000000 sec
D20	0.00689655 sec
TD0	1
CHANNEL f1	
SFO1	125.7459782 MHz
NUC1	13C
P1	9.40 usec
P2	18.80 usec
PLW1	140.00000000 W
CHANNEL f2	
SFO2	500.0350280 MHz
NUC2	1H
CPDPRG[2	waltz16
PCPD2	80.00 usec
PLW2	14.80000019 W
PLW12	0.33300000 W
F2 - Processing parameters	
SI	32768
SF	125.7334049 MHz
WDW	EM
SSB	0
LB	1.00 Hz
GB	0
PC	1.40

^{1}H-NMR of 3-chloro-5-((3-hydroxy-4-methylphenyl)amino)-4*H*-1,2,6-thiadiazin-4-one (**7**)

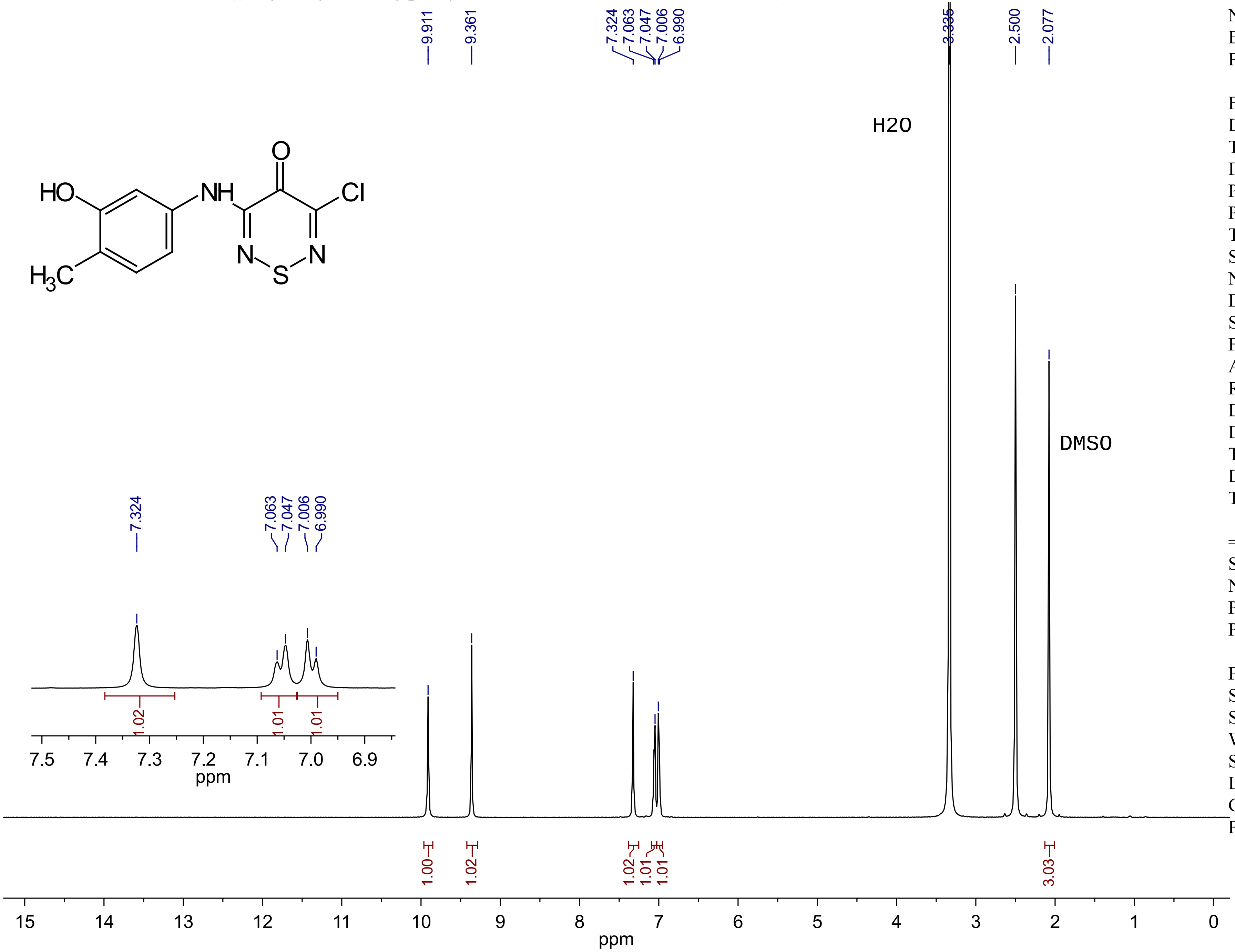

Current Data Parameters
NAME Kalogirou
EXPNO 30
PROCNO 1

F2 - Acquisition Parameters
Date_ 20160510
Time 20.20
INSTRUM spect
PROBHD 5 mm PABBO BB-
PULPROG zg30
TD 65536
SOLVENT DMSO
NS 16
DS 2
SWH 10000.000 Hz
FIDRES 0.152588 Hz
AQ 3.2767999 sec
RG 128
DW 50.000 usec
DE 6.50 usec
TE 297.8 K
D1 1.00000000 sec
TD0 1

CHANNEL f1
SFO1 500.0361158 MHz
NUC1 1H
P1 12.00 usec
PLW1 14.50000000 W

F2 - Processing parameters
SI 65536
SF 500.0330321 MHz
WDW EM
SSB 0
LB 0.30 Hz
GB 0
PC 1.00

^{13}C-NMR of 3-chloro-5-((3-hydroxy-4-methylphenyl)amino)-4*H*-1,2,6-thiadiazin-4-one (**7**)

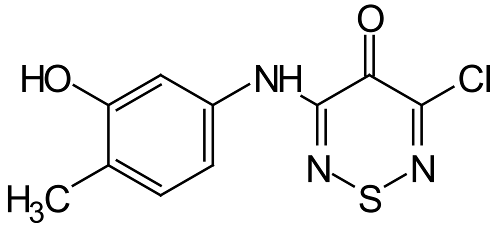

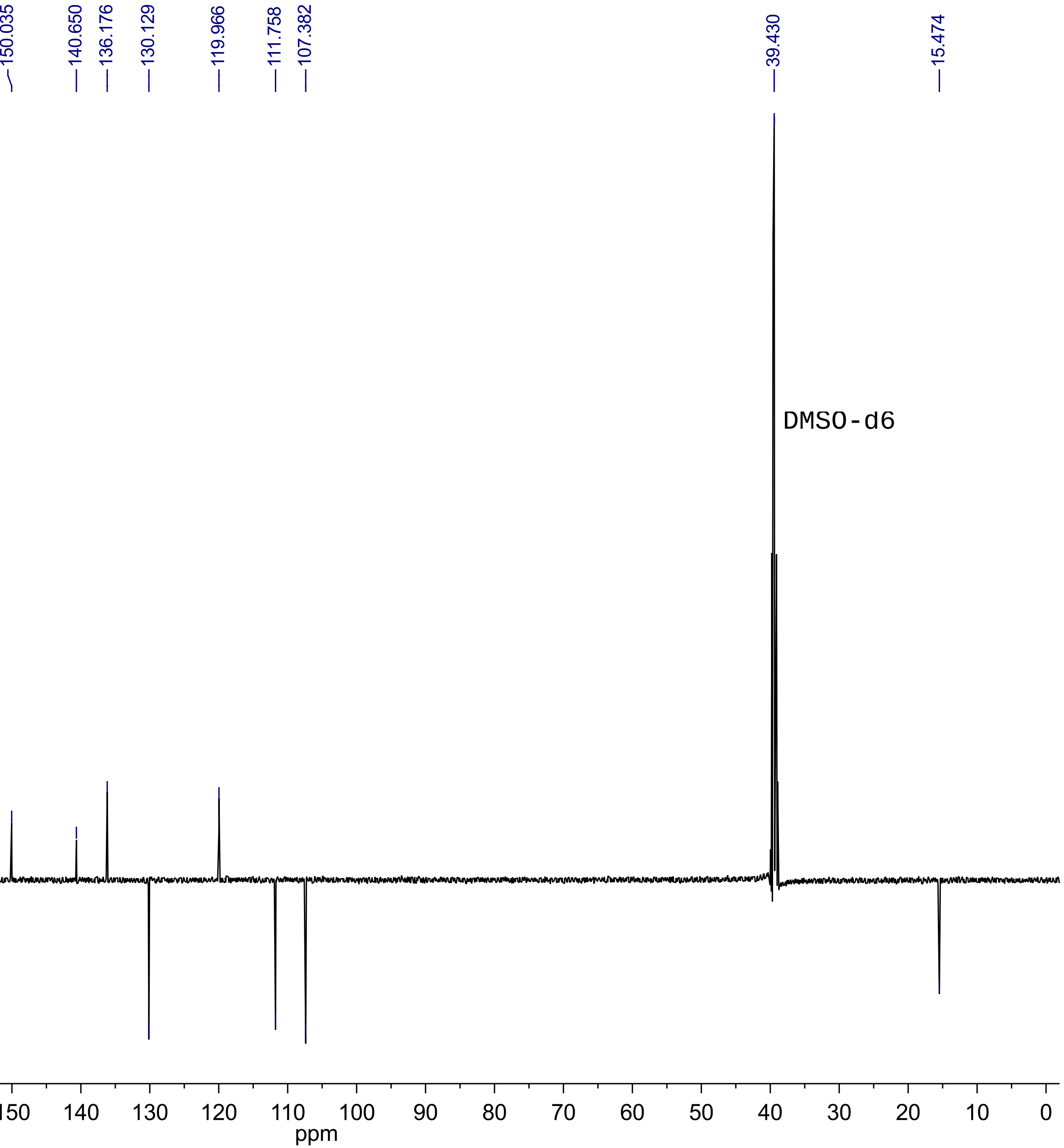

Current Data Parameters
NAME Kalogirou
EXPNO 31
PROCNO 1
F2 - Acquisition Parameters
Date_ 20160510
Time 23.17
INSTRUM spect
PROBHD 5 mm PABBO BB-
PULPROG jmod
TD 65536
SOLVENT DMSO
NS 3328
DS 4
SWH 29761.904 Hz
FIDRES 0.454131 Hz
AQ 1.1010048 sec
RG 2050
DW 16.800 usec
DE 6.50 usec
TE 299.0 K
CNST2 145.0000000
CNST11 1.0000000
D1 2.00000000 sec
D20 0.00689655 sec
TD0 1
======== CHANNEL f1 ========
SFO1 125.7459782 MHz
NUC1 13C
P1 9.00 usec
P2 18.00 usec
PLW1 133.00000000 W
======== CHANNEL f2 ========
SFO2 500.0350280 MHz
NUC2 1H
CPDPRG[2 waltz16
PCPD2 80.00 usec
PLW2 14.50000000 W
PLW12 0.32624999 W
F2 - Processing parameters
SI 32768
SF 125.7334656 MHz
WDW EM
SSB 0
LB 1.00 Hz
GB 0
PC 1.40

^{1}H-NMR of 2-((5-chloro-4-oxo-4*H*-1,2,6-thiadiazin-3-yl)amino)-N-methylbenzamide (**8**)

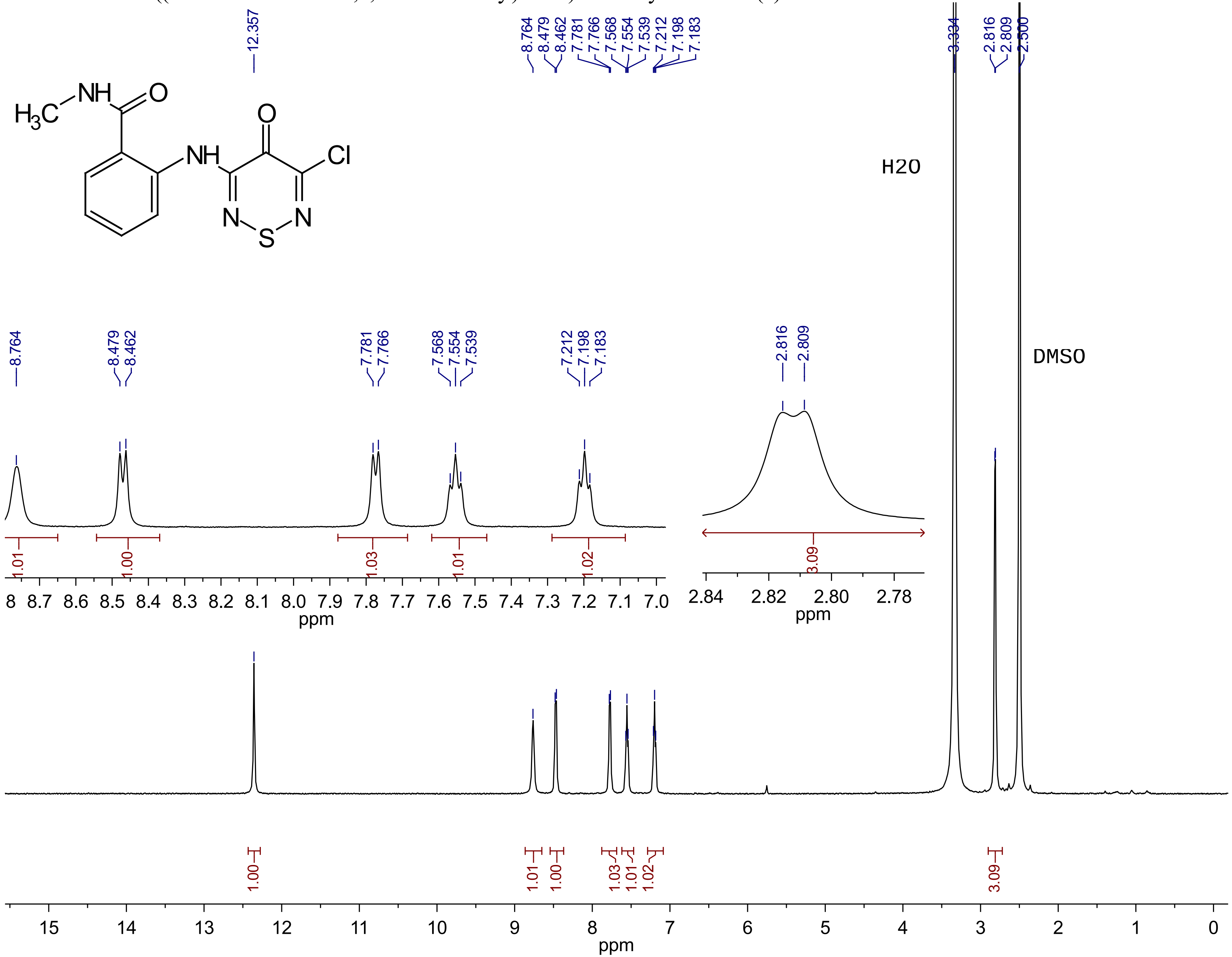

Current Data Parameters
NAME Kalogirou
EXPNO 32
PROCNO 1

F2 - Acquisition Parameters
Date_ 20160511
Time 19.26
INSTRUM spect
PROBHD 5 mm PABBO BB-
PULPROG zg30
TD 65536
SOLVENT DMSO
NS 16
DS 2
SWH 10000.000 Hz
FIDRES 0.152588 Hz
AQ 3.2767999 sec
RG 128
DW 50.000 usec
DE 6.50 usec
TE 297.6 K
D1 1.00000000 sec
TD0 1

======== CHANNEL f1 ========
SFO1 500.0361158 MHz
NUC1 1H
P1 12.00 usec
PLW1 14.50000000 W

F2 - Processing parameters
SI 65536
SF 500.0330322 MHz
WDW EM
SSB 0
LB 0.30 Hz
GB 0
PC 1.00

^{13}C-NMR of 2-((5-chloro-4-oxo-4*H*-1,2,6-thiadiazin-3-yl)amino)-N-methylbenzamide (**8**)

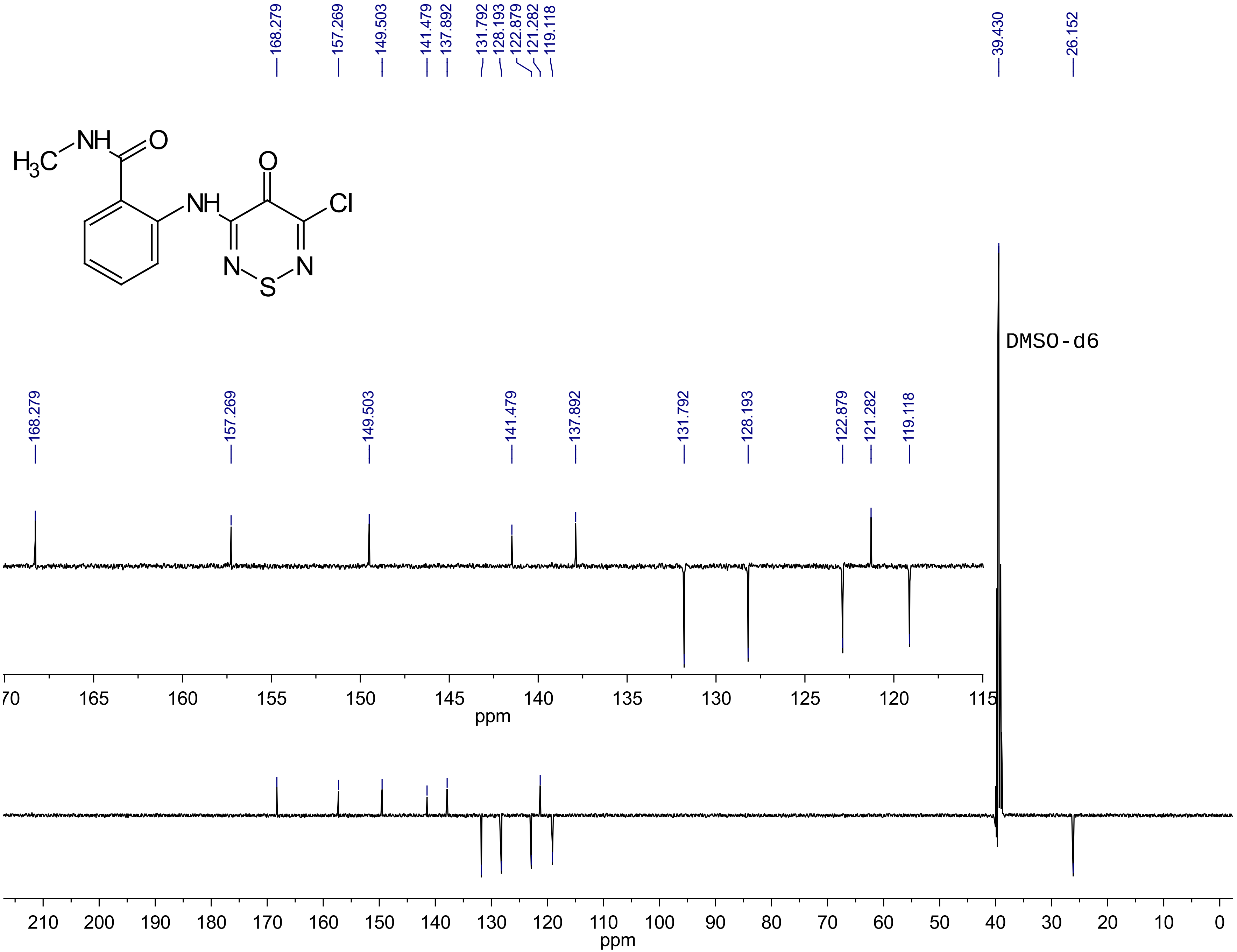

Current Data Parameters
NAME Kalogirou
EXPNO 33
PROCNO 1
F2 - Acquisition Parameters
Date_ 20160511
Time 22.03
INSTRUM spect
PROBHD 5 mm PABBO BB-
PULPROG jmod
TD 65536
SOLVENT DMSO
NS 4608
DS 4
SWH 29761.904 Hz
FIDRES 0.454131 Hz
AQ 1.1010048 sec
RG 2050
DW 16.800 usec
DE 6.50 usec
TE 299.0 K
CNST2 145.0000000
CNST11 1.0000000
D1 2.00000000 sec
D20 0.00689655 sec
TD0 1
======== CHANNEL f1 ========
SFO1 125.7459782 MHz
NUC1 13C
P1 9.00 usec
P2 18.00 usec
PLW1 133.00000000 W
======== CHANNEL f2 ========
SFO2 500.0350280 MHz
NUC2 1H
CPDPRG[2 waltz16
PCPD2 80.00 usec
PLW2 14.50000000 W
PLW12 0.32624999 W
F2 - Processing parameters
SI 32768
SF 125.7334653 MHz
WDW EM
SSB 0
LB 1.00 Hz
GB 0
PC 1.40

^{1}H-NMR of 3-((4-(1H-imidazol-2-yl)phenyl)amino)-5-chloro-4*H*-1,2,6-thiadiazin-4-one (**21**)

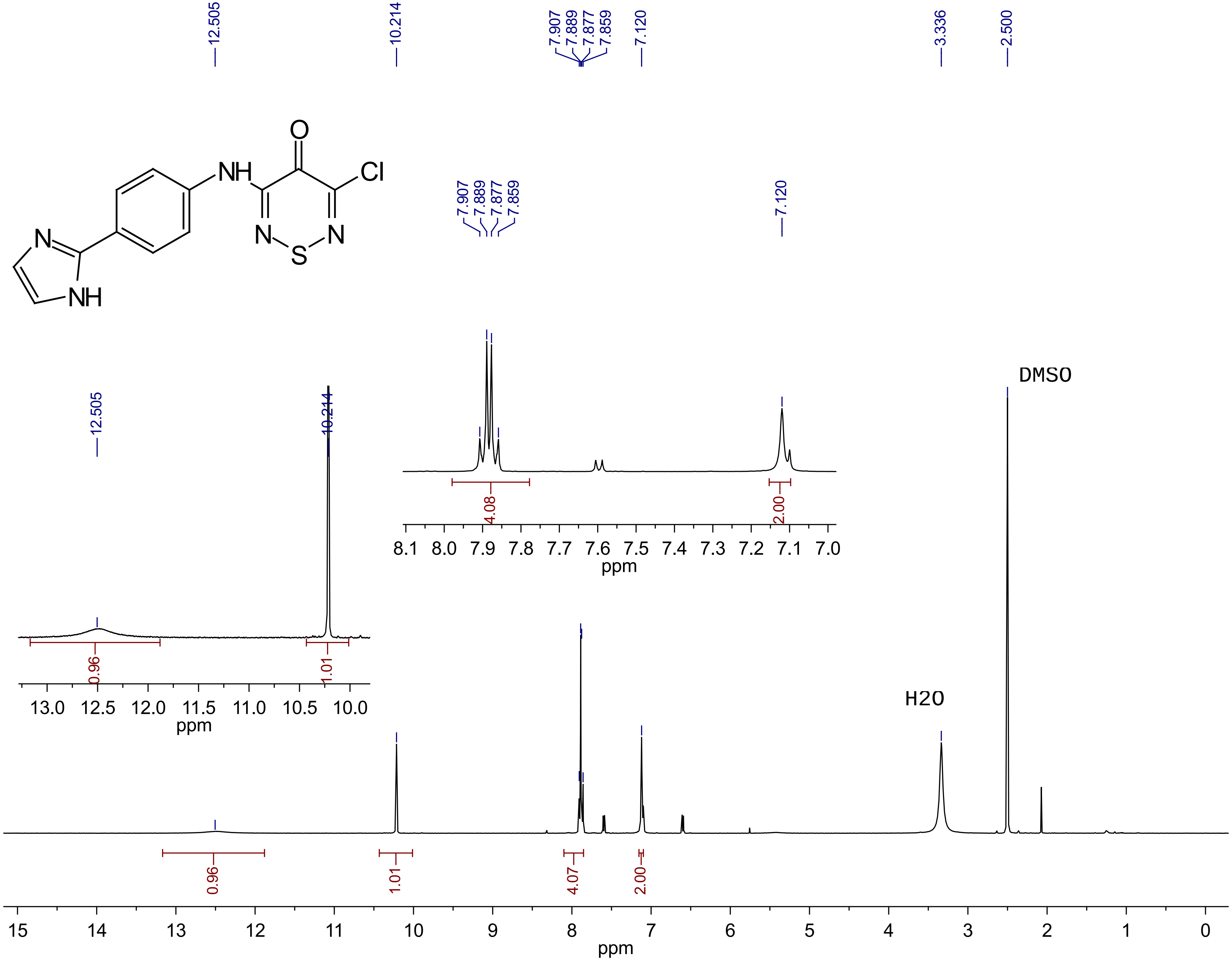

Current Data Parameters
NAME Kalogirou
EXPNO 417
PROCNO 1

F2 - Acquisition Parameters
Date_ 20170923
Time 0.50
INSTRUM spect
PROBHD 5 mm PABBO BB-
PULPROG zg30
TD 65536
SOLVENT DMSO
NS 16
DS 2
SWH 10000.000 Hz
FIDRES 0.152588 Hz
AQ 3.2767999 sec
RG 161
DW 50.000 usec
DE 6.50 usec
TE 296.9 K
D1 1.00000000 sec
TD0 1

======== CHANNEL f1 ========
SFO1 500.0361158 MHz
NUC1 1H
P1 12.00 usec
PLW1 14.50000000 W

F2 - Processing parameters
SI 65536
SF 500.0330319 MHz
WDW EM
SSB 0
LB 0.30 Hz
GB 0
PC 1.00

^{13}C-NMR of 3-((4-(1H-imidazol-2-yl)phenyl)amino)-5-chloro-4*H*-1,2,6-thiadiazin-4-one (**21**)

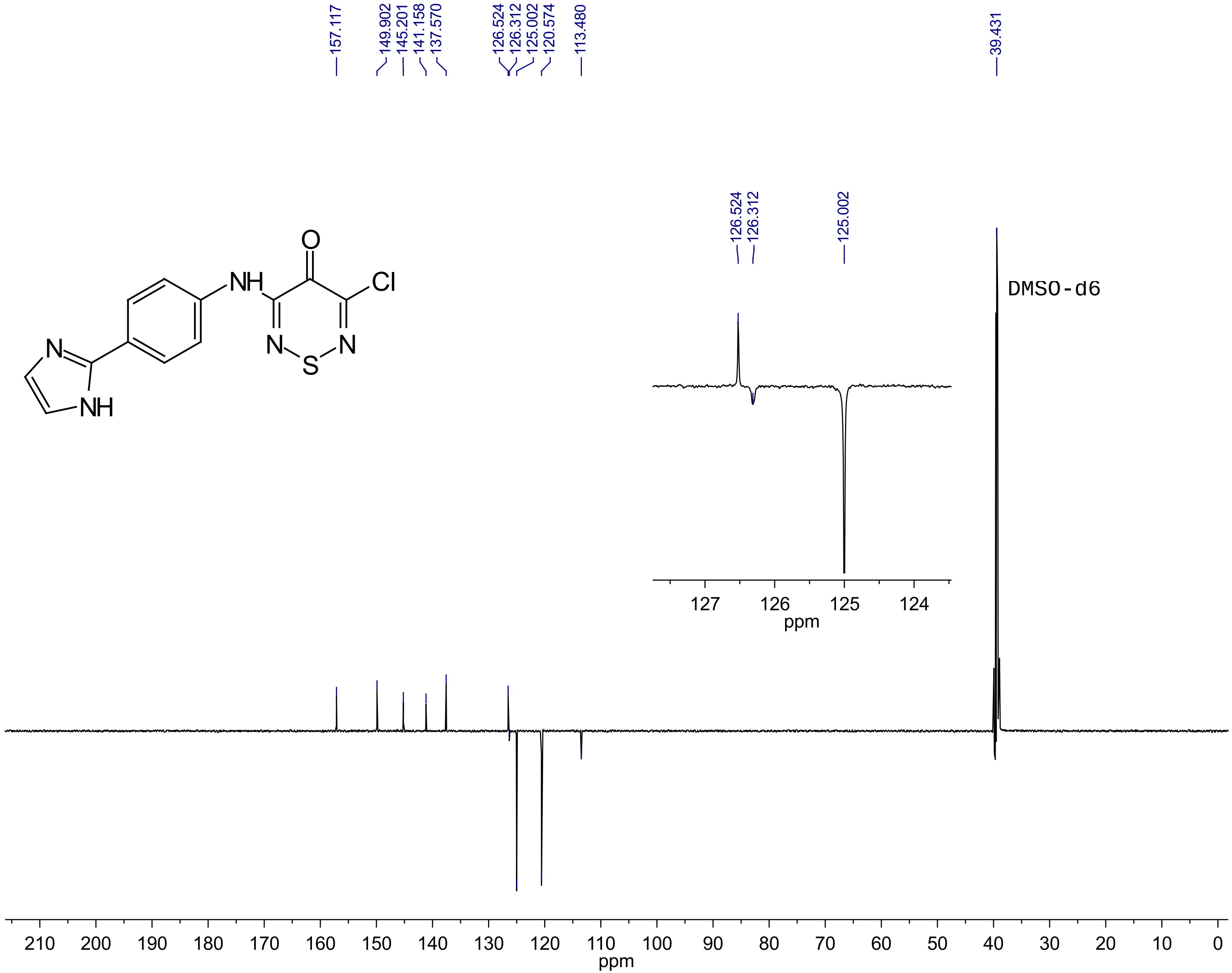

Current Data Parameters
NAME Kalogirou
EXPNO 418
PROCNO 1
F2 - Acquisition Parameters
Date_ 20170923
Time 8.55
INSTRUM spect
PROBHD 5 mm PABBO BB-
PULPROG jmod
TD 65536
SOLVENT DMSO
NS 9216
DS 4
SWH 29761.904 Hz
FIDRES 0.454131 Hz
AQ 1.1010048 sec
RG 2050
DW 16.800 usec
DE 6.50 usec
TE 297.8 K
CNST2 145.0000000
CNST11 1.0000000
D1 2.00000000 sec
D20 0.00689655 sec
TD0 1
======== CHANNEL f1 ========
SFO1 125.7459782 MHz
NUC1 13C
P1 9.00 usec
P2 18.00 usec
PLW1 140.00000000 W
======== CHANNEL f2 ========
SFO2 500.0350280 MHz
NUC2 1H
CPDPRG[2 waltz16
PCPD2 80.00 usec
PLW2 14.50000000 W
PLW12 0.32624999 W
F2 - Processing parameters
SI 32768
SF 125.7334766 MHz
WDW EM
SSB 0
LB 1.00 Hz
GB 0
PC 1.40

^{1}H-NMR of 3-((5-((3-hydroxy-4-methylphenyl)amino)-4-oxo-4*H*-1,2,6-thiadiazin-3-yl)amino)-benzamide (**1**)

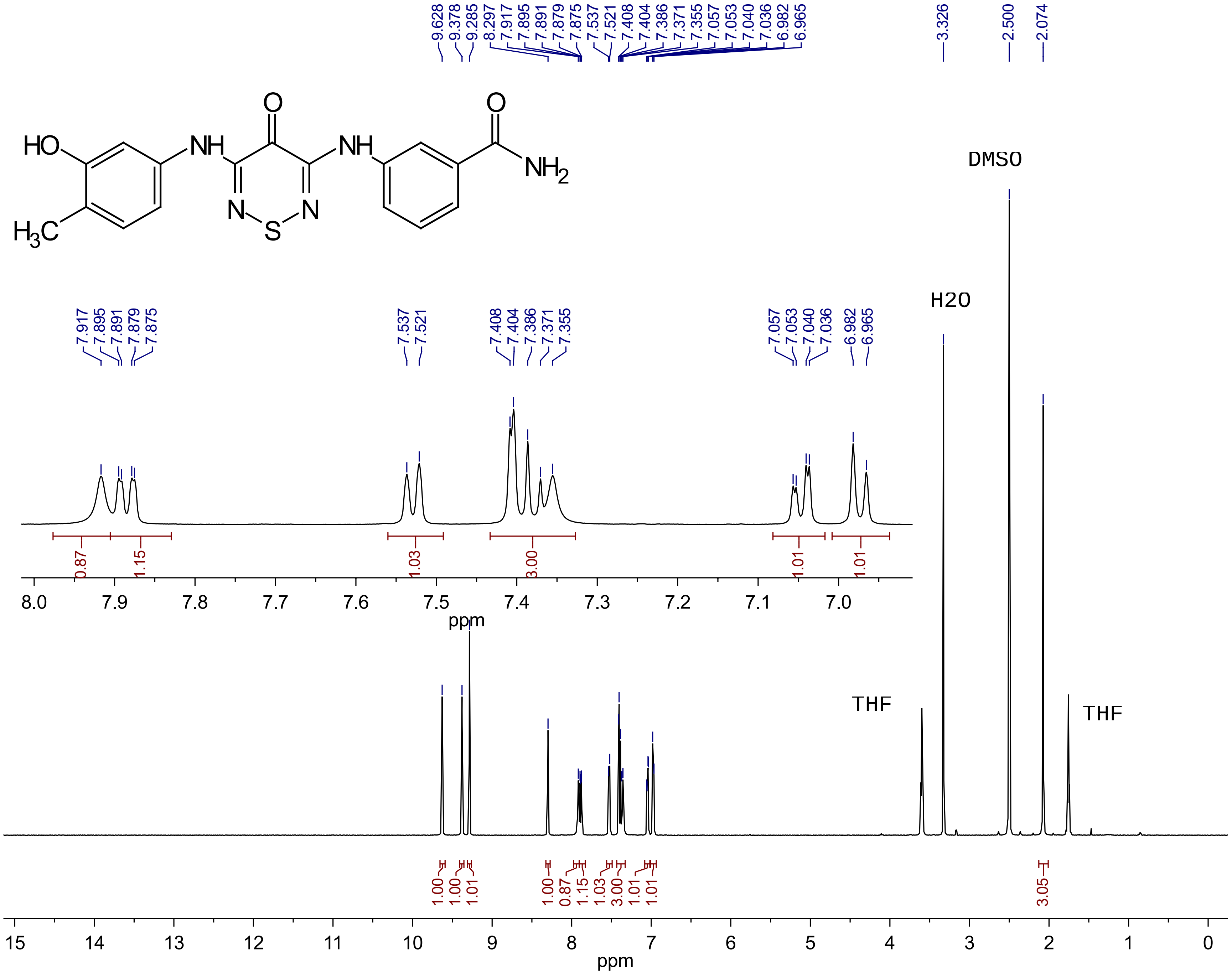

Current Data Parameters
NAME Kalogirou
EXPNO 50
PROCNO 1

F2 - Acquisition Parameters
Date_ 20160520
Time 15.04
INSTRUM spect
PROBHD 5 mm PABBO BB-
PULPROG zg30
TD 65536
SOLVENT DMSO
NS 16
DS 2
SWH 10000.000 Hz
FIDRES 0.152588 Hz
AQ 3.2767999 sec
RG 144
DW 50.000 usec
DE 6.50 usec
TE 297.9 K
D1 1.00000000 sec
TD0 1

CHANNEL f1
SFO1 500.0361158 MHz
NUC1 1H
P1 12.00 usec
PLW1 14.50000000 W

F2 - Processing parameters
SI 65536
SF 500.0330320 MHz
WDW EM
SSB 0
LB 0.30 Hz
GB 0
PC 1.00

^{13}C-NMR of 3-((5-((3-hydroxy-4-methylphenyl)amino)-4-oxo-4*H*-1,2,6-thiadiazin-3-yl)amino)-benzamide (**1**)

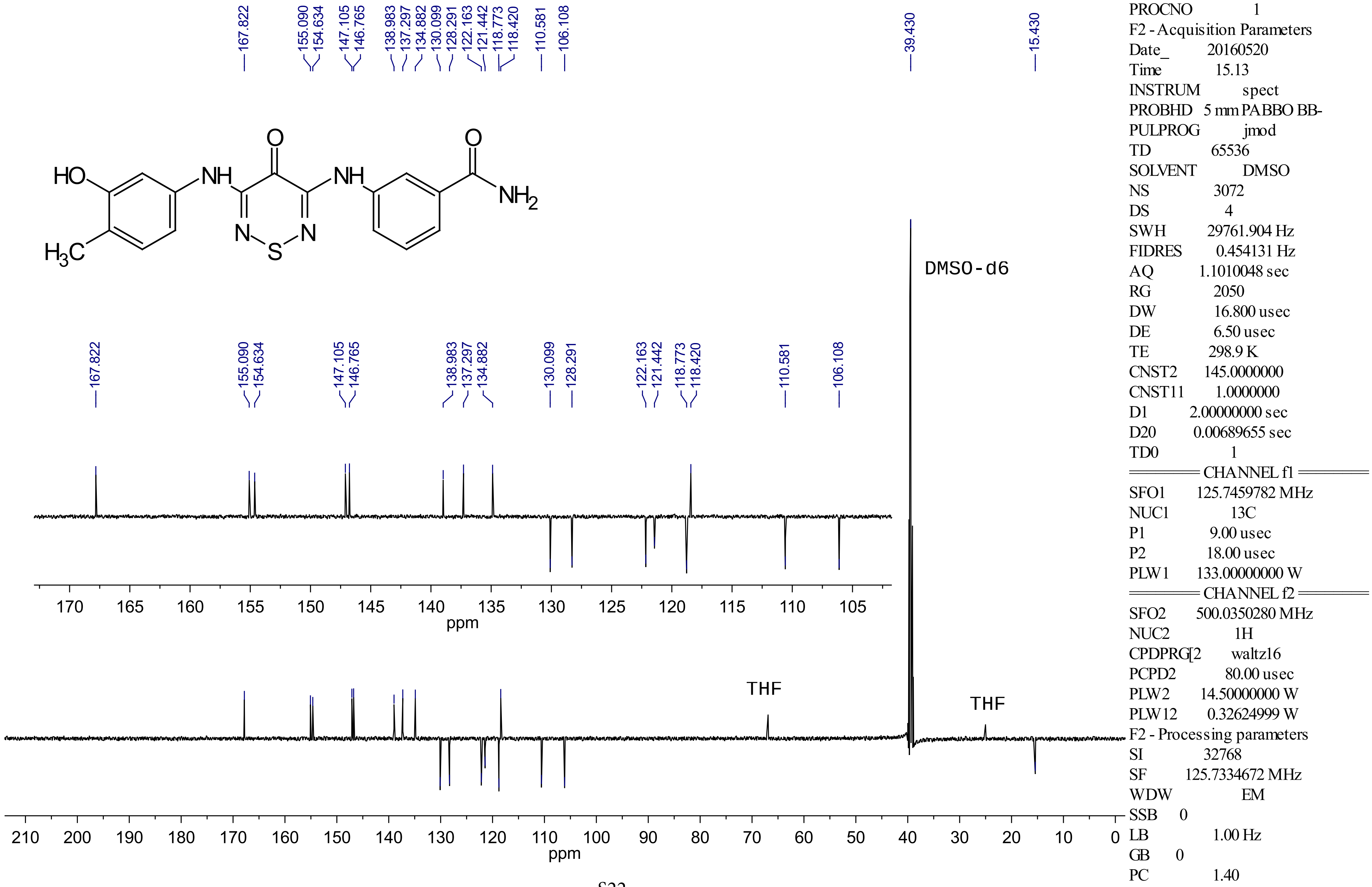

^{1}H-NMR of 4-((5-((3-hydroxy-4-methylphenyl)amino)-4-oxo-4*H*-1,2,6-thiadiazin-3-yl)amino)-benzamide (**2**)

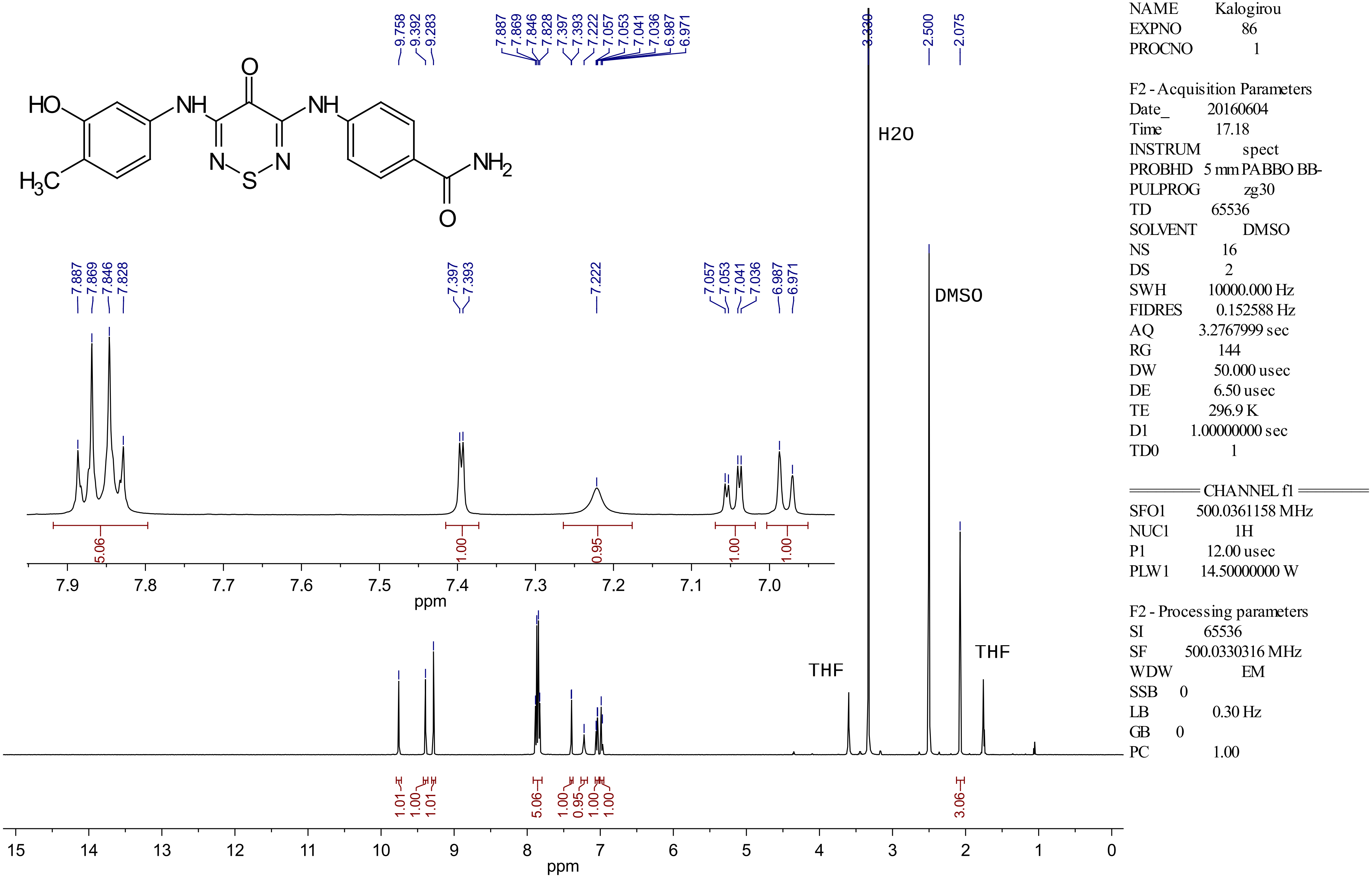

^{13}C-NMR of 4-((5-((3-hydroxy-4-methylphenyl)amino)-4-oxo-4*H*-1,2,6-thiadiazin-3-yl)amino)-benzamide (**2**)

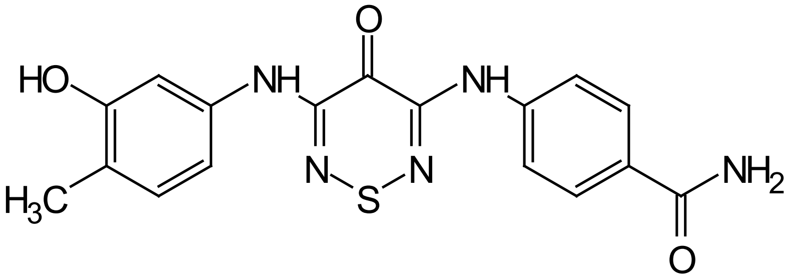

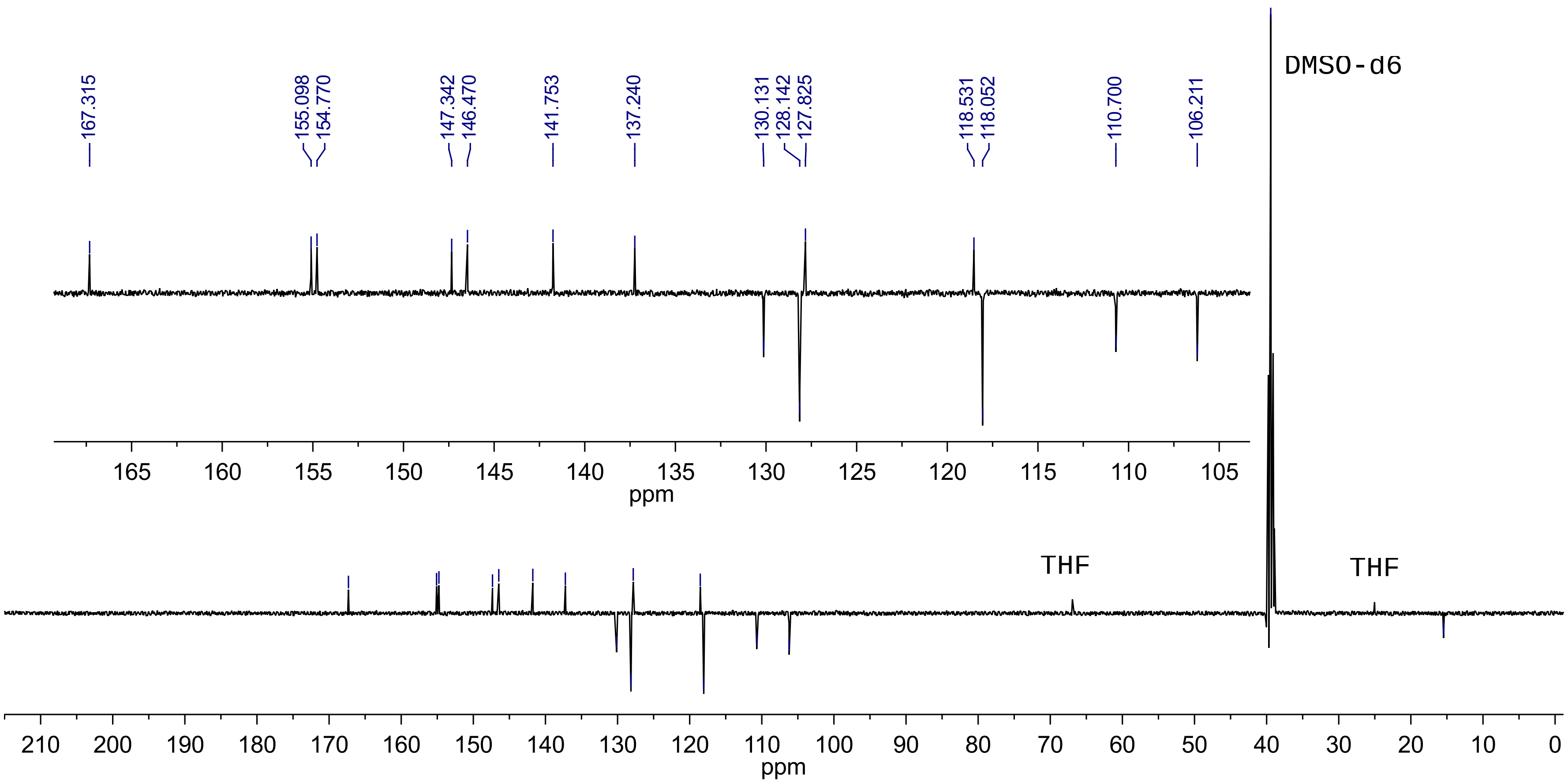

Current Data Parameters
NAME Kalogirou
EXPNO 87
PROCNO 1
F2 - Acquisition Parameters
Date_ 20160604
Time 17.36
INSTRUM spect
PROBHD 5 mm PABBO BB-
PULPROG jmod
TD 65536
SOLVENT DMSO
NS 3072
DS 4
SWH 29761.904 Hz
FIDRES 0.454131 Hz
AQ 1.1010048 sec
RG 2050
DW 16.800 usec
DE 6.50 usec
TE 297.8 K
CNST2 145.0000000
CNST11 1.0000000
D1 2.00000000 sec
D20 0.00689655 sec
TD0 1
======== CHANNEL f1 ========
SFO1 125.7459782 MHz
NUC1 13C
P1 9.00 usec
P2 18.00 usec
PLW1 133.00000000 W
======== CHANNEL f2 ========
SFO2 500.0350280 MHz
NUC2 1H
CPDPRG[2 waltz16
PCPD2 80.00 usec
PLW2 14.50000000 W
PLW12 0.32624999 W
F2 - Processing parameters
SI 32768
SF 125.7334644 MHz
WDW EM
SSB 0
LB 1.00 Hz
GB 0
PC 1.40

^{1}H-NMR of 3-((2,2-dioxido-1,3-dihydrobenzo[*c*]thiophen-5-yl)amino)-5-((3-hydroxy-4-methyl-phenyl) amino)-4*H*-1,2,6-thiadiazin-4-one (**3**)

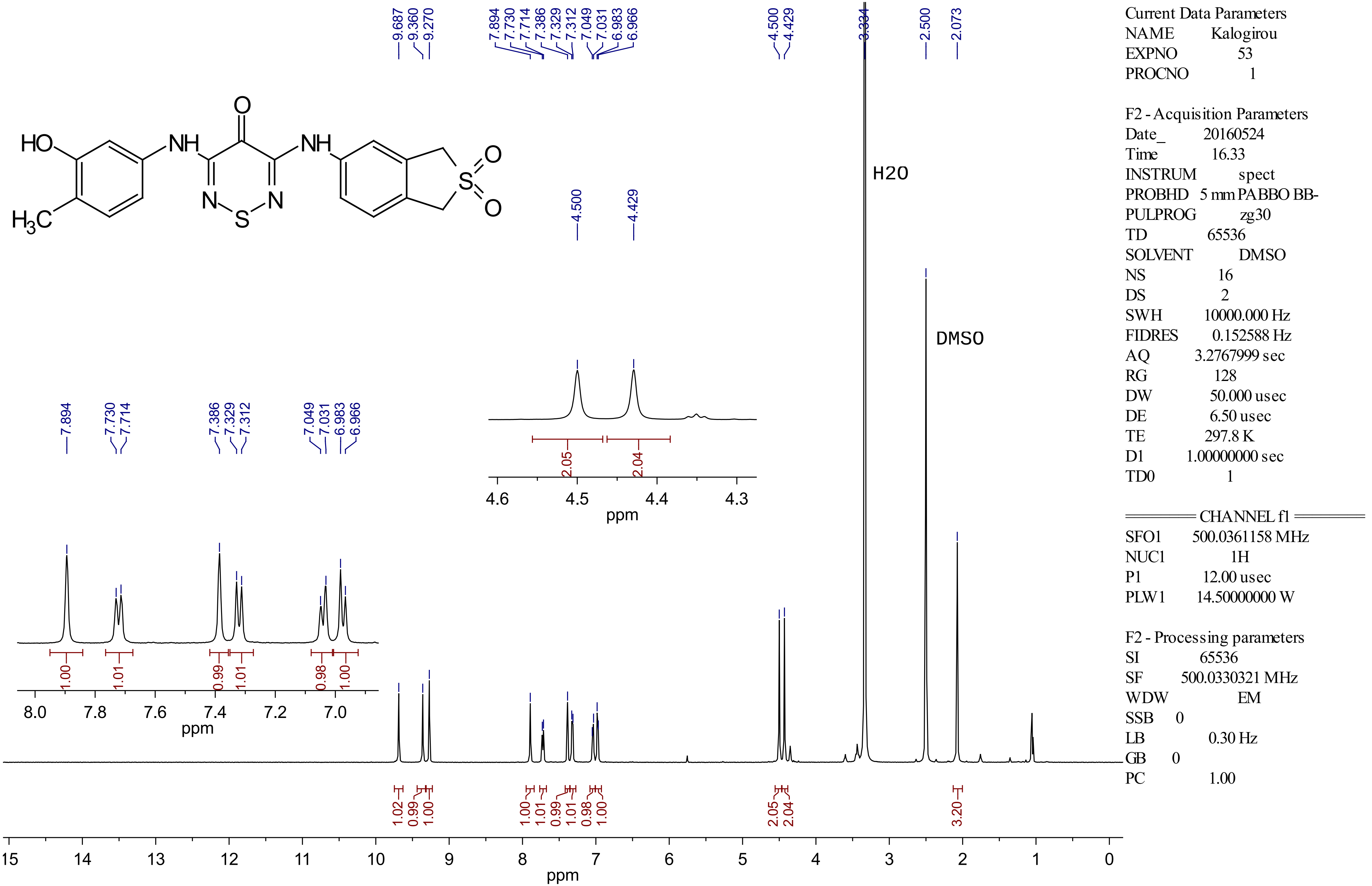

^{13}C-NMR of 3-((2,2-dioxido-1,3-dihydrobenzo[*c*]thiophen-5-yl)amino)-5-((3-hydroxy-4-methyl-phenyl) amino)-4*H*-1,2,6-thiadiazin-4-one (**3**)

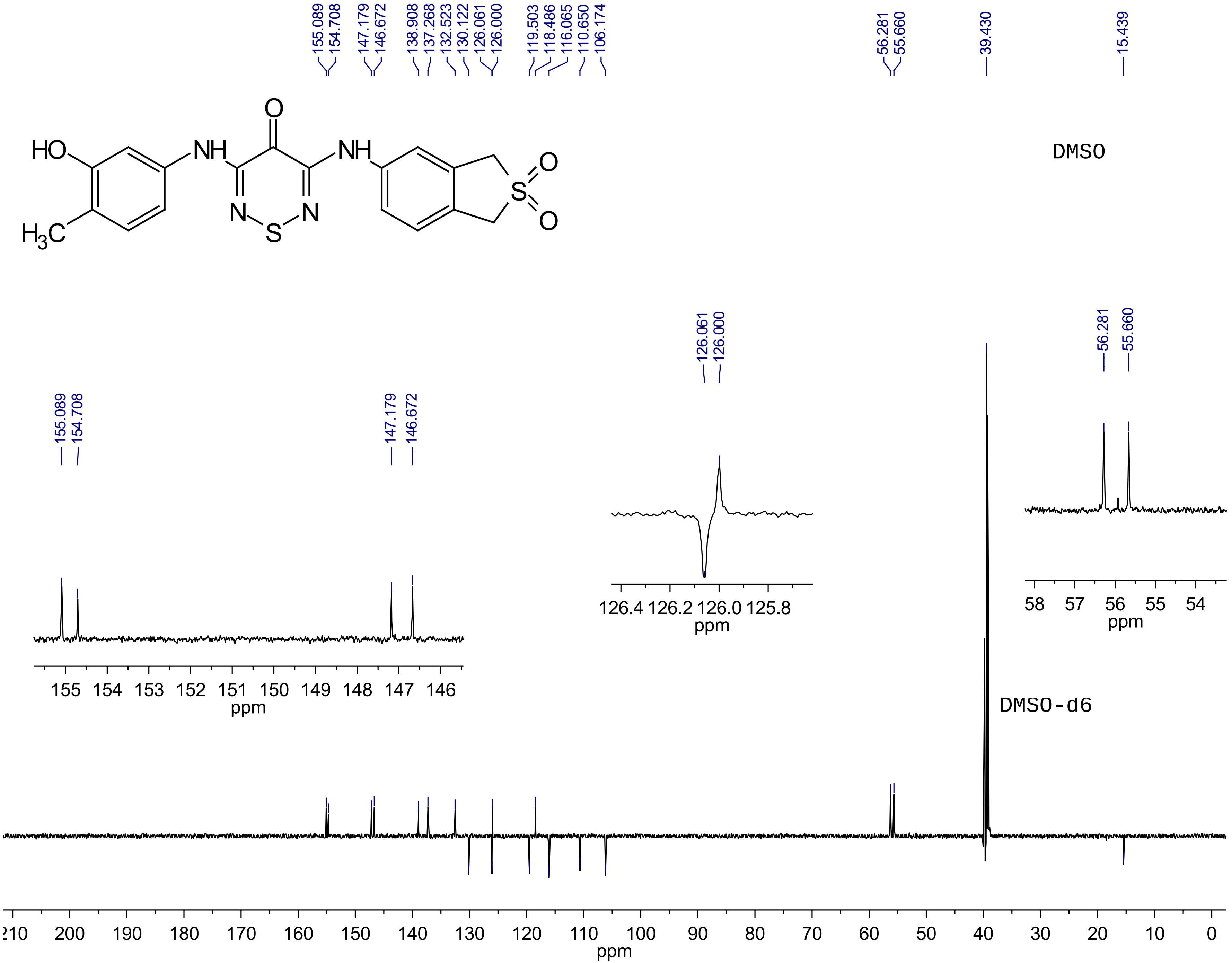

Current Data Parameters
NAME Kalogirou
EXPNO 54
PROCNO 1
F2 - Acquisition Parameters
Date_ 20160524
Time 19.16
INSTRUM spect
PROBHD 5 mm PABBO BB-
PULPROG jmod
TD 65536
SOLVENT DMSO
NS 3072
DS 4
SWH 29761.904 Hz
FIDRES 0.454131 Hz
AQ 1.1010048 sec
RG 2050
DW 16.800 usec
DE 6.50 usec
TE 298.9 K
CNST2 145.0000000
CNST11 1.0000000
D1 2.00000000 sec
D20 0.00689655 sec
TD0 1
CHANNEL f1
SFO1 125.7459782 MHz
NUC1 13C
P1 9.00 usec
P2 18.00 usec
PLW1 133.00000000 W
CHANNEL f2
SFO2 500.0350280 MHz
NUC2 1H
CPDPRG[2 waltz16
PCPD2 80.00 usec
PLW2 14.50000000 W
PLW12 0.32624999 W
F2 - Processing parameters
SI 32768
SF 125.7334652 MHz
WDW EM
SSB 0
LB 1.00 Hz
GB 0
PC 1.40

^{1}H-NMR of *N*-methyl-2-((5-((3-((methylsulfonyl)methyl)phenyl)amino)-4-oxo-4*H*-1,2,6-thiadiazin-3-yl)-amino)benzamide (**4**)

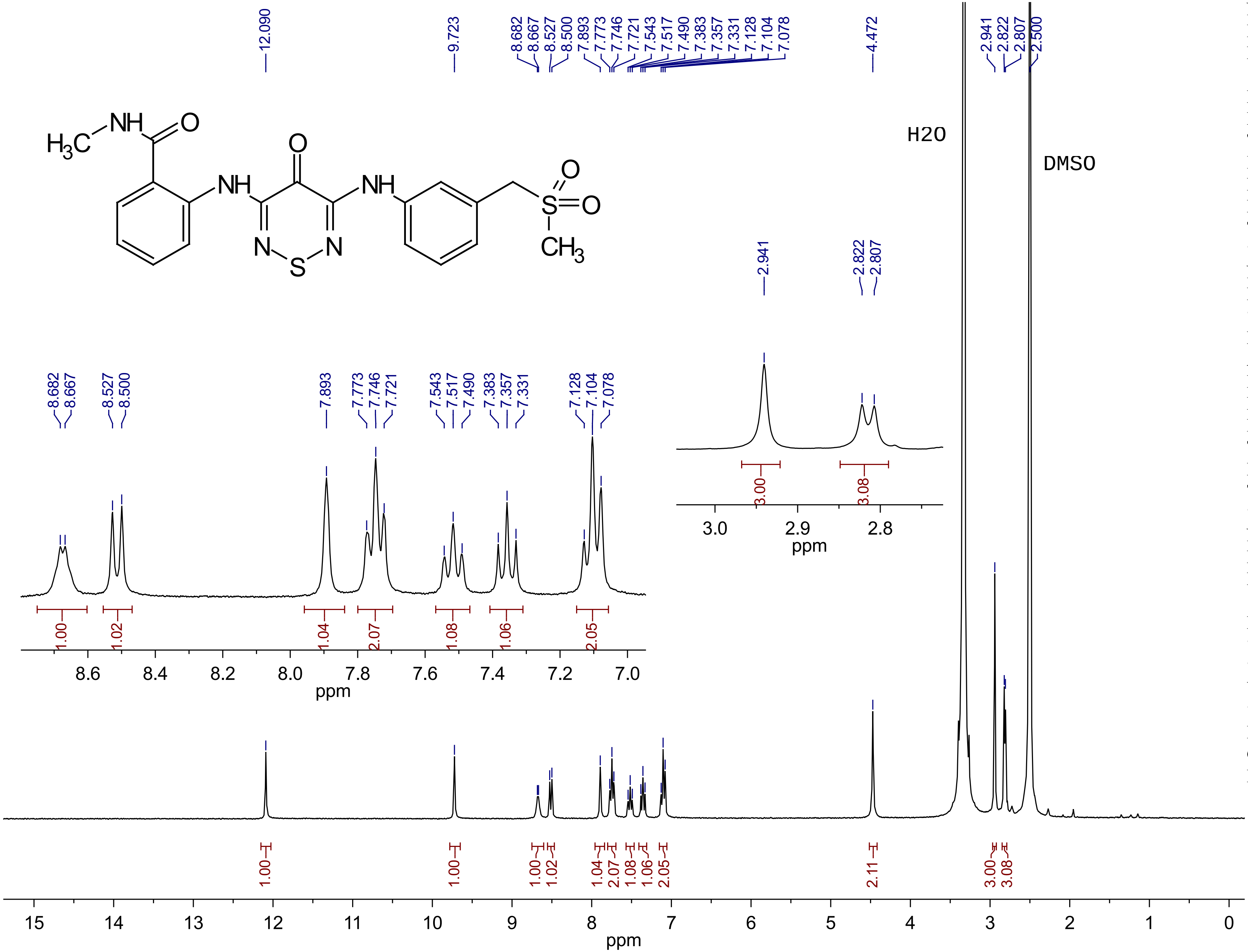

Current Data Parameters
NAME Andreas
EXPNO 114
PROCNO 1

F2 - Acquisition Parameters
Date_ 20180414
Time 18.24 h
INSTRUM spect
PROBHD Z104275_0375 (
PULPROG zg30
TD 65536
SOLVENT DMSO
NS 128
DS 2
SWH 6009.615 Hz
FIDRES 0.183399 Hz
AQ 5.4525952 sec
RG 201.81
DW 83.200 usec
DE 6.50 usec
TE 298.1 K
D1 1.00000000 sec
TD0 1
SFO1 300.1318533 MHz
NUC1 1H
P1 14.00 usec
PLW1 7.50000000 W

F2 - Processing parameters
SI 65536
SF 300.1300024 MHz
WDW EM
SSB 0
LB 0.30 Hz
GB 0
PC 1.00

^{13}C-NMR of *N*-methyl-2-((5-((3-((methylsulfonyl)methyl)phenyl)amino)-4-oxo-4*H*-1,2,6-thiadiazin-3-yl)-amino)benzamide (**4**)

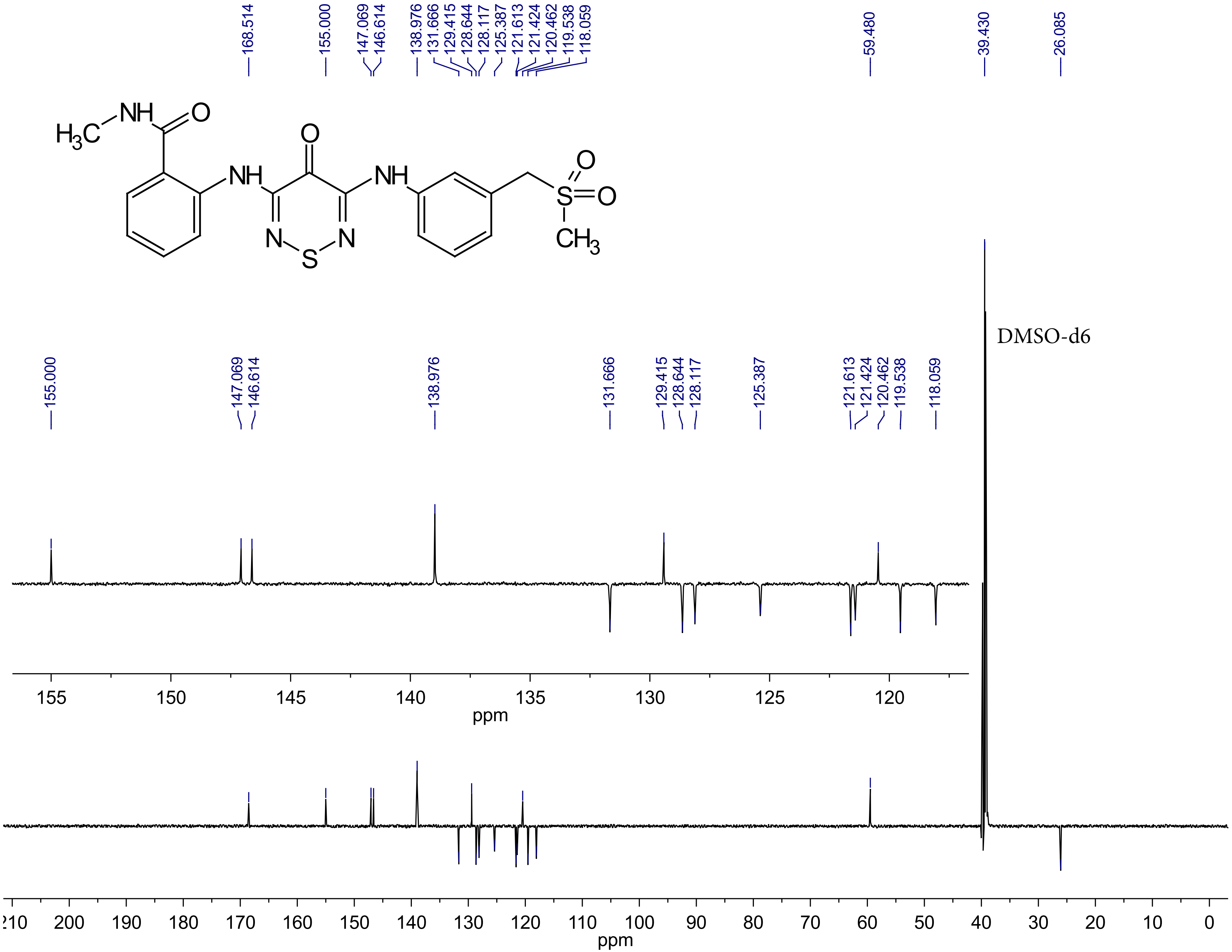

Current Data Parameters
NAME Kalogirou
EXPNO 56
PROCNO 1
F2 - Acquisition Parameters
Date_ 20160525
Time 5.16
INSTRUM spect
PROBHD 5 mm PABBO BB-
PULPROG jmod
TD 65536
SOLVENT DMSO
NS 10240
DS 4
SWH 29761.904 Hz
FIDRES 0.454131 Hz
AQ 1.1010048 sec
RG 2050
DW 16.800 usec
DE 6.50 usec
TE 299.2 K
CNST2 145.0000000
CNST11 1.0000000
D1 2.00000000 sec
D20 0.00689655 sec
TD0 1
======== CHANNEL f1 ========
SFO1 125.7459782 MHz
NUC1 13C
P1 9.00 usec
P2 18.00 usec
PLW1 133.00000000 W
======== CHANNEL f2 ========
SFO2 500.0350280 MHz
NUC2 1H
CPDPRG[2 waltz16
PCPD2 80.00 usec
PLW2 14.50000000 W
PLW12 0.32624999 W
F2 - Processing parameters
SI 32768
SF 125.7334672 MHz
WDW EM
SSB 0
LB 1.00 Hz
GB 0
PC 1.40

^{1}H-NMR of *N*-methyl-2-((5-((4-morpholinophenyl)amino)-4-oxo-4*H*-1,2,6-thiadiazin-3-yl)amino)- benzamide (**5**)

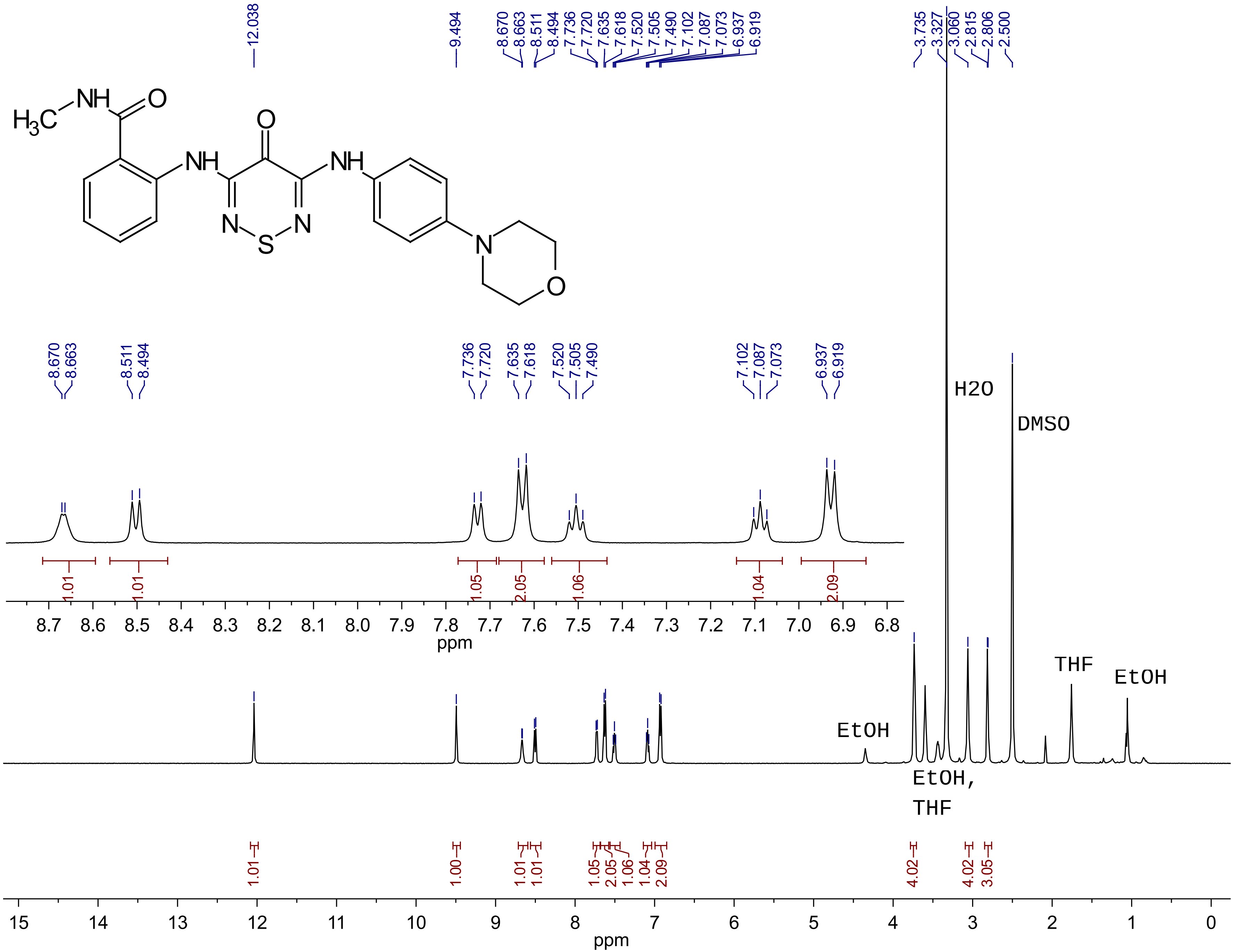

Current Data Parameters
NAME Kalogirou
EXPNO 62
PROCNO 1

F2 - Acquisition Parameters
Date_ 20160526
Time 13.29
INSTRUM spect
PROBHD 5 mm PABBO BB-
PULPROG zg30
TD 65536
SOLVENT DMSO
NS 16
DS 2
SWH 10000.000 Hz
FIDRES 0.152588 Hz
AQ 3.2767999 sec
RG 128
DW 50.000 usec
DE 6.50 usec
TE 297.8 K
D1 1.00000000 sec
TD0 1

CHANNEL f1
SFO1 500.0361158 MHz
NUC1 1H
P1 12.00 usec
PLW1 14.50000000 W

F2 - Processing parameters
SI 65536
SF 500.0330323 MHz
WDW EM
SSB 0
LB 0.30 Hz
GB 0
PC 1.00

^{13}C-NMR of *N*-methyl-2-((5-((4-morpholinophenyl)amino)-4-oxo-4*H*-1,2,6-thiadiazin-3-yl)amino)- benzamide (**5**)

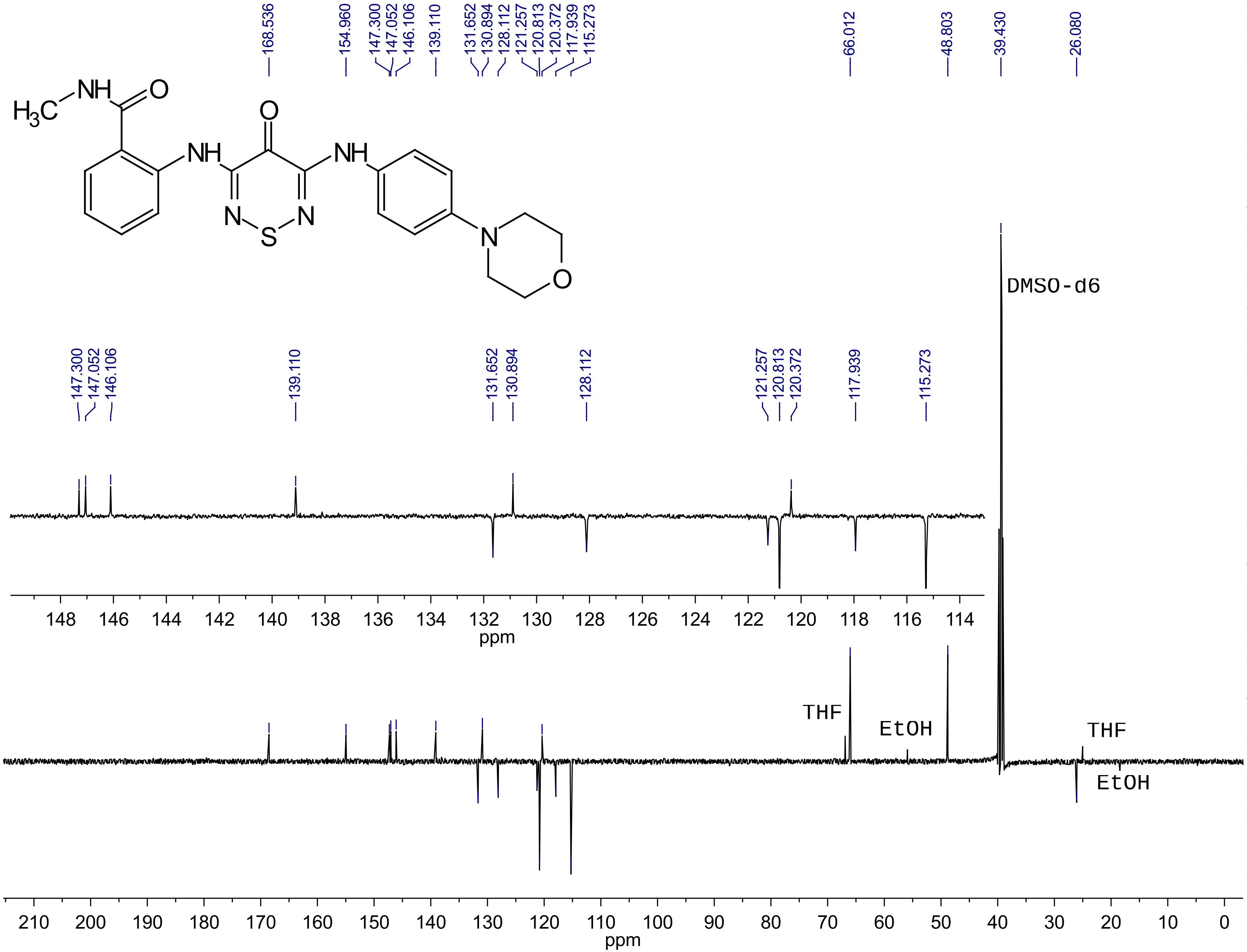

Current Data Parameters
NAME Kalogirou
EXPNO 63
PROCNO 1
F2 - Acquisition Parameters
Date_ 20160526
Time 15.32
INSTRUM spect
PROBHD 5 mm PABBO BB-
PULPROG jmod
TD 65536
SOLVENT DMSO
NS 2666
DS 4
SWH 29761.904 Hz
FIDRES 0.454131 Hz
AQ 1.1010048 sec
RG 2050
DW 16.800 usec
DE 6.50 usec
TE 299.3 K
CNST2 145.0000000
CNST11 1.0000000
D1 2.00000000 sec
D20 0.00689655 sec
TD0 1
======== CHANNEL f1 ========
SFO1 125.7459782 MHz
NUC1 13C
P1 9.00 usec
P2 18.00 usec
PLW1 133.00000000 W
======== CHANNEL f2 ========
SFO2 500.0350280 MHz
NUC2 1H
CPDPRG[2 waltz16
PCPD2 80.00 usec
PLW2 14.50000000 W
PLW12 0.32624999 W
F2 - Processing parameters
SI 32768
SF 125.7334666 MHz
WDW EM
SSB 0
LB 1.00 Hz
GB 0
PC 1.40

^{1}H-NMR of 2-((5-((3-hydroxy-4-methylphenyl)amino)-4-oxo-4*H*-1,2,6-thiadiazin-3-yl)amino)-benzamide (**10**)

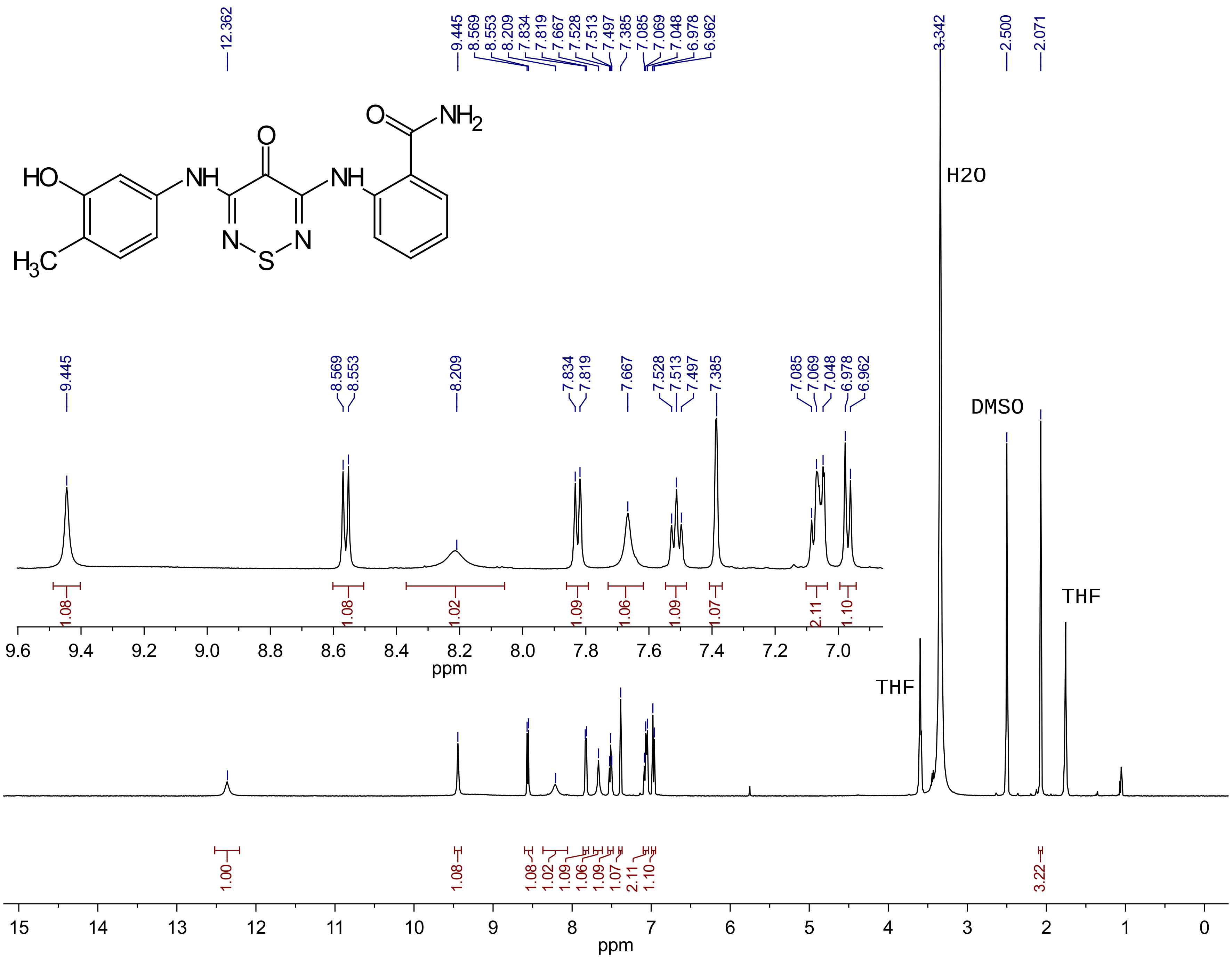

Current Data Parameters
NAME Kalogirou
EXPNO 344
PROCNO 1

F2 - Acquisition Parameters
Date_ 20170627
Time 23.56
INSTRUM spect
PROBHD 5 mm PABBO BB-
PULPROG zg30
TD 65536
SOLVENT DMSO
NS 16
DS 2
SWH 10000.000 Hz
FIDRES 0.152588 Hz
AQ 3.2767999 sec
RG 114
DW 50.000 usec
DE 6.50 usec
TE 297.7 K
D1 1.00000000 sec
TD0 1

======== CHANNEL f1 ========
SFO1 500.0361158 MHz
NUC1 1H
P1 12.00 usec
PLW1 14.50000000 W

F2 - Processing parameters
SI 65536
SF 500.0330322 MHz
WDW EM
SSB 0
LB 0.30 Hz
GB 0
PC 1.00

^{13}C-NMR of 2-((5-((3-hydroxy-4-methylphenyl)amino)-4-oxo-4*H*-1,2,6-thiadiazin-3-yl)amino)-benzamide (**10**)

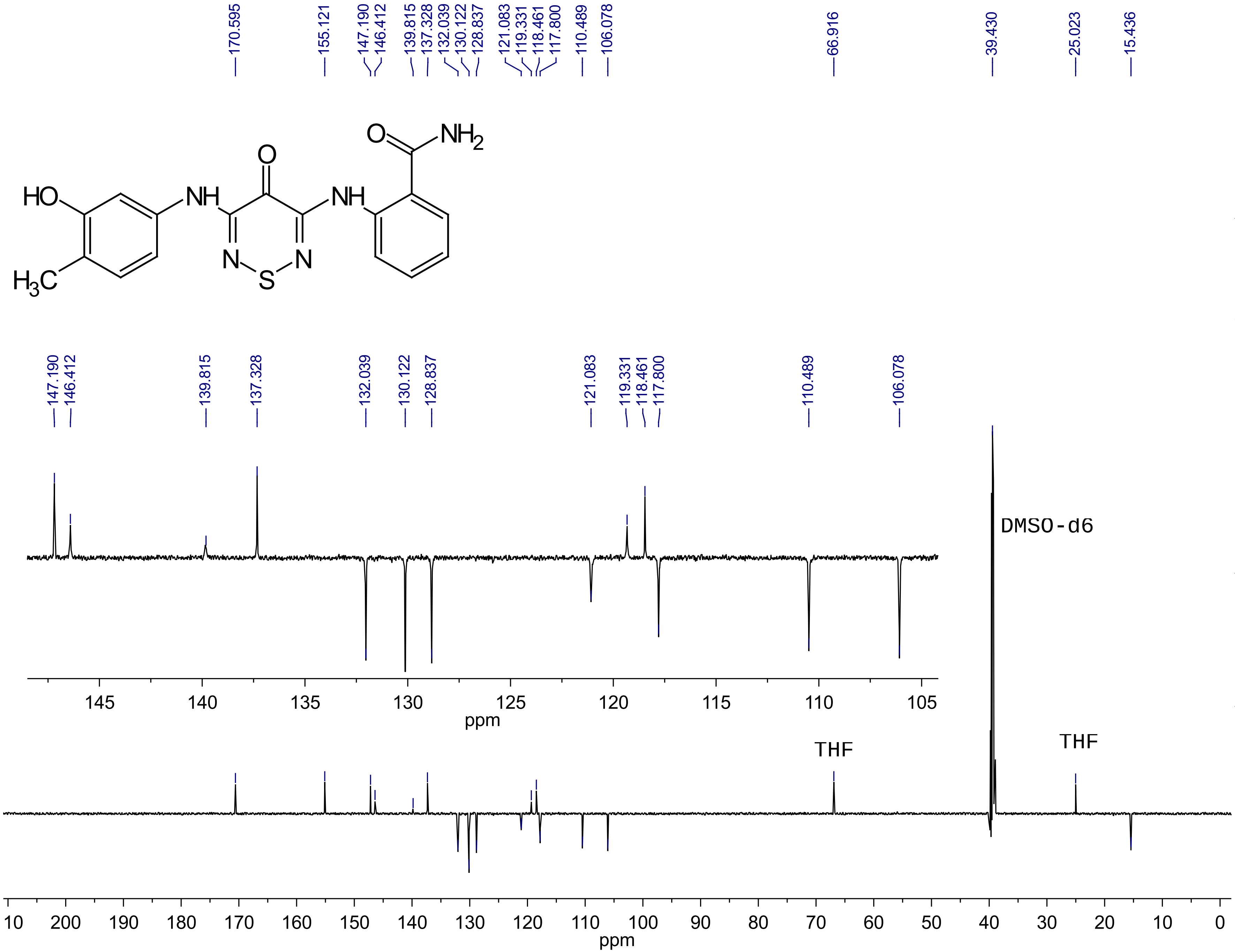

Current Data Parameters
NAME Kalogirou
EXPNO 345
PROCNO 1
F2 - Acquisition Parameters
Date_ 20170628
Time 8.55
INSTRUM spect
PROBHD 5 mm PABBO BB-
PULPROG jmod
TD 65536
SOLVENT DMSO
NS 10240
DS 4
SWH 29761.904 Hz
FIDRES 0.454131 Hz
AQ 1.1010048 sec
RG 2050
DW 16.800 usec
DE 6.50 usec
TE 299.5 K
CNST2 145.0000000
CNST11 1.0000000
D1 2.00000000 sec
D20 0.00689655 sec
TD0 1
CHANNEL f1
SFO1 125.7459782 MHz
NUC1 13C
P1 9.00 usec
P2 18.00 usec
PLW1 140.00000000 W
CHANNEL f2
SFO2 500.0350280 MHz
NUC2 1H
CPDPRG[2 waltz16
PCPD2 80.00 usec
PLW2 14.50000000 W
PLW12 0.32624999 W
F2 - Processing parameters
SI 32768
SF 125.7334769 MHz
WDW EM
SSB 0
LB 1.00 Hz
GB 0
PC 1.40

^{1}H-NMR of 3-((4-(1*H*-imidazol-2-yl)phenyl)amino)-5-((3-hydroxy-4-methylphenyl)amino)-4*H*-1,2,6-thiadiazin-4-one (**11**)

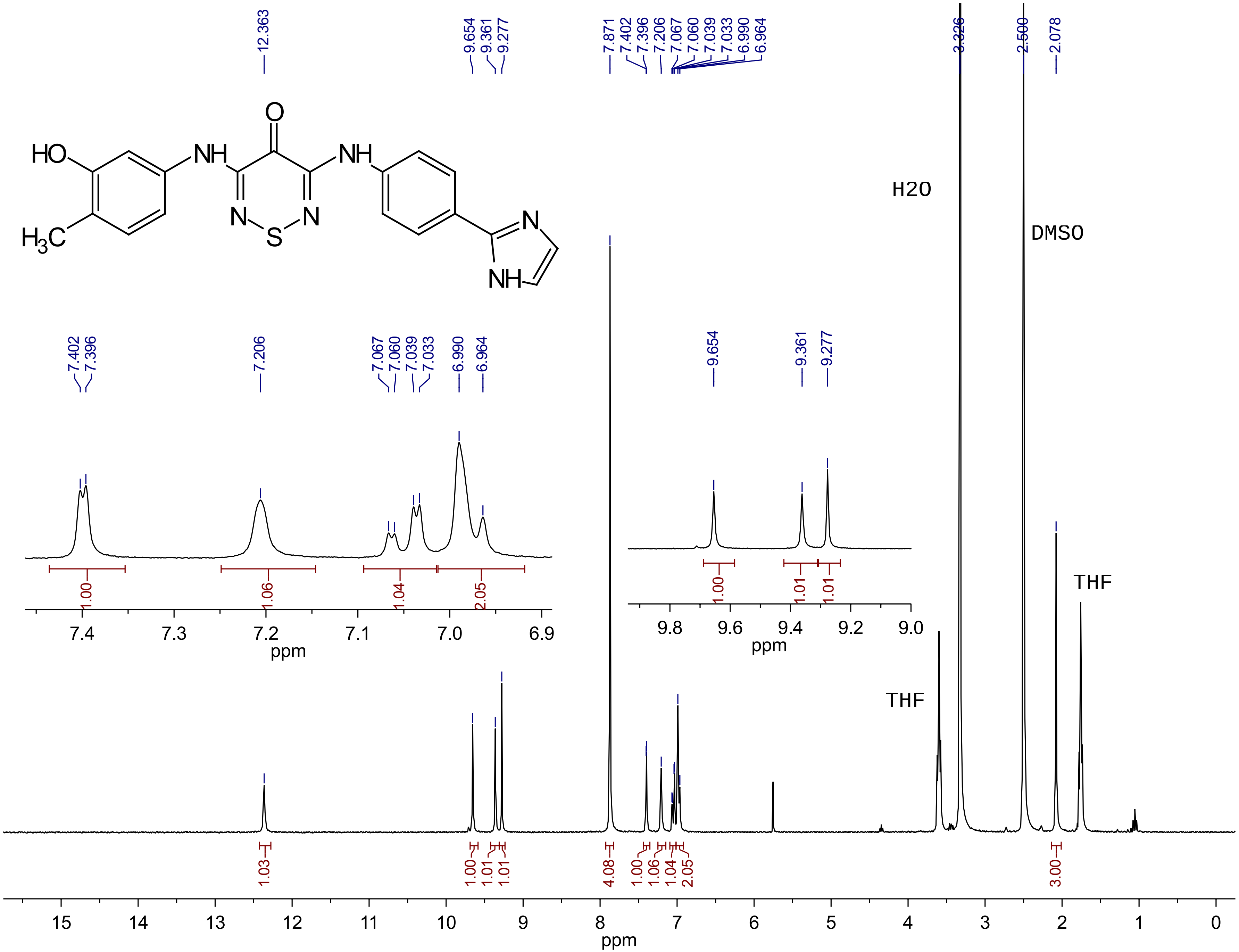

Current Data Parameters
NAME Andreas
EXPNO 47
PROCNO 1

F2 - Acquisition Parameters
Date_ 20170921
Time 19.28 h
INSTRUM spect
PROBHD Z104275_0375 (
PULPROG zg30
TD 65536
SOLVENT DMSO
NS 16
DS 2
SWH 6009.615 Hz
FIDRES 0.183399 Hz
AQ 5.4525952 sec
RG 201.81
DW 83.200 usec
DE 6.50 usec
TE 298.2 K
D1 1.00000000 sec
TD0 1
SFO1 300.1318533 MHz
NUC1 1H
P1 14.00 usec
PLW1 7.50000000 W

F2 - Processing parameters
SI 65536
SF 300.1300032 MHz
WDW EM
SSB 0
LB 0.30 Hz
GB 0
PC 1.00

^{13}C-NMR of 3-((4-(1*H*-imidazol-2-yl)phenyl)amino)-5-((3-hydroxy-4-methylphenyl)amino)-4*H*-1,2,6-thiadiazin-4-one (**11**)

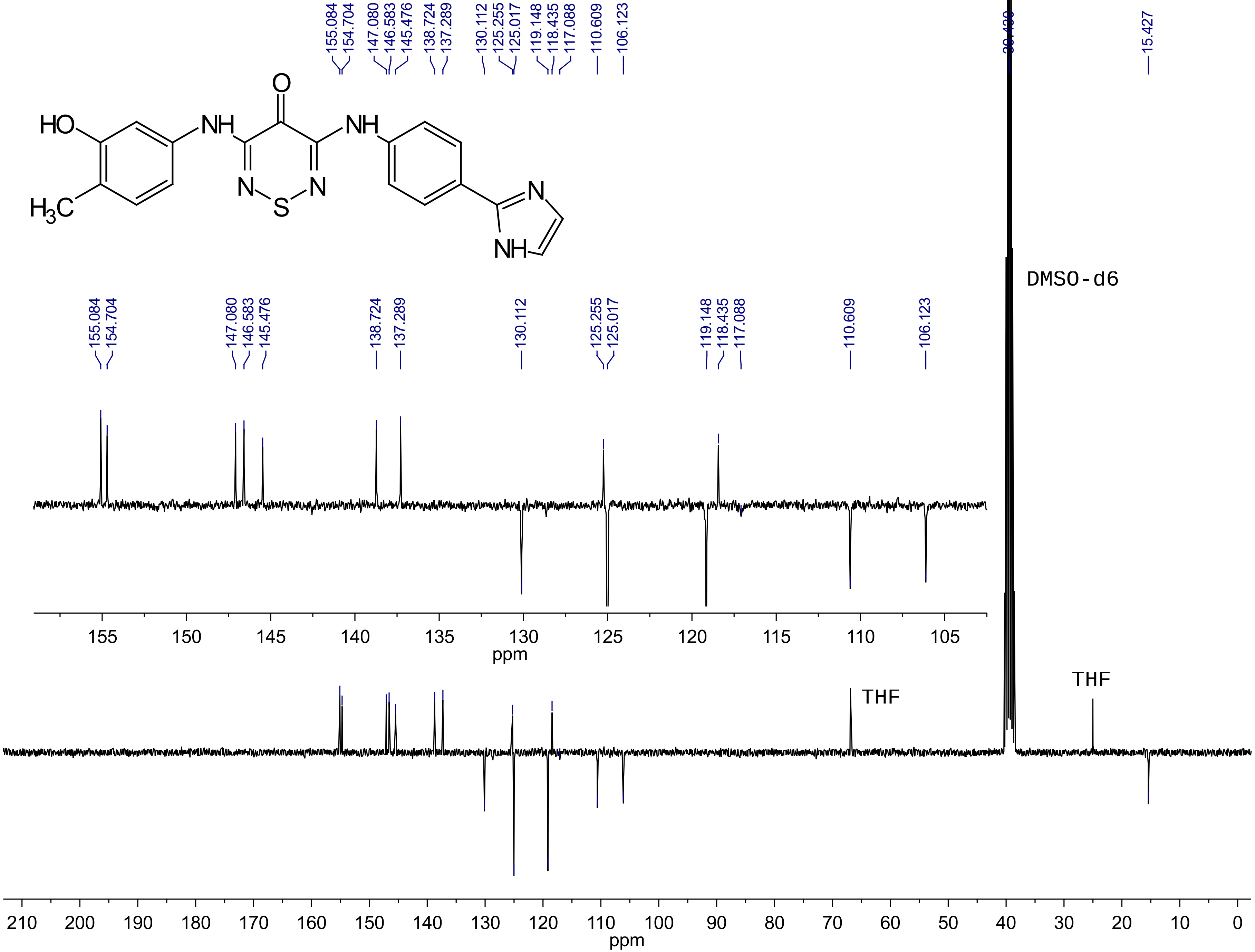

Current Data Parameters
NAME Andreas
EXPNO 48
PROCNO 1
F2 - Acquisition Parameters
Date_ 20170922
Time 9.51 h
INSTRUM spect
PROBHD Z104275_0375 (
PULPROG jmod
TD 65536
SOLVENT DMSO
NS 13252
DS 4
SWH 18115.941 Hz
FIDRES 0.552855 Hz
AQ 1.8087935 sec
RG 201.81
DW 27.600 usec
DE 6.50 usec
TE 298.4 K
CNST2 145.0000000
CNST11 1.0000000
D1 2.00000000 sec
D20 0.00689655 sec
TD0 1
SFO1 75.4752953 MHz
NUC1 13C
P1 10.00 usec
P2 20.00 usec
PLW1 40.05500031 W
SFO2 300.1312005 MHz
NUC2 1H
CPDPRG[2 waltz16
PCPD2 90.00 usec
PLW2 7.50000000 W
PLW12 0.18148001 W
F2 - Processing parameters
SI 32768
SF 75.4677930 MHz
WDW EM
SSB 0
LB 1.00 Hz
GB 0
PC 1.40

^{1}H-NMR of 4-((5-((3-hydroxy-4-methylphenyl)amino)-4-oxo-4*H*-1,2,6-thiadiazin-3-yl)amino)-benzoic acid (**12**)

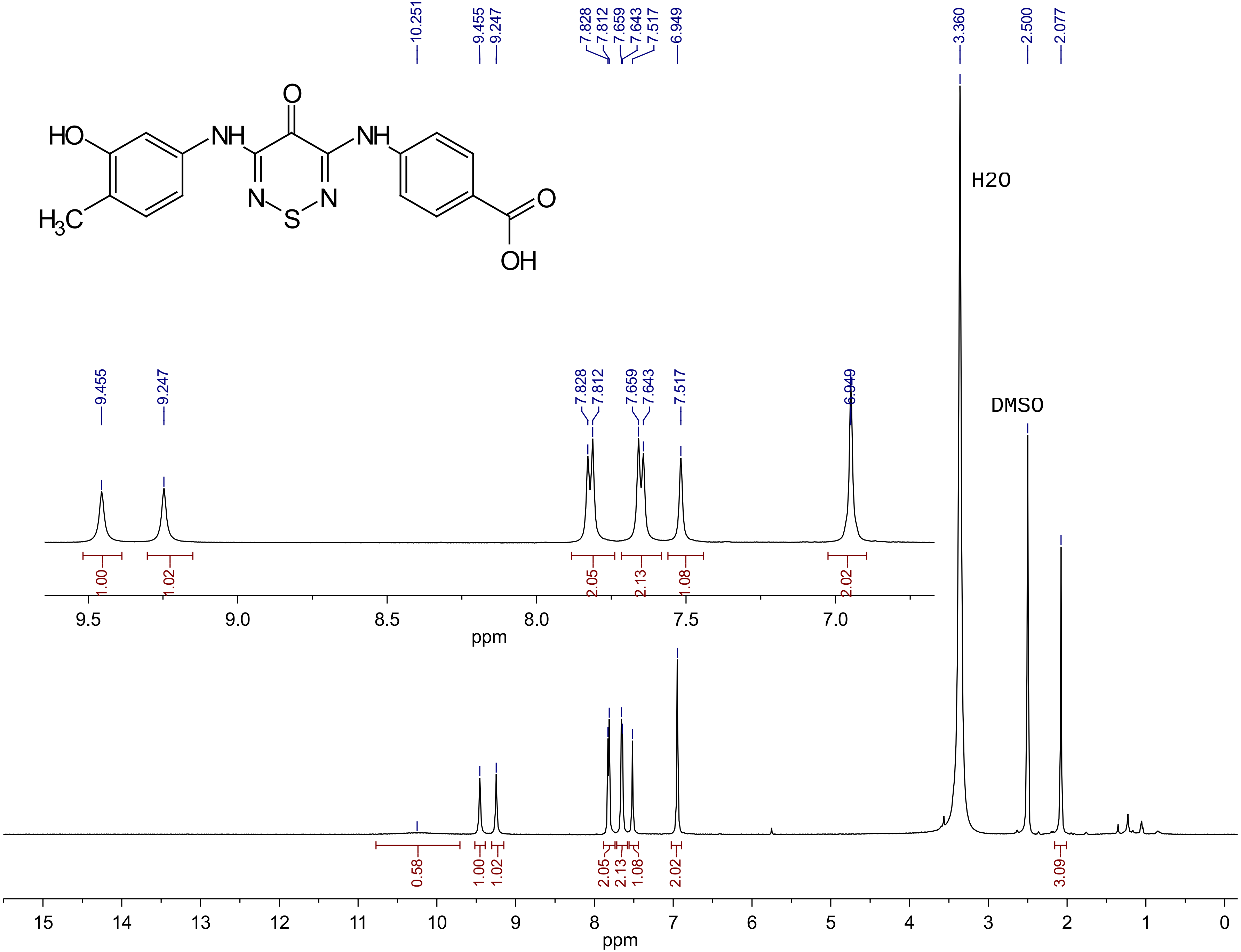

Current Data Parameters
NAME Kalogirou
EXPNO 346
PROCNO 1

F2 - Acquisition Parameters
Date_ 20170629
Time 0.23
INSTRUM spect
PROBHD 5 mm PABBO BB-
PULPROG zg30
TD 65536
SOLVENT DMSO
NS 16
DS 2
SWH 10000.000 Hz
FIDRES 0.152588 Hz
AQ 3.2767999 sec
RG 128
DW 50.000 usec
DE 6.50 usec
TE 298.5 K
D1 1.00000000 sec
TD0 1

======== CHANNEL f1 ========
SFO1 500.0361158 MHz
NUC1 1H
P1 12.00 usec
PLW1 14.50000000 W

F2 - Processing parameters
SI 65536
SF 500.0330325 MHz
WDW EM
SSB 0
LB 0.30 Hz
GB 0
PC 1.00

^{13}C-NMR of 4-((5-((3-hydroxy-4-methylphenyl)amino)-4-oxo-4*H*-1,2,6-thiadiazin-3-yl)amino)-benzoic acid (**12**)

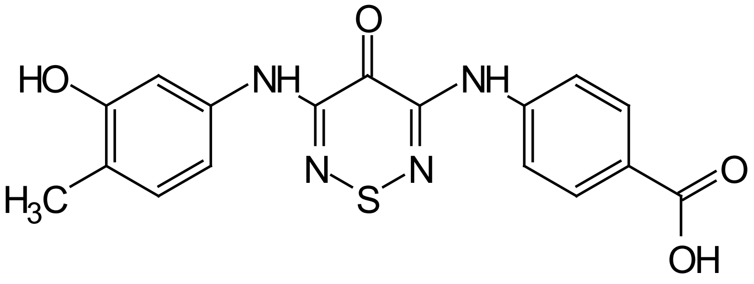

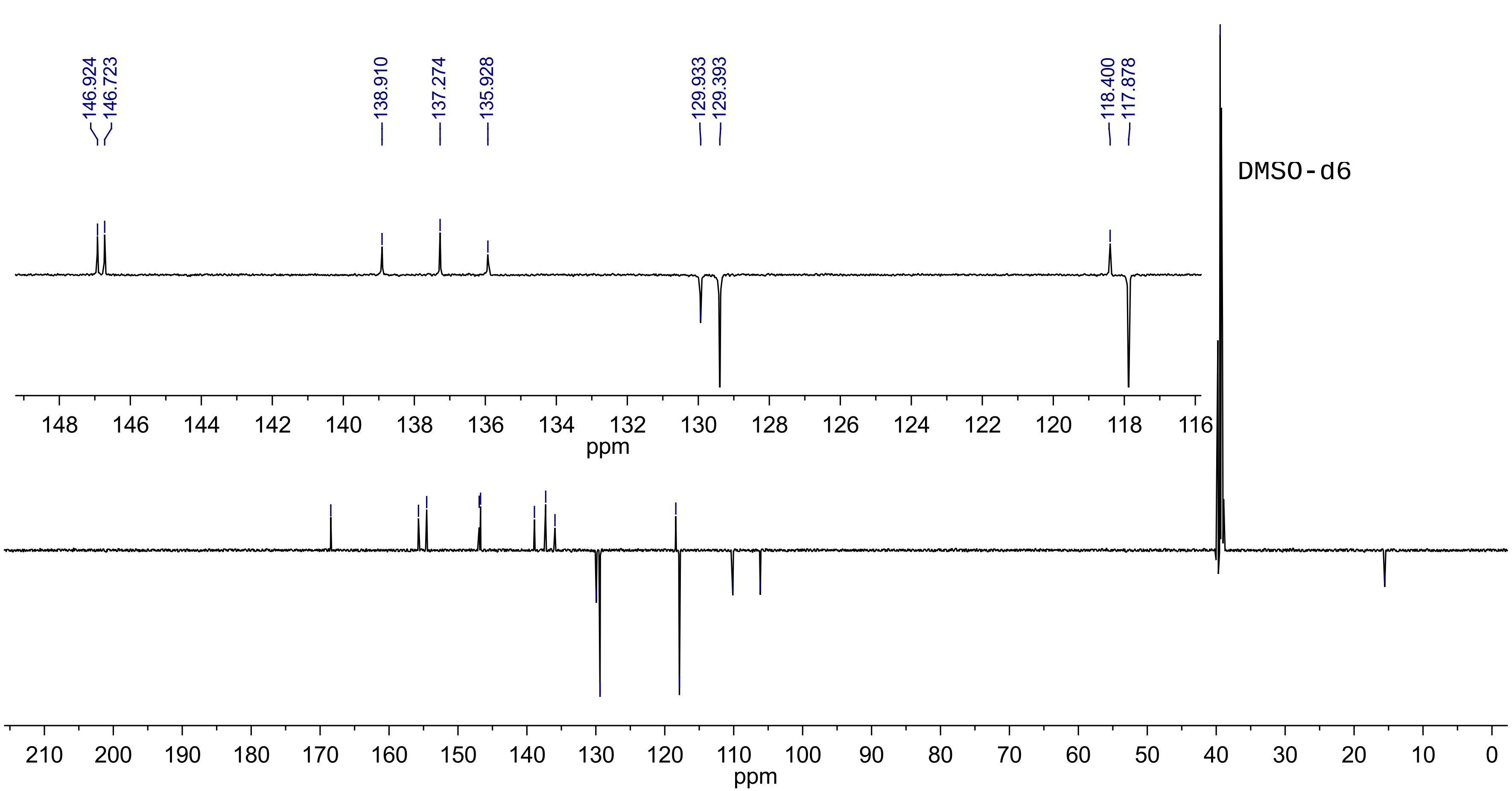

Current Data Parameters
NAME Kalogirou
EXPNO 347
PROCNO 1
F2 - Acquisition Parameters
Date_ 20170629
Time 10.15
INSTRUM spect
PROBHD 5 mm PABBO BB-
PULPROG jmod
TD 65536
SOLVENT DMSO
NS 11264
DS 4
SWH 29761.904 Hz
FIDRES 0.454131 Hz
AQ 1.1010048 sec
RG 1820
DW 16.800 usec
DE 6.50 usec
TE 299.0 K
CNST2 145.0000000
CNST11 1.0000000
D1 2.00000000 sec
D20 0.00689655 sec
TD0 1
======== CHANNEL f1 ========
SFO1 125.7459782 MHz
NUC1 13C
P1 9.00 usec
P2 18.00 usec
PLW1 140.00000000 W
======== CHANNEL f2 ========
SFO2 500.0350280 MHz
NUC2 1H
CPDPRG[2 waltz16
PCPD2 80.00 usec
PLW2 14.50000000 W
PLW12 0.32624999 W
F2 - Processing parameters
SI 32768
SF 125.7334766 MHz
WDW EM
SSB 0
LB 1.00 Hz
GB 0
PC 1.40

^{1}H-NMR of 2-((5-((3-hydroxy-4-methylphenyl)amino)-4-oxo-4*H*-1,2,6-thiadiazin-3-yl)amino)-benzonitrile (**13**)

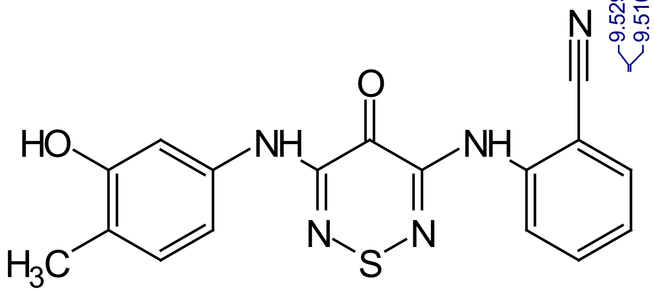

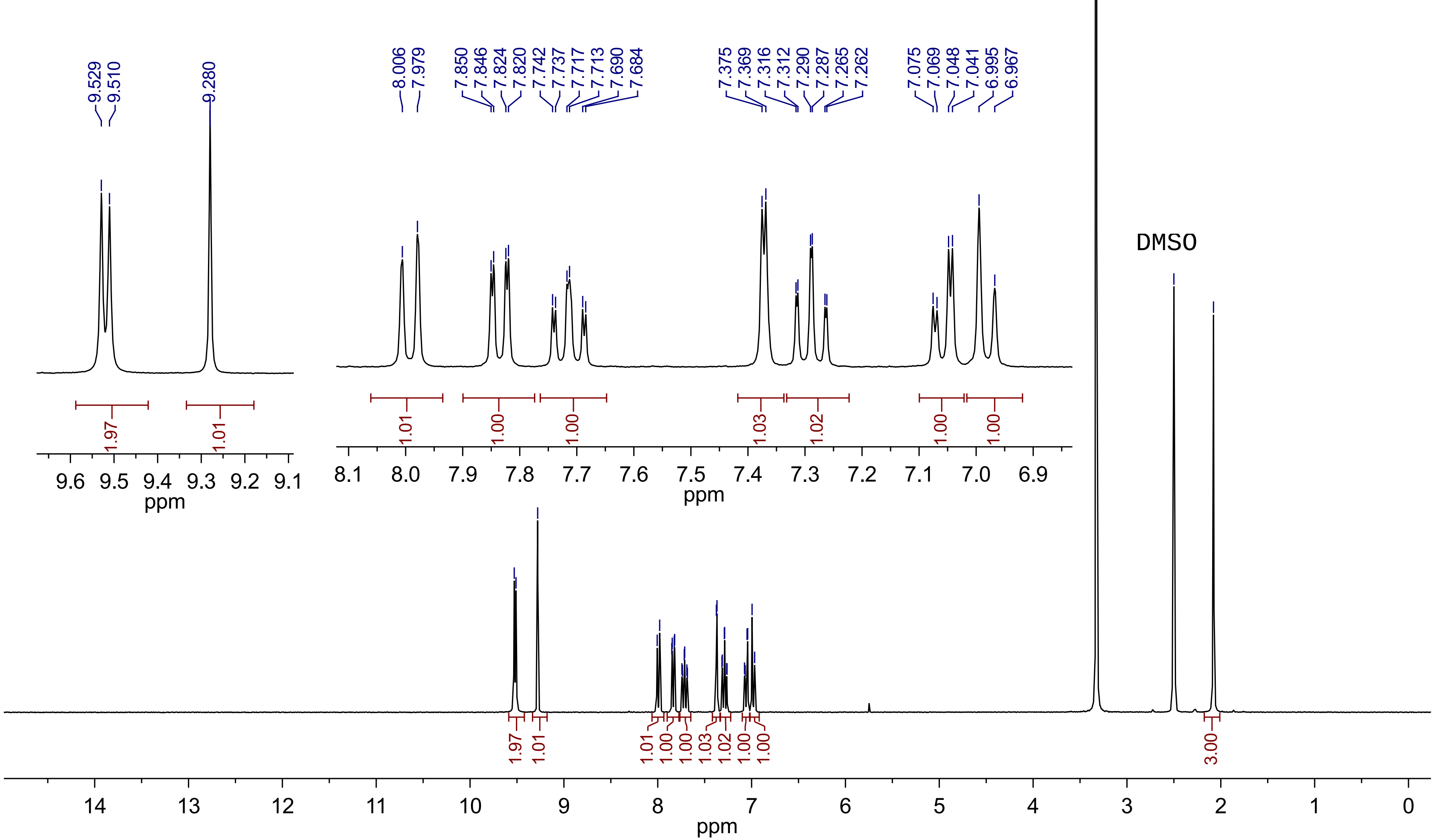

Current Data Parameters	
NAME	Andreas
EXPNO	3
PROCNO	1
**F2 - Acquisition Parameters**	
Date_	20170624
Time	18.38 h
INSTRUM	spect
PROBHD	Z104275_0375 (
PULPROG	zg30
TD	65536
SOLVENT	DMSO
NS	16
DS	2
SWH	6009.615 Hz
FIDRES	0.183399 Hz
AQ	5.4525952 sec
RG	201.81
DW	83.200 usec
DE	6.50 usec
TE	300.5 K
D1	1.00000000 sec
TD0	1
SFO1	300.1318533 MHz
NUC1	1H
P1	14.00 usec
PLW1	7.50000000 W
**F2 - Processing parameters**	
SI	65536
SF	300.1300033 MHz
WDW	EM
SSB	0
LB	0.30 Hz
GB	0
PC	1.00

^{13}C-NMR of 2-((5-((3-hydroxy-4-methylphenyl)amino)-4-oxo-4*H*-1,2,6-thiadiazin-3-yl)amino)-benzonitrile (**13**)

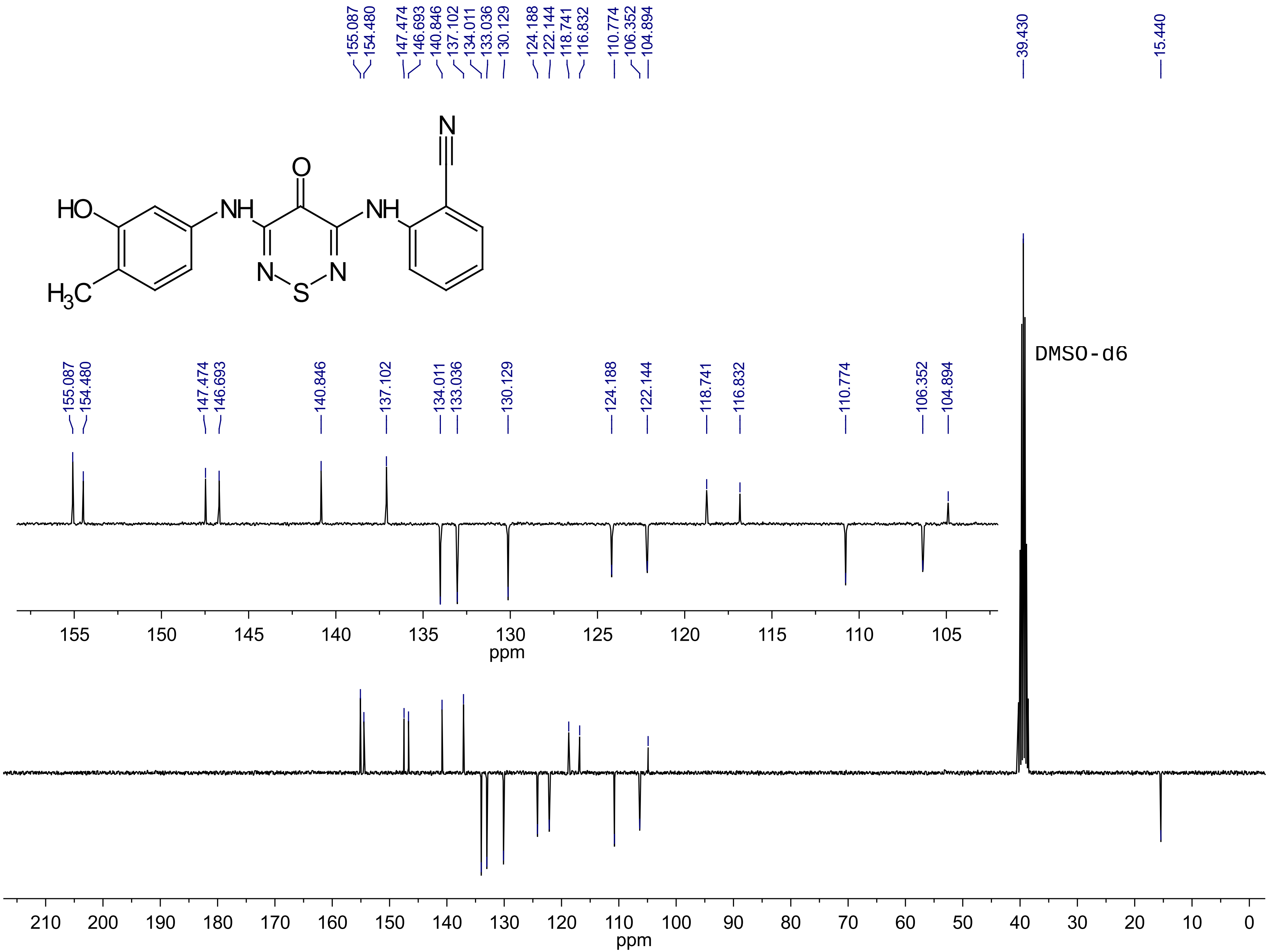

Current Data Parameters	
NAME	Andreas
EXPNO	4
PROCNO	1
F2 - Acquisition Parameters	
Date_	20170625
Time	10.05 h
INSTRUM	spect
PROBHD	Z104275_0375 (
PULPROG	jmod
TD	65536
SOLVENT	DMSO
NS	14336
DS	4
SWH	18115.941 Hz
FIDRES	0.552855 Hz
AQ	1.8087935 sec
RG	201.81
DW	27.600 usec
DE	6.50 usec
TE	301.1 K
CNST2	145.0000000
CNST11	1.0000000
D1	2.00000000 sec
D20	0.00689655 sec
TD0	1
SFO1	75.4752953 MHz
NUC1	13C
P1	10.00 usec
P2	20.00 usec
PLW1	40.05500031 W
SFO2	300.1312005 MHz
NUC2	1H
CPDPRG[2	waltz16
PCPD2	90.00 usec
PLW2	7.50000000 W
PLW12	0.18148001 W
F2 - Processing parameters	
SI	32768
SF	75.4677923 MHz
WDW	EM
SSB	0
LB	1.00 Hz
GB	0
PC	1.40

^{1}H-NMR of 3-((5-((3-hydroxy-4-methylphenyl)amino)-4-oxo-4*H*-1,2,6-thiadiazin-3-yl)amino)-benzonitrile (**14**)

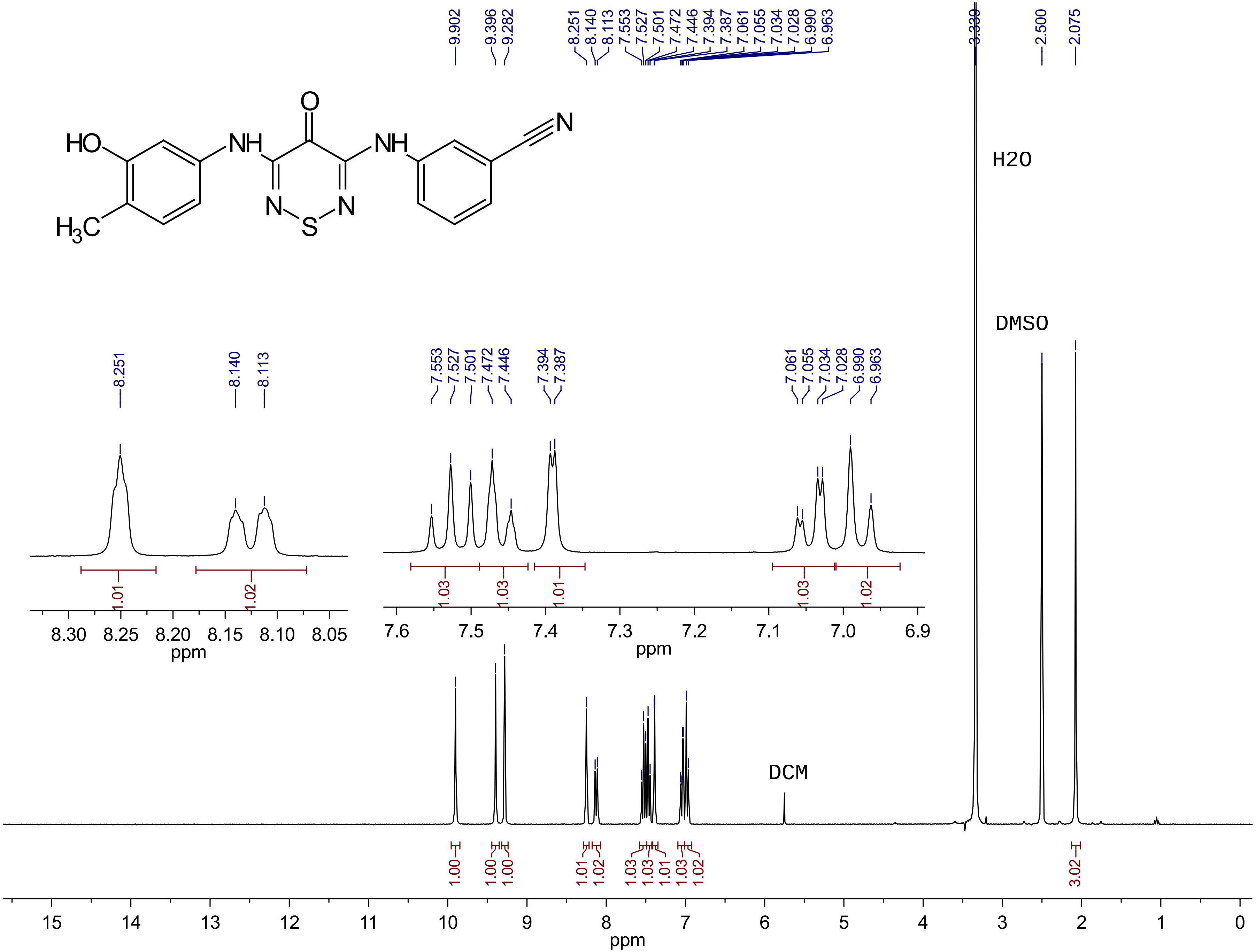

Current Data Parameters
NAME Andreas
EXPNO 10
PROCNO 1

F2 - Acquisition Parameters
Date_ 20170630
Time 20.49 h
INSTRUM spect
PROBHD Z104275_0375 (
PULPROG zg30
TD 65536
SOLVENT DMSO
NS 16
DS 2
SWH 6009.615 Hz
FIDRES 0.183399 Hz
AQ 5.4525952 sec
RG 201.81
DW 83.200 usec
DE 6.50 usec
TE 298.1 K
D1 1.00000000 sec
TD0 1
SFO1 300.1318533 MHz
NUC1 1H
P1 14.00 usec
PLW1 7.50000000 W

F2 - Processing parameters
SI 65536
SF 300.1300030 MHz
WDW EM
SSB 0
LB 0.30 Hz
GB 0
PC 1.00

^{13}C-NMR of 3-((5-((3-hydroxy-4-methylphenyl)amino)-4-oxo-4*H*-1,2,6-thiadiazin-3-yl)amino)-benzonitrile (**14**)

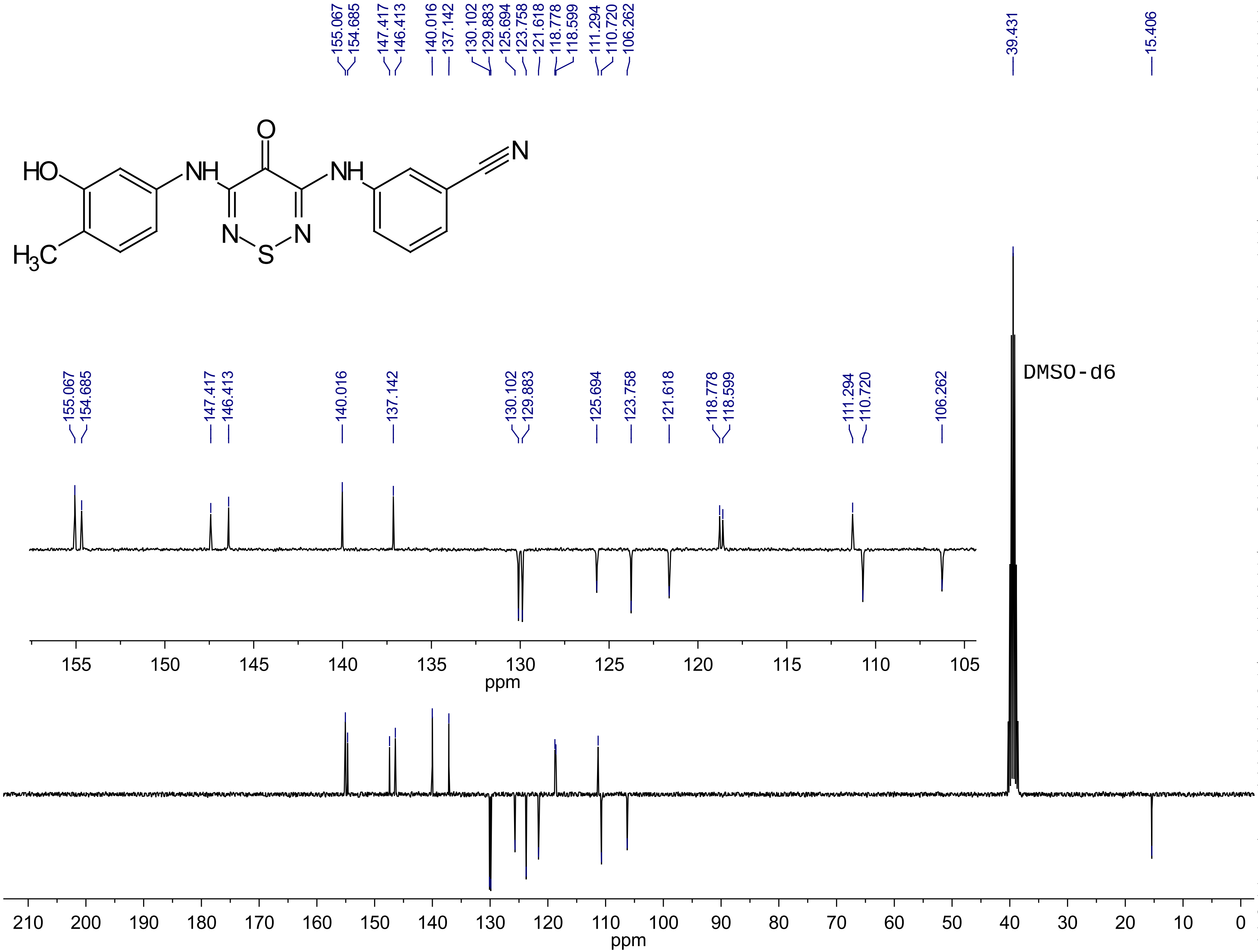

Current Data Parameters
NAME Andreas
EXPNO 12
PROCNO 1
F2 - Acquisition Parameters
Date_ 20170701
Time 7.50 h
INSTRUM spect
PROBHD Z104275_0375 (
PULPROG jmod
TD 65536
SOLVENT DMSO
NS 10240
DS 4
SWH 18115.941 Hz
FIDRES 0.552855 Hz
AQ 1.8087935 sec
RG 201.81
DW 27.600 usec
DE 6.50 usec
TE 298.4 K
CNST2 145.0000000
CNST11 1.0000000
D1 2.00000000 sec
D20 0.00689655 sec
TD0 1
SFO1 75.4752953 MHz
NUC1 13C
P1 10.00 usec
P2 20.00 usec
PLW1 40.05500031 W
SFO2 300.1312005 MHz
NUC2 1H
CPDPRG[2 waltz16
PCPD2 90.00 usec
PLW2 7.50000000 W
PLW12 0.18148001 W
F2 - Processing parameters
SI 32768
SF 75.4677938 MHz
WDW EM
SSB 0
LB 1.00 Hz
GB 0
PC 1.40

^{1}H-NMR of 4-((5-((3-hydroxy-4-methylphenyl)amino)-4-oxo-4*H*-1,2,6-thiadiazin-3-yl)amino)-benzonitrile (**15**)

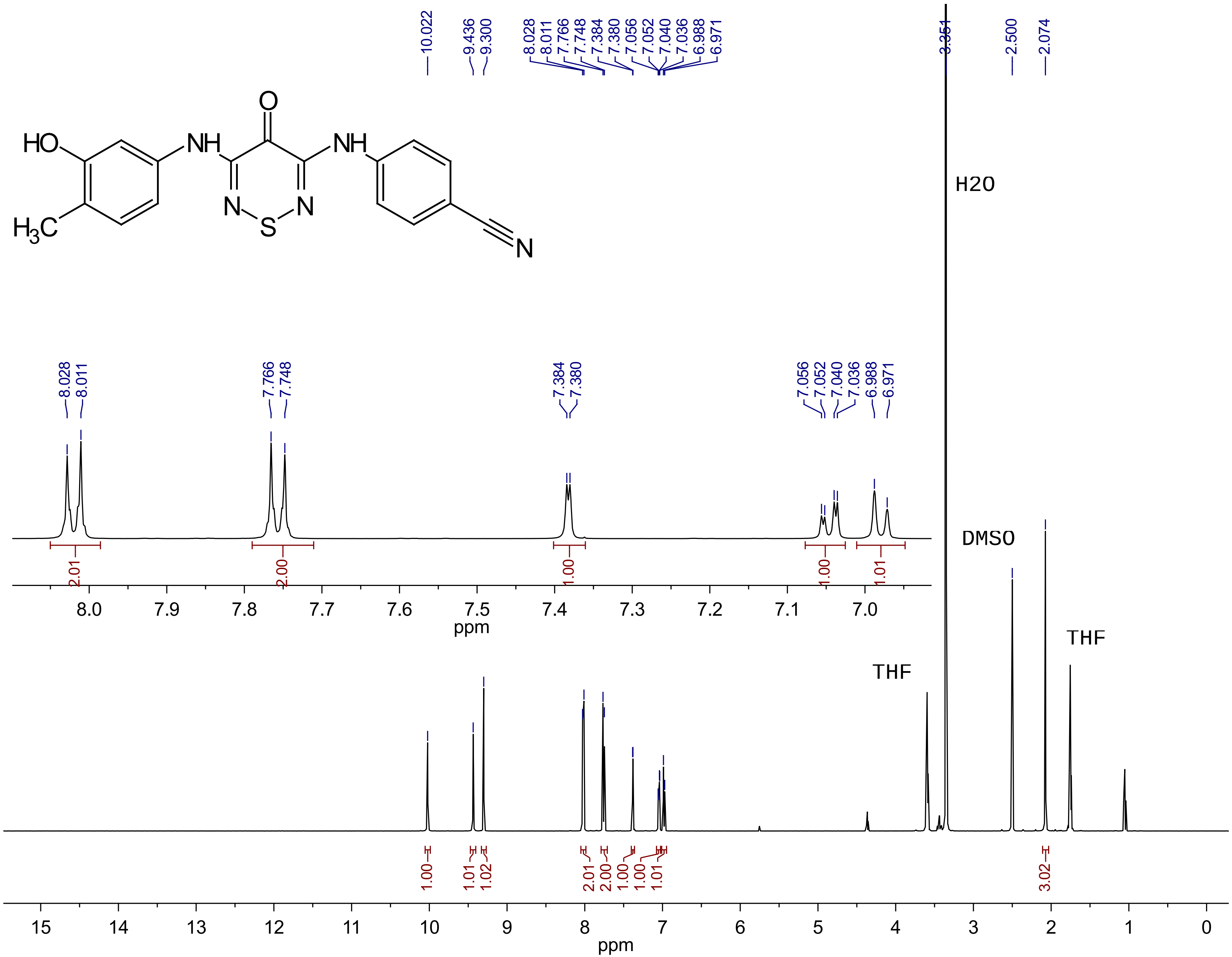

Current Data Parameters
NAME Kalogirou
EXPNO 333
PROCNO 1

F2 - Acquisition Parameters
Date_ 20170613
Time 16.08
INSTRUM spect
PROBHD 5 mm PABBO BB-
PULPROG zg30
TD 65536
SOLVENT DMSO
NS 16
DS 2
SWH 10000.000 Hz
FIDRES 0.152588 Hz
AQ 3.2767999 sec
RG 101
DW 50.000 usec
DE 6.50 usec
TE 295.4 K
D1 1.00000000 sec
TD0 1

======== CHANNEL f1 ========
SFO1 500.0361158 MHz
NUC1 1H
P1 12.00 usec
PLW1 14.50000000 W

F2 - Processing parameters
SI 65536
SF 500.0330320 MHz
WDW EM
SSB 0
LB 0.30 Hz
GB 0
PC 1.00

^{13}C-NMR of 4-((5-((3-hydroxy-4-methylphenyl)amino)-4-oxo-4*H*-1,2,6-thiadiazin-3-yl)amino)-benzonitrile (**15**)

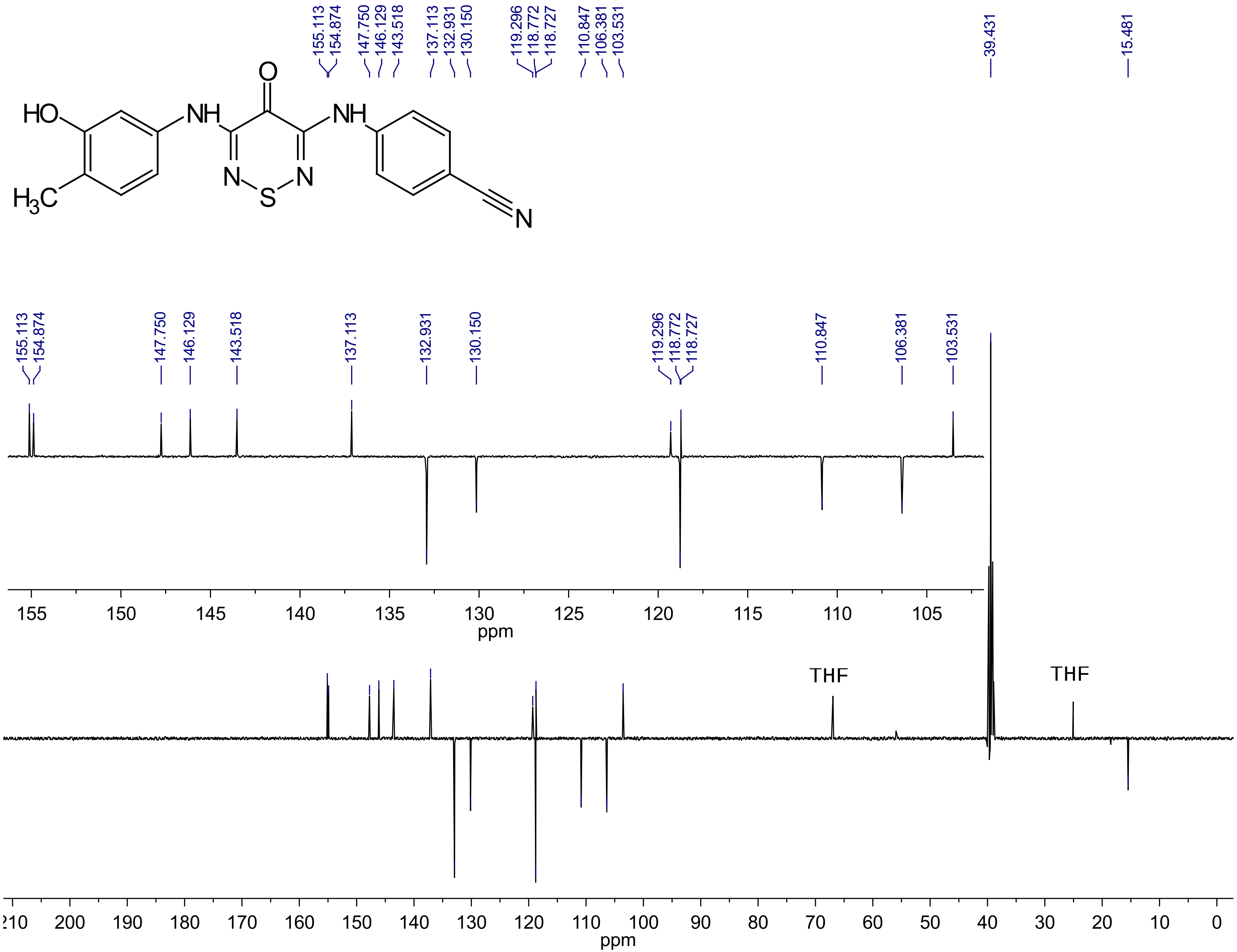

Current Data Parameters
NAME Kalogirou
EXPNO 334
PROCNO 1
F2 - Acquisition Parameters
Date_ 20170613
Time 16.54
INSTRUM spect
PROBHD 5 mm PABBO BB-
PULPROG jmod
TD 65536
SOLVENT DMSO
NS 2902
DS 4
SWH 29761.904 Hz
FIDRES 0.454131 Hz
AQ 1.1010048 sec
RG 2050
DW 16.800 usec
DE 6.50 usec
TE 296.9 K
CNST2 145.0000000
CNST11 1.0000000
D1 2.00000000 sec
D20 0.00689655 sec
TD0 1
======== CHANNEL f1 ========
SFO1 125.7459782 MHz
NUC1 13C
P1 9.00 usec
P2 18.00 usec
PLW1 140.00000000 W
======== CHANNEL f2 ========
SFO2 500.0350280 MHz
NUC2 1H
CPDPRG[2 waltz16
PCPD2 80.00 usec
PLW2 14.50000000 W
PLW12 0.32624999 W
F2 - Processing parameters
SI 32768
SF 125.7334733 MHz
WDW EM
SSB 0
LB 1.00 Hz
GB 0
PC 1.40

^{1}H-NMR of 4-((5-((3-hydroxy-4-methylphenyl)amino)-4-oxo-4*H*-1,2,6-thiadiazin-3-yl)(oxazol-4-ylmethyl)amino)benzamide (**16**)

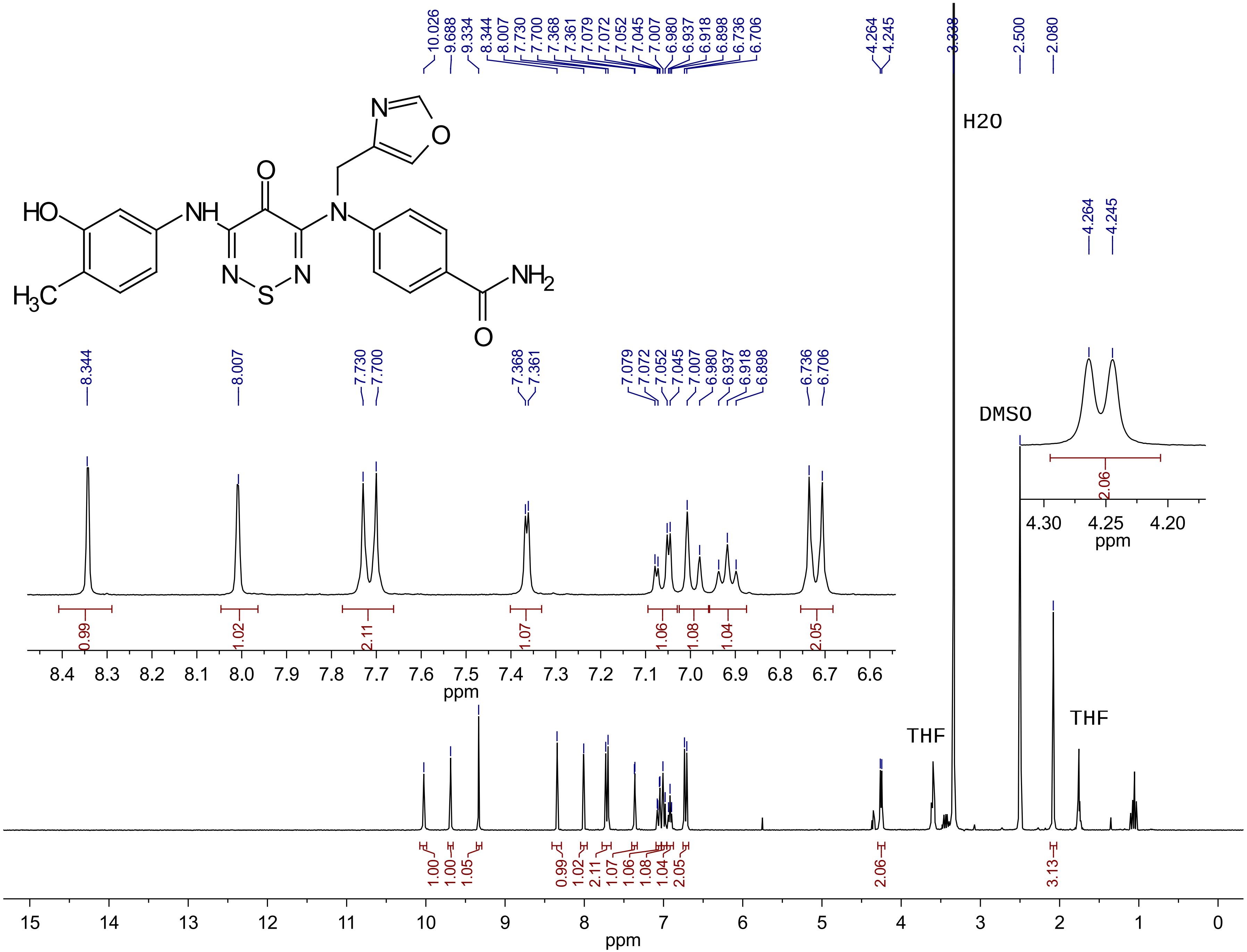

Current Data Parameters
NAME Andreas
EXPNO 15
PROCNO 1

F2 - Acquisition Parameters
Date_ 20170710
Time 18.33 h
INSTRUM spect
PROBHD Z104275_0375 (
PULPROG zg30
TD 65536
SOLVENT DMSO
NS 16
DS 2
SWH 6009.615 Hz
FIDRES 0.183399 Hz
AQ 5.4525952 sec
RG 201.81
DW 83.200 usec
DE 6.50 usec
TE 298.1 K
D1 1.00000000 sec
TD0 1
SFO1 300.1318533 MHz
NUC1 1H
P1 14.00 usec
PLW1 7.50000000 W

F2 - Processing parameters
SI 65536
SF 300.1300029 MHz
WDW EM
SSB 0
LB 0.30 Hz
GB 0
PC 1.00

^{13}C-NMR of 4-((5-((3-hydroxy-4-methylphenyl)amino)-4-oxo-4*H*-1,2,6-thiadiazin-3-yl)(oxazol-4-ylmethyl)amino)benzamide (**16**)

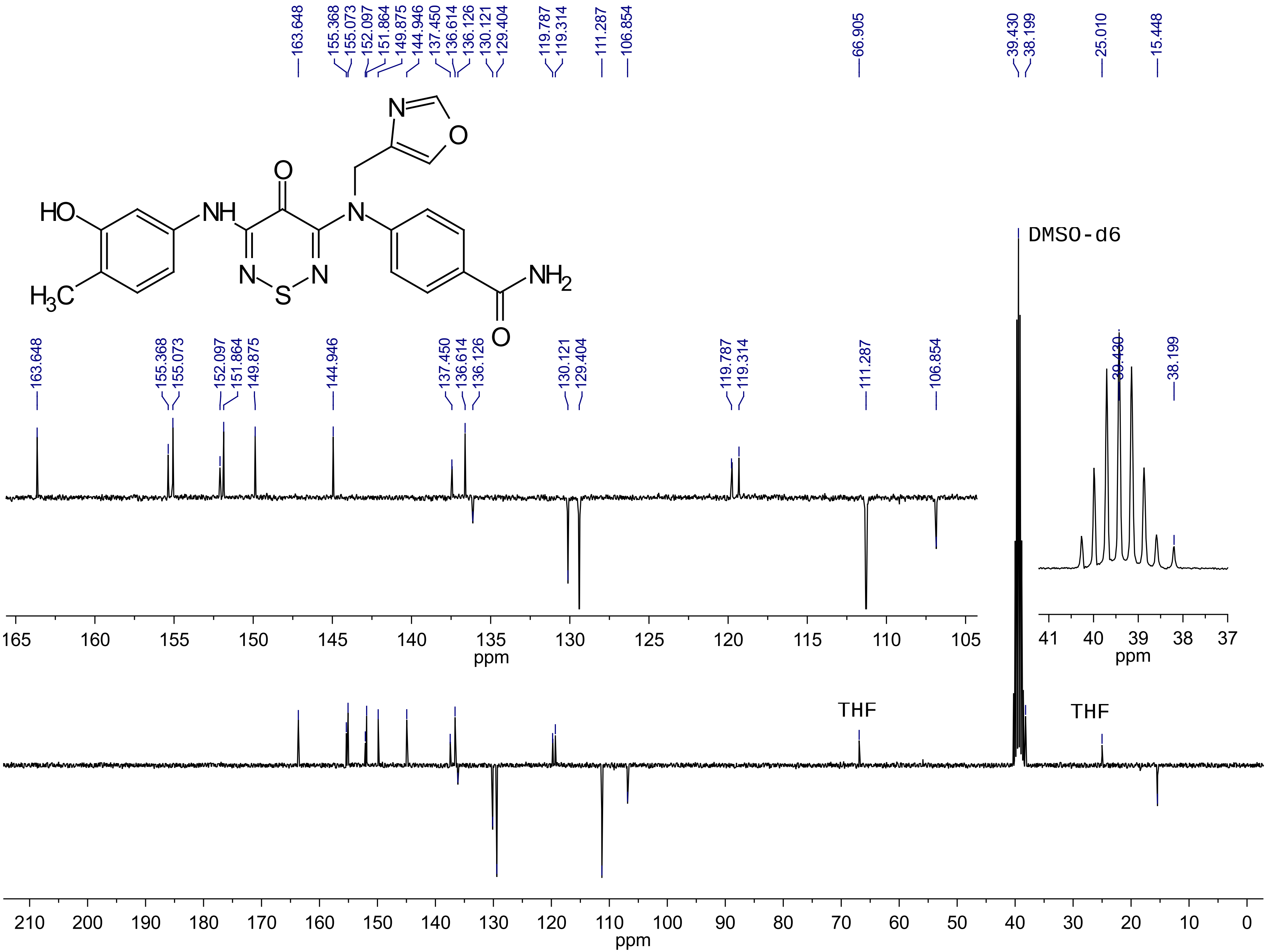

Current Data Parameters	
NAME	Andreas
EXPNO	16
PROCNO	1
F2 - Acquisition Parameters	
Date_	20170711
Time	9.56 h
INSTRUM	spect
PROBHD	Z104275_0375 (
PULPROG	jmod
TD	65536
SOLVENT	DMSO
NS	14336
DS	4
SWH	18115.941 Hz
FIDRES	0.552855 Hz
AQ	1.8087935 sec
RG	201.81
DW	27.600 usec
DE	6.50 usec
TE	298.2 K
CNST2	145.0000000
CNST11	1.0000000
D1	2.00000000 sec
D20	0.00689655 sec
TD0	1
SFO1	75.4752953 MHz
NUC1	13C
P1	10.00 usec
P2	20.00 usec
PLW1	40.05500031 W
SFO2	300.1312005 MHz
NUC2	1H
CPDPRG[2	waltz16
PCPD2	90.00 usec
PLW2	7.50000000 W
PLW12	0.18148001 W
F2 - Processing parameters	
SI	32768
SF	75.4677927 MHz
WDW	EM
SSB	0

^{1}H-NMR of 3-((3-hydroxy-4-methylphenyl)amino)-5-((2-(hydroxymethyl)phenyl)amino)-4*H*-1,2,6-thiadiazin-4-one (**17**)

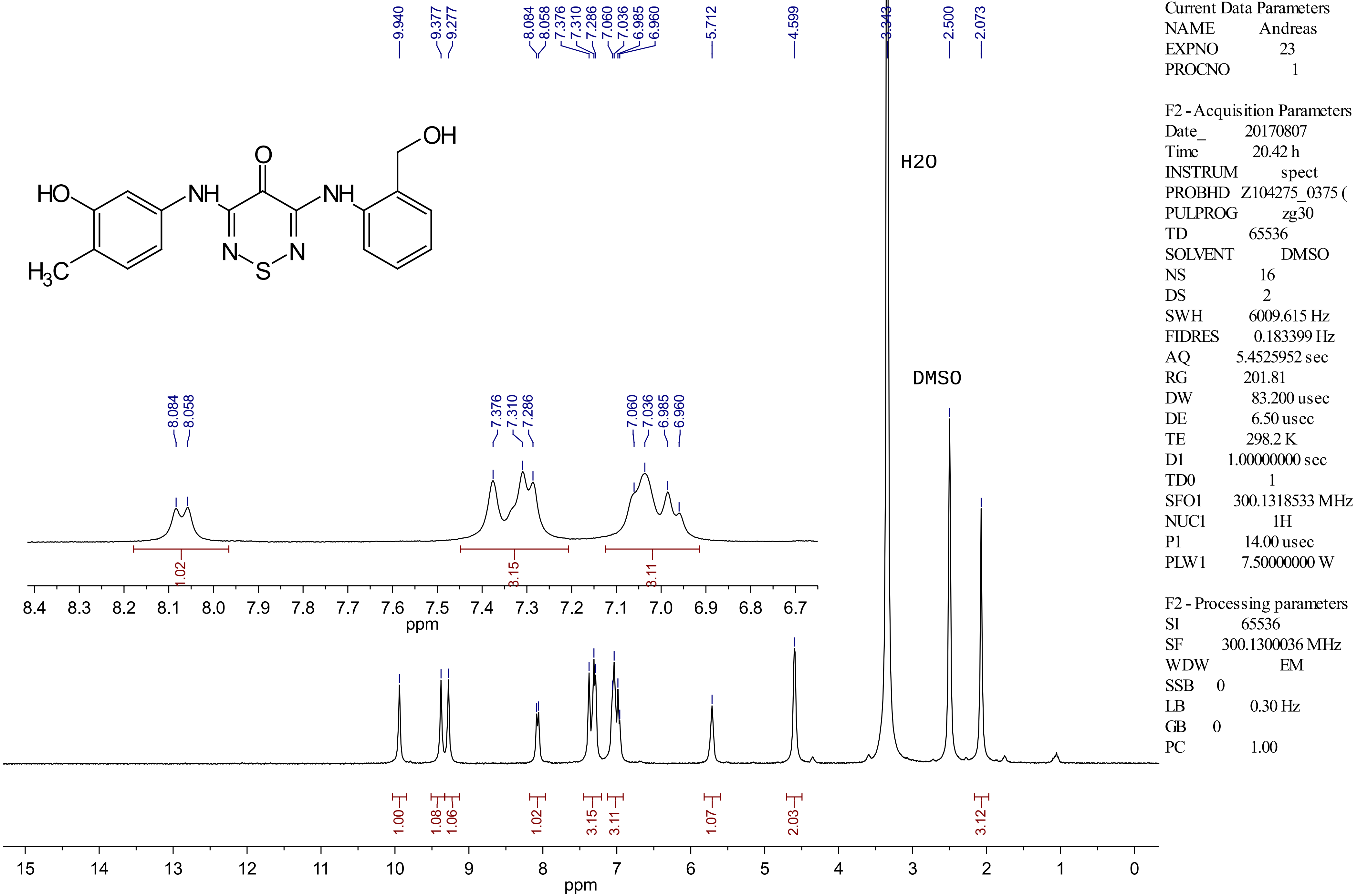

^{13}C-NMR of 3-((3-hydroxy-4-methylphenyl)amino)-5-((2-(hydroxymethyl)phenyl)amino)-4*H*-1,2,6-thiadiazin-4-one (**17**)

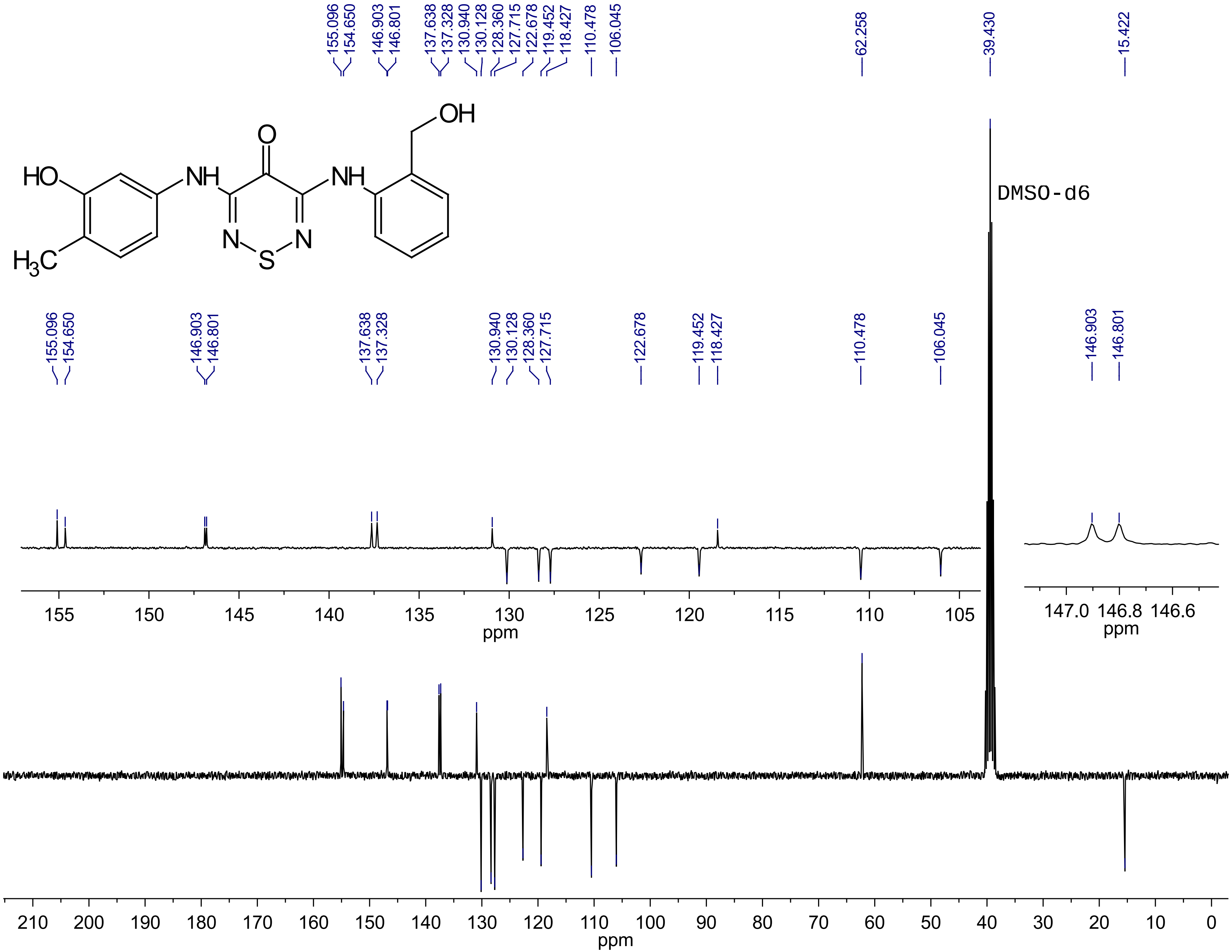

Current Data Parameters
NAME Andreas
EXPNO 24
PROCNO 1
F2 - Acquisition Parameters
Date_ 20170808
Time 9.49 h
INSTRUM spect
PROBHD Z104275_0375 (
PULPROG jmod
TD 65536
SOLVENT DMSO
NS 12228
DS 4
SWH 18115.941 Hz
FIDRES 0.552855 Hz
AQ 1.8087935 sec
RG 201.81
DW 27.600 usec
DE 6.50 usec
TE 298.4 K
CNST2 145.0000000
CNST11 1.0000000
D1 2.00000000 sec
D20 0.00689655 sec
TD0 1
SFO1 75.4752953 MHz
NUC1 13C
P1 10.00 usec
P2 20.00 usec
PLW1 40.05500031 W
SFO2 300.1312005 MHz
NUC2 1H
CPDPRG[2 waltz16
PCPD2 90.00 usec
PLW2 7.50000000 W
PLW12 0.18148001 W
F2 - Processing parameters
SI 32768
SF 75.4677929 MHz
WDW EM
SSB 0
LB 1.00 Hz
GB 0
PC 1.40

^{1}H-NMR of 3-((3-hydroxy-4-methylphenyl)amino)-5-((2-(hydroxymethyl)-3-(trifluoromethyl)-phenyl)-amino)-4*H*-1,2,6-thiadiazin-4-one (**18**)

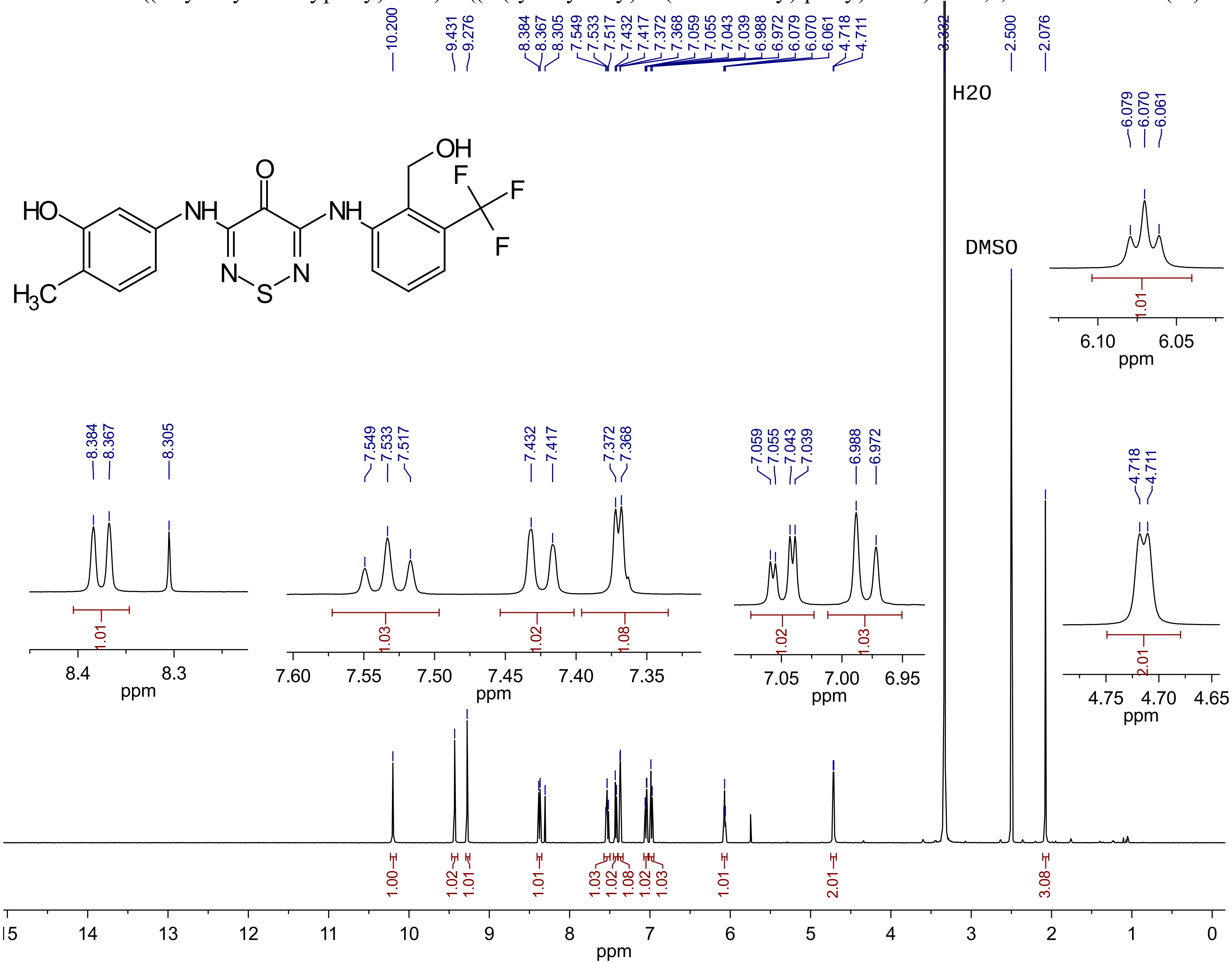

Current Data Parameters
NAME Kalogirou
EXPNO 394
PROCNO 1
F2 - Acquisition Parameters
Date_ 20170813
Time 5.09
INSTRUM spect
PROBHD 5 mm PABBO BB-
PULPROG zg30
TD 65536
SOLVENT DMSO
NS 16
DS 2
SWH 10000.000 Hz
FIDRES 0.152588 Hz
AQ 3.2767999 sec
RG 128
DW 50.000 usec
DE 6.50 usec
TE 300.8 K
D1 1.00000000 sec
TD0 1
======== CHANNEL f1 ========
SFO1 500.0361158 MHz
NUC1 1H
P1 12.00 usec
PLW1 14.50000000 W
F2 - Processing parameters
SI 65536
SF 500.0330324 MHz
WDW EM
SSB 0
LB 0.30 Hz
GB 0
PC 1.00

^{13}C-NMR of 3-((3-hydroxy-4-methylphenyl)amino)-5-((2-(hydroxymethyl)-3-(trifluoromethyl)-phenyl)-amino)-4*H*-1,2,6-thiadiazin-4-one (**18**)

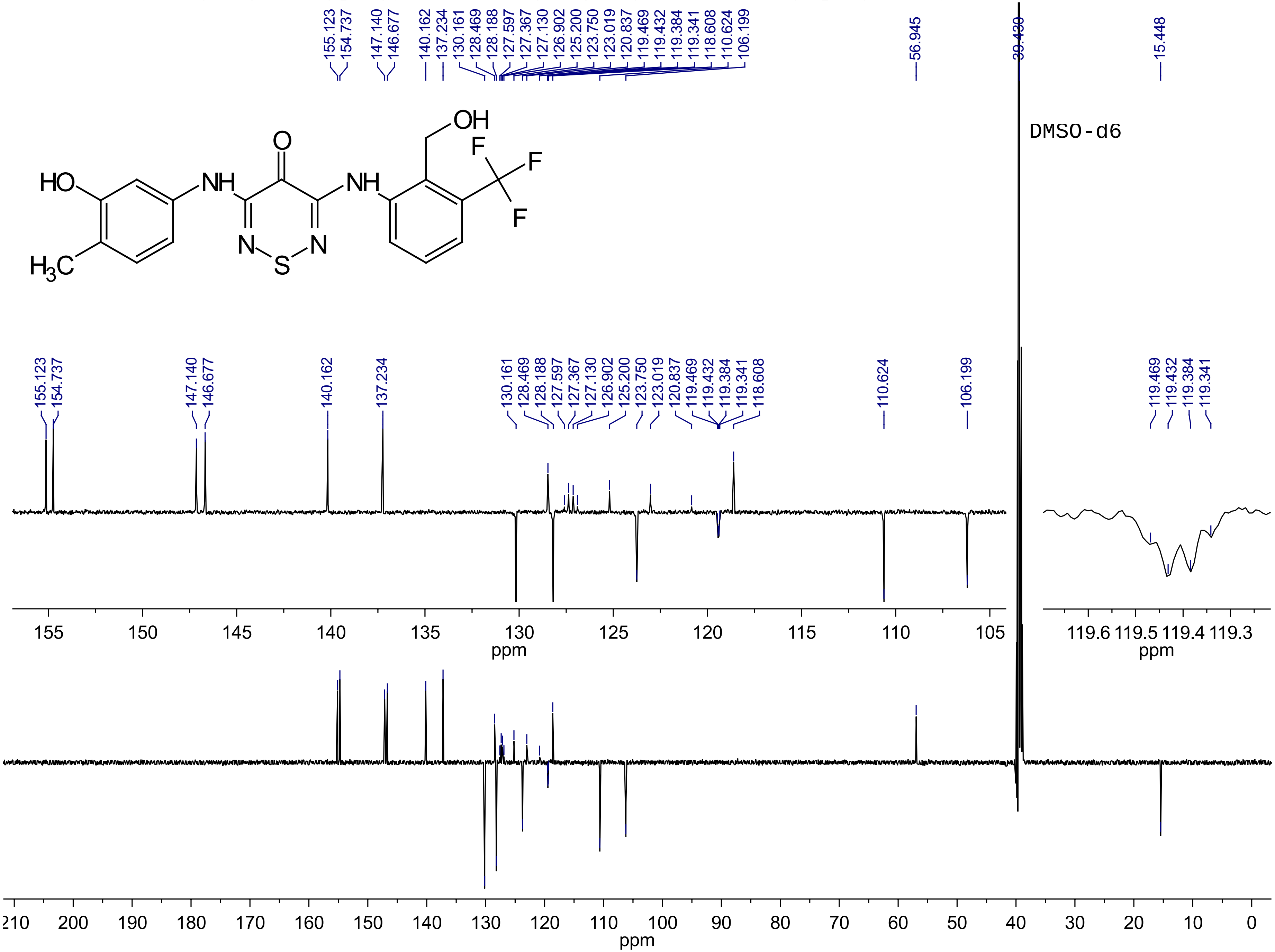

Current Data Parameters
NAME Kalogirou
EXPNO 395
PROCNO 1

F2 - Acquisition Parameters
Date_ 20170813
Time 15.55
INSTRUM spect
PROBHD 5 mm PABBO BB-
PULPROG jmod
TD 65536
SOLVENT DMSO
NS 12288
DS 4
SWH 29761.904 Hz
FIDRES 0.454131 Hz
AQ 1.1010048 sec
RG 1820
DW 16.800 usec
DE 6.50 usec
TE 298.4 K
CNST2 145.0000000
CNST11 1.0000000
D1 2.00000000 sec
D20 0.00689655 sec
TD0 1

======== CHANNEL f1 ========
SFO1 125.7459782 MHz
NUC1 13C
P1 9.00 usec
P2 18.00 usec
PLW1 140.00000000 W

======== CHANNEL f2 ========
SFO2 500.0350280 MHz
NUC2 1H
CPDPRG[2 waltz16
PCPD2 80.00 usec
PLW2 14.50000000 W
PLW12 0.32624999 W

F2 - Processing parameters
SI 32768
SF 125.7334754 MHz
WDW EM
SSB 0
LB 1.00 Hz
GB 0
PC 1.40

^{1}H-NMR of 2-cyclopentyl-4-(5-((3-hydroxy-4-methylphenyl)amino)-4-oxo-4*H*-1,2,6-thiadiazin-3-yl)-benzoic acid (**19**)

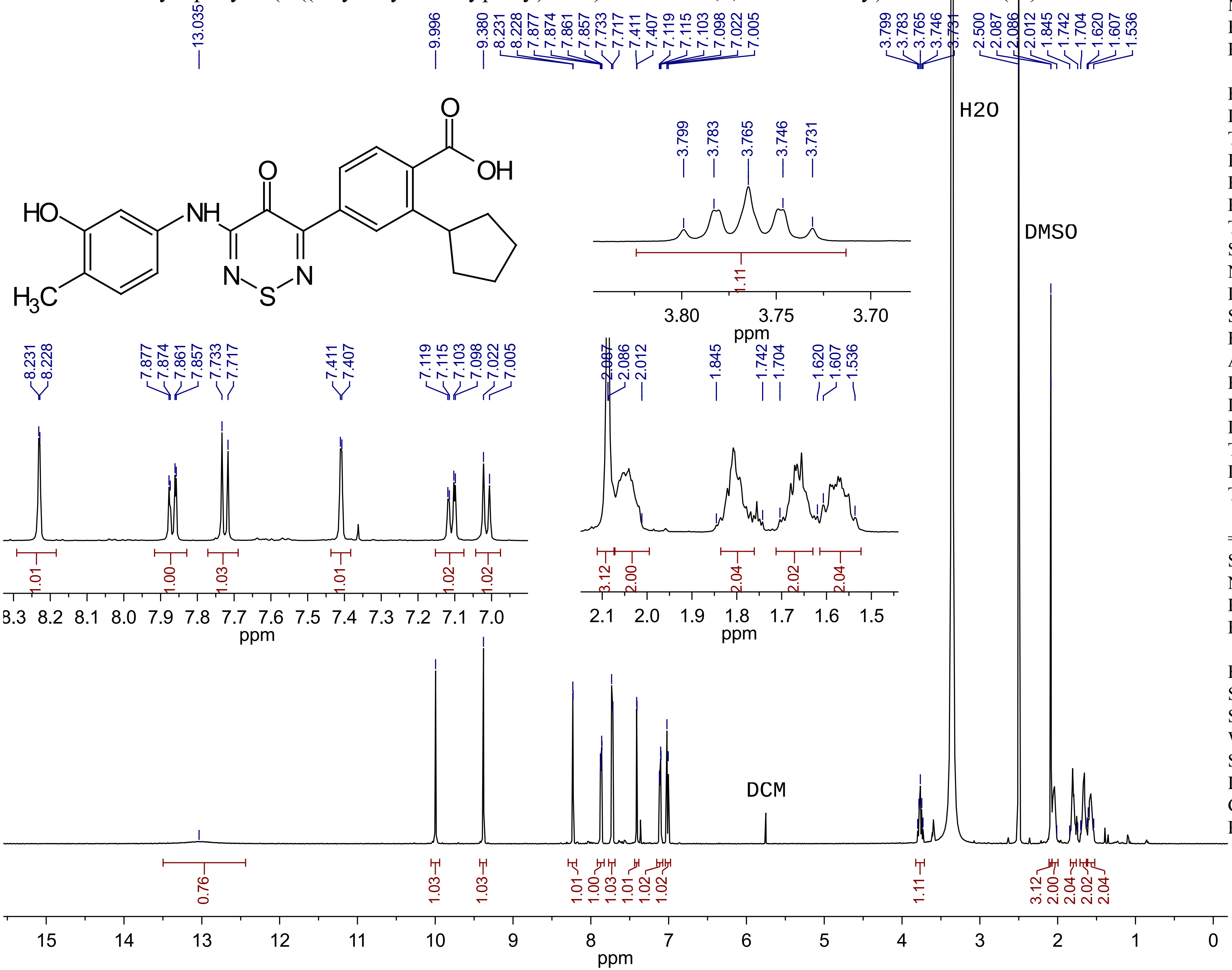

Current Data Parameters
NAME Kalogirou
EXPNO 410
PROCNO 1

F2 - Acquisition Parameters
Date_ 20170918
Time 16.26
INSTRUM spect
PROBHD 5 mm PABBO BB-
PULPROG zg30
TD 65536
SOLVENT DMSO
NS 16
DS 2
SWH 10000.000 Hz
FIDRES 0.152588 Hz
AQ 3.2767999 sec
RG 101
DW 50.000 usec
DE 6.50 usec
TE 296.9 K
D1 1.00000000 sec
TD0 1

======== CHANNEL f1 ========
SFO1 500.0361158 MHz
NUC1 1H
P1 12.00 usec
PLW1 14.50000000 W

F2 - Processing parameters
SI 65536
SF 500.0330320 MHz
WDW EM
SSB 0
LB 0.30 Hz
GB 0
PC 1.00

^{13}C-NMR of 2-cyclopentyl-4-(5-((3-hydroxy-4-methylphenyl)amino)-4-oxo-4*H*-1,2,6-thiadiazin-3-yl)-benzoic acid (**19**)

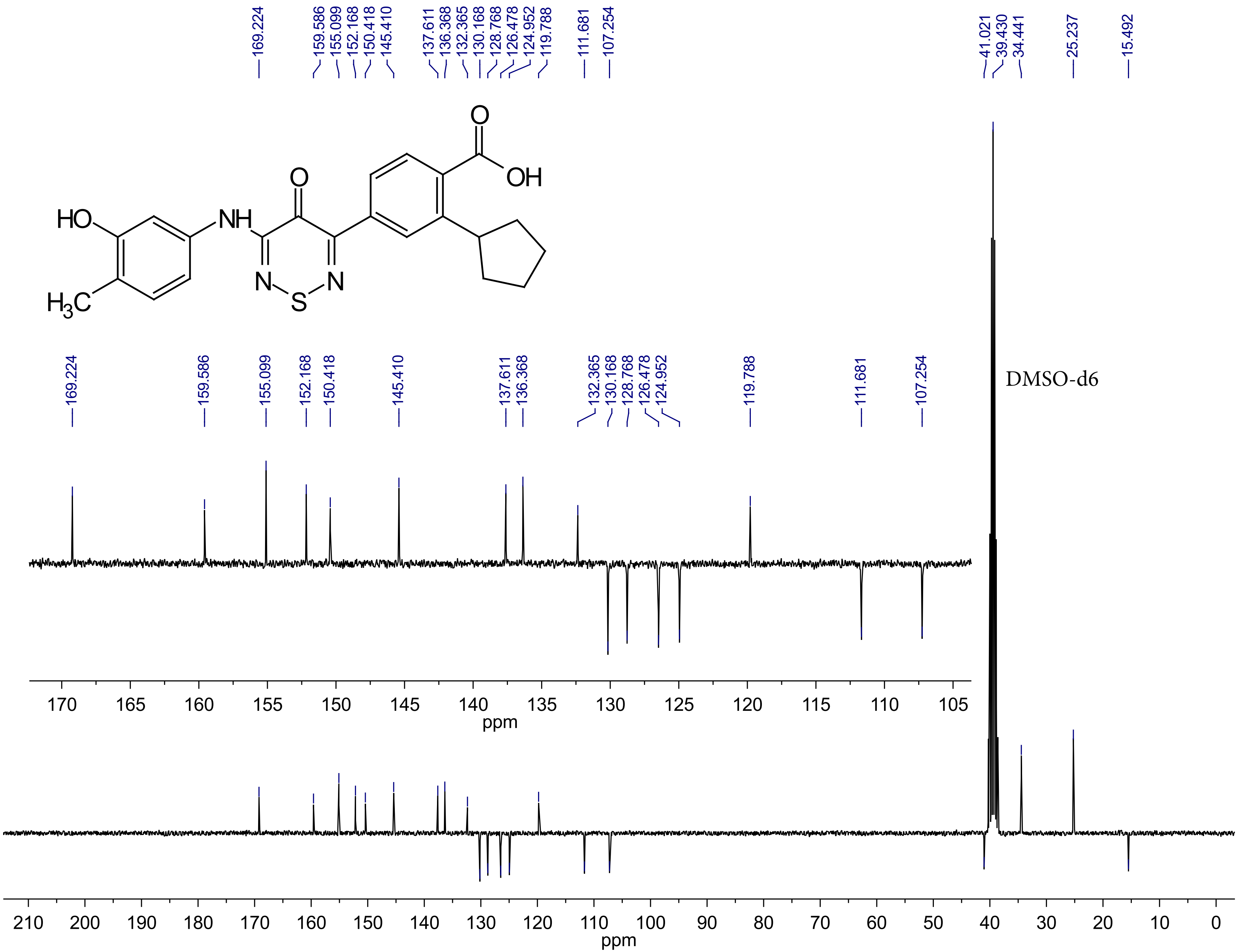

Current Data Parameters
NAME Andreas
EXPNO 46
PROCNO 1
F2 - Acquisition Parameters
Date_ 20170919
Time 9.39 h
INSTRUM spect
PROBHD Z104275_0375 (
PULPROG jmod
TD 65536
SOLVENT DMSO
NS 12288
DS 4
SWH 18115.941 Hz
FIDRES 0.552855 Hz
AQ 1.8087935 sec
RG 201.81
DW 27.600 usec
DE 6.50 usec
TE 298.6 K
CNST2 145.0000000
CNST11 1.0000000
D1 2.00000000 sec
D20 0.00689655 sec
TD0 1
SFO1 75.4752953 MHz
NUC1 13C
P1 10.00 usec
P2 20.00 usec
PLW1 40.05500031 W
SFO2 300.1312005 MHz
NUC2 1H
CPDPRG[2 waltz16
PCPD2 90.00 usec
PLW2 7.50000000 W
PLW12 0.18148001 W
F2 - Processing parameters
SI 32768
SF 75.4677915 MHz
WDW EM
SSB 0
LB 1.00 Hz
GB 0
PC 1.40
